# Supplementary material for: Health Outcomes, Pathogenesis and Epidemiology of Severe Acute Malnutrition (HOPE-SAM): rationale and methods of a longitudinal observational study
Source: BMJ Open. 2019 Feb 1;9(1):e023077. doi: 10.1136/bmjopen-2018-023077 (PMC6361330; doi:10.1136/bmjopen-2018-023077)
Supplement: Supplementary data [file bmjopen-2018-023077supp001.pdf]

Supplementary Table 1: Assays undertaken on stored samples for children in the observational cohort and immunology substudy.

Sample type	Assay (method)	Location of work	Study subjects	Baseline	Discharge	Week 12	Week 24	Week 48
Blood	HIV testing (rapid antibody test algorithm if >18 mo old or HIV DNA PCR <18 mo old)[1]	TROPGAN, Zvitambo or clinical sites	All	X				
Blood	CD4 count (flow cytometry or PIMA)[1]	TROPGAN, Zvitambo or clinical sites	All HIV-positive	X	X	X	X	X
Plasma	HIV viral load (real-time PCR)[1]	TROPGAN, Zvitambo or clinical sites	All HIV-positive	X	X	X	X	X
Plasma	C-reactive protein (ELISA)	TROPGAN, Zvitambo	All	X	X	X	X	X
Plasma	Albumin (ELISA)	TROPGAN, Zvitambo	All	X	X	X	X	X
Plasma	Lipopolysaccharide (LAL assay)	TROPGAN and Zvitambo	Subgroup[3]	X	X	X	X	X
Plasma	Lipopolysaccharide binding protein (LBP)	TROPGAN and Zvitambo	Subgroup[3]	X	X	X	X	X
Plasma	sCD14 (ELISA)	TROPGAN and Zvitambo	Subgroup[3]	X	X	X	X	X
Plasma	sCD163 (ELISA)	TROPGAN and Zvitambo	Subgroup[3]	X	X	X	X	X
Plasma	IL-6, TNF-alpha, IL-1β (ELISA) and/or multiplex cytokines	TROPGAN and Zvitambo	Subgroup[3]	X	X	X	X	X
Plasma	Total PAMP activity (THP1 reporter cell line[2])	TROPGAN and Zvitambo	Subgroup[3]	X	X	X	X	X
Whole	Molecular techniques	QMUL, London[2]	Subgroup[3]	X	X	X	X	X

blood	for bacterial detection (broad-range and specific PCR and next-generation sequencing)							
**Immunology substudy only**								
Whole blood	*In vitro* binding to bacterial products, cytokine expression and cellular responses to PAMP stimulation[4]	TROPGAN, Zvitambo	All	X	X	X	X	X
Plasma	Co-culture with healthy immune cells[5]	QMUL, London	All	X	X	X	X	X

[1]If HIV test, CD4 and viral load have already been conducted as part of routine clinical care, they will not be repeated on the research sample.
[2]THP1 reporter cells are derived from THP1, a human monocytic cell line that naturally expresses many pattern recognition receptors (PRR). The cell line stably expresses an NF-κB/AP-1 inducible reporter (SEAP) system to facilitate the monitoring of PRR-induced NF-κB/AP-1 activation.
[3]Assays will be undertaken in a subgroup of children, using a case-control or case-cohort design to evaluate the impact of biomarkers on immune activation and mortality.
[4]Whole blood will be stimulated with pathogen-associated molecular patterns (PAMP) in culture plates and bacterial antigens labelled with fluorescent tags in test tubes, and incubated for 1-24hr. Supernatant will be removed and stored at -80C for subsequent analysis of pro- and anti-inflammatory cytokines, and cells will be fixed for subsequent analysis of bacterial binding, cellular activation, proliferation and cytokine elaboration by flow cytometry.
[5]To determine the effect of the systemic milieu on healthy immune cell function, plasma samples will be transported to the Blizard Institute, QMUL and co-cultured with healthy immune cells, which will be functionally analysed via multi-parameter flow cytometry in the Flow Cytometry Core Facility.

IFABP: Intestinal fatty acid binding protein; ELISA: Enzyme-linked immunosorbent assay; GLP-2: glucagon-like peptide 2; sCD14: soluble CD14; sCD163: soluble CD163; PAMP: pathogen-associated molecular pattern; QMUL: Queen Mary University of London; CRP: C-reactive protein; LAL: limulus amoebocyte lysate assay.

Supplementary table 2: Additional laboratory analyses for enteropathy substudy

Sample type	Assay (method)	Location of work	Study groups[1]	Baseline	Discharge	Week 12	Week 24	Week 48
Urine	Lactulose-mannitol ratio (mass spectrometry)	Orgeon Analytics, USA	A, B, C, D (all)	X	X	X		X
Stool	Neopterin, myeloperoxidase, alpha-1 antitrypsin and REG-1B (ELISA)	TROPGAN and Zvitambo	A, B, C, D (all)	X	X	X	X	X
Plasma	I-FABP (ELISA)	TROPGAN and Zvitambo	A, B, C, D (all)	X	X	X	X	X
Plasma	GLP-2	TROPGAN and Zvitambo	A, B, C, D (all)	X	X	X	X	X
Plasma	Citrulline (mass spectrometry)	Imperial College London	A, B, C, D (all)	X	X	X	X	X
Plasma	Kynurenine:tryptophan ratio and metabolites along tryptophan pathway (mass spectrometry)	Imperial College London	A, B, C, D (all)	X	X	X	X	X
Stool	Microbiome analysis[2]	BCCDC, Vancouver	A, B, C, D (all)	X	X	X	X	X
Stool	*Helicobacter pylori* antigen	TROPGAN and Zvitambo	A, B, C, D (all)	X				
Gastric juice	Culture and molecular techniques for bacterial detection (broad-range and specific PCR and next-generation sequencing)	QMUL, London	Subgroup of A, C (n=50 per group)	X				

RNA extracted from PAXGene tubes	Gene expression analysis (RNASeq)	QMUL, London	A, B, C, D (all)	X	X			
Plasma and urine	Targeted and untargeted metabolic phenotyping	Imperial College London	A, B, C, D (all)	X	X	X	X	X

[1]Enteropathy substudy groups: Group A:,HIV-positive children with severe acute malnutrition; Group B: HIV-positive well-nourished controls; Group C: HIV-negative children with severe acute malnutrition; Group D: HIV-negative well-nourished controls.

Note that controls only have blood taken at baseline.

[2]For microbiome analyses,total DNA and/or RNA will be extracted from stool samples and used as template for next generation sequencing library preparation and for quantitative polymerase chain reaction (qPCR), then sequenced via whole metagenome shotgun sequencing

QMUL: Queen Mary University of London; BCCDC: British Columbia Centre for Disease Control.
