# Supplementary material for: Health Outcomes, Pathogenesis and Epidemiology of Severe Acute Malnutrition (HOPE-SAM): rationale and methods of a longitudinal observational study
Source: BMJ Open. 2019 Feb 1;9(1):e023077. doi: 10.1136/bmjopen-2018-023077 (PMC6361330; doi:10.1136/bmjopen-2018-023077)
Supplement: Author's manuscript [file bmjopen-2018-023077.draft_revisions.pdf]

BMJ Open is committed to open peer review. As part of this commitment we make the peer review history of every article we publish publicly available.

When an article is published we post the peer reviewers' comments and the authors' responses online. We also post the versions of the paper that were used during peer review. These are the versions that the peer review comments apply to.

The versions of the paper that follow are the versions that were submitted during the peer review process. They are not the versions of record or the final published versions. They should not be cited or distributed as the published version of this manuscript.

BMJ Open is an open access journal and the full, final, typeset and author-corrected version of record of the manuscript is available on our site with no access controls, subscription charges or pay-per-view fees (http://bmjopen.bmj.com).

If you have any questions on BMJ Open's open peer review process please email info.bmjopen@bmj.com

BMJ Open

Health Outcomes, Pathogenesis and Epidemiology of Severe Acute Malnutrition (HOPE-SAM): rationale and methods of a longitudinal observational study

Journal:	*BMJ Open*
Manuscript ID	bmjopen-2018-023077
Article Type:	Protocol
Date Submitted by the Author:	21-Mar-2018
Complete List of Authors:	Bwakura-Dangarembizi, Mutsa; Paediatrics and Child Health<br>Amadi, Beatrice; 2Tropical Gastroenterology and Nutrition Group, University of Zambia, Lusaka, Zambia<br>Bourke, Claire ; Blizard Institute, Queen Mary University of London, UK<br>Robertson, Ruairi; Blizard Institute, Queen Mary University of London, UK<br>Mwapenya, Benjamin; Zvitambo Institute for Maternal and Child Health Research, Harare, Zimbabwe<br>Chandwe, Kanta; Tropical Gastroenterology and Nutrition Group, University of Zambia, Lusaka, Zambia<br>Kapoma, Chanda; Tropical Gastroenterology and Nutrition Group, University of Zambia, Lusaka, Zambia, Paediatrics; Livingstone Central Hospital, Paediatrics<br>Chifunda, Kapula; Tropical Gastroenterology and Nutrition Group, University of Zambia, Lusaka, Zambia, paediatrics and child health<br>Majo, Florence; Zvitambo Institute for Maternal and Child Health Research, Harare, Zimbabwezi<br>Ngosa, Deophine; Tropical Gastroenterology and Nutrition Group, University of Zambia, Lusaka, Zambia<br>Chakara, Pamela; Zvitambo Institute for Maternal and Child Health Research, Harare, Zimbabwe<br>Chulu, Nivea; 2Tropical Gastroenterology and Nutrition Group, University of Zambia, Lusaka, Zambia<br>Masimba, Faithfull; Zvitambo Institute for Maternal and Child Health Research, Harare, Zimbabwe<br>Mapurisa, Idah; Zvitambo Institute for Maternal and Child Health Research, Harare, Zimbabwe<br>Besa, Ellen; Zvitambo Institute for Maternal and Child Health Research, Harare, Zimbabwe<br>Mutasa, Kuda; Zvitambo Institute for Maternal and Child Health Research, Harare, Zimbabwe<br>Mwakamui, Simutanyi; 2Tropical Gastroenterology and Nutrition Group, University of Zambia, Lusaka, Zambia<br>Runodamoto, Thompson ; Zvitambo Institute for Maternal and Child Health Research, Harare, Zimbabwe<br>Humphrey, Jean; Zvitambo Institute for Maternal and Child Health Research, Harare, Zimbabwe<br>Ntozini, Robert; Zvitambo Institute, Biostatistics & IT<br>Wells, Jonathan C. K.; Royal Cornwall Hosp NHS Trust

Manges, Amee; University of British Columbia
Swann, Jon; Imperial College London
Walker, Sarah; MRC Clinical Trials Unit at UCL
Nathoo, Kusum; University of Zimbabwe, College of Health Sciences
Kelly, Paul; Barts and The London School of Medicine
Prendergast, Andrew ; Queen Mary University of London,

Keywords: Malnutrition, HIV, Africa, mortality, microbiota, enteropathy

SCHOLARONE™
Manuscripts

Health Outcomes, Pathogenesis and Epidemiology of Severe Acute Malnutrition (HOPE-SAM): rationale and methods of a longitudinal observational study

Mutsa Bwakura-Dangarembizi[1], Beatrice Amadi[2], Claire D Bourke[3], Ruairi C Robertson[3], Benjamin Mwapenya[4], Kanta Chandwe[2], Chanda Kapoma[2], Kapula Chifunda[2], Florence Majo[4], Deophine Ngosa[2], Pamela Chakara[4], Nivea Chulu[2], Faithful Masimba[4], Idah Mapurisa[4], Ellen Besa[2], Kuda Mutasa[4], Simutanyi Mwakamui[2], Thompson Runodamoto[4], Jean H Humphrey[4], Robert Ntozini[4], Jonathan Wells[5], Amee R Manges[6], Jonathan Swann[7], A Sarah Walker[8], Kusum J Nathoo[1], Paul Kelly[2,3], Andrew J Prendergast[3,4] for the HOPE-SAM Study Team[9].

[1]Department of Paediatrics and Child Health, University of Zimbabwe College of Health Sciences, Harare, Zimbabwe

[2]Tropical Gastroenterology and Nutrition Group, University of Zambia, Lusaka, Zambia

[3]Blizard Institute, Queen Mary University of London, UK

[4]Zvitambo Institute for Maternal and Child Health Research, Harare, Zimbabwe

[5]UCL Great Ormond Street Institute of Child Health, London, UK

[6]University of British Columbia, Vancouver, Canada

[7]Imperial College London, UK

[8]MRC Clinical Trials Unit at UCL, London, UK

[9]Other members of the HOPE-SAM study team are listed in the Acknowledgements section.

Corresponding author: Dr Mutsa Bwakura-Dangarembizi, Senior Lecturer, Department of Paediatrics and Child Health, University of Zimbabwe College of

For peer review only

Health Sciences, Box A178, Avondale, Harare, Zimbabwe. Email:

mbwakura@medsch.uz.ac.zw

Tel +263 772 601 735. Fax +263 4 700 877.

Word count 5926

Abstract 261

Key words: Malnutrition, HIV, Africa, mortality, microbiota, enteropathy, immunology

For peer review only

ABSTRACT

Introduction

Mortality among children hospitalised for complicated severe acute malnutrition (SAM) remains high despite the implementation of WHO guidelines, particularly in settings of high HIV prevalence. Children continue to be at high risk of morbidity, mortality and relapse after discharge from hospital although long-term outcomes are not well documented. Better understanding the pathogenesis of SAM and the factors associated with poor outcomes may inform new therapeutic interventions.

Methods and analysis

The Health Outcomes, Pathogenesis and Epidemiology of Severe Acute Malnutrition (HOPE-SAM) study is a longitudinal observational cohort that aims to evaluate the short- and long-term clinical outcomes of HIV-infected and HIV-uninfected children with complicated SAM, and to identify the risk factors at admission and discharge from hospital that independently predict poor outcomes. Children aged 0-59mo hospitalised for SAM are being enrolled at three tertiary hospitals in Harare, Zimbabwe, and Lusaka, Zambia. Longitudinal mortality, morbidity and nutritional data are being collected at admission, discharge and for 48 weeks post-discharge. Nested laboratory substudies are exploring the role of enteropathy, gut microbiota, metabolomics and cellular immune function in the pathogenesis of SAM using stool, urine and blood collected from participants.

Ethics and dissemination

The study is approved by the local and international institutional review boards in the participating countries (the Joint Research Ethics Committee of the University of Zimbabwe, Medical Research Council of Zimbabwe and University of Zambia Biomedical Research Ethics Committee) and the study sponsor (Queen Mary University of London). Caregivers provide written informed consent for each

For peer review only

participant. Findings will be disseminated through peer-reviewed journals, conference presentations and to caregivers at face-to-face meetings.

Strengths and limitations of this study

Strengths:

- The primary strength of this study is the rigorous collection of longitudinal data on morbidity, mortality and nutritional status during inpatient care and for 48 weeks after initial admission for SAM.
- Laboratory sub-studies investigating enteropathy, microbiota, metabolomics and immune cell function provide a unique opportunity to understand which pathogenic pathways contribute to SAM and whether these processes normalise with nutritional rehabilitation, capitalising on a well-characterised cohort with appropriate controls.
- This study builds on existing studies of SAM and HIV-SAM prior to the availability of antiretroviral therapy (ART) and will provide the first assessment of longitudinal clinical outcomes in the current ART era.

Potential limitations:

- High loss to follow-up due to participants returning to home settings following hospital discharge. A dedicated clinical study team is in place to maximise follow-up through phone reminders and community visits.
- The clinical heterogeneity of the study participants, including comorbidities such as stunting and co-infections, may make it challenging to identify the specific causes of clinical outcomes. However, the embedded sub-studies will enable multiple pathways to be explored within the same cohort.

For peer review only

INTRODUCTION

Malnutrition underlies almost half of all childhood deaths in developing countries[1]. Severe acute malnutrition (SAM) is defined by a weight-for-height Z score <-3, mid-upper arm circumference <115mm and/or bilateral pitting oedema[2]. Current treatment guidelines distinguish two groups: i) children with uncomplicated SAM who can be managed in the community; and ii) children with complicated SAM, who are hospitalised and undergo resuscitation, stabilisation and nutritional rehabilitation. In-hospital mortality in children with complicated SAM remains high despite the implementation of WHO guidelines[3]. Furthermore, SAM presents as two major clinical phenotypes: non-oedematous SAM (marasmus), characterised by severe wasting, and oedematous SAM (kwashiorkor), a more complex syndrome characterised by bilateral pitting oedema, steatosis and diarrhea[4 5]. Despite differing clinical outcomes, treatment protocols are the same for both oedematous and non-oedematous SAM.

A contributory factor to high in-patient mortality is the co-occurrence of HIV infection in around one-third of children hospitalised for SAM in sub-Saharan Africa[6 7]. While new HIV infections in children have declined[8], a substantial number of infected children are diagnosed late and present with malnutrition. There is also a growing population of HIV-exposed uninfected (HEU) children who have immune abnormalities, poor growth and higher risk of mortality and infectious morbidity[9]. Hence, HIV has transformed the epidemiology and outcomes of SAM[10]. Even with standardised treatment approaches, inpatient deaths are almost four-fold higher among HIV-infected children with SAM (HIV-SAM), compared to HIV-uninfected children with SAM (30.4% vs 8.4%), for reasons that remain unclear[11]; this mortality is three-fold higher than would be expected from anthropometric parameters alone[11]. Management of HIV-SAM is particularly challenging because HIV fundamentally alters the clinical presentation of malnutrition and the response to treatment. Children

with HIV-SAM are more stunted and wasted; have a higher frequency of persistent diarrhoea; tend to have delayed nutritional recovery and have a more complicated clinical course than children with SAM[11].

Long-term outcomes of SAM

Following resolution of SAM complications and return of appetite, children are discharged from hospital to continue therapeutic feeds at home. However, emerging data indicate high post-discharge mortality following in-hospital management of SAM[12-14]. Malnutrition together with young age, HIV infection and pneumonia have been associated with higher post-discharge mortality[15]. One of the largest prospective studies of growth and mortality in children with SAM (FuSAM), conducted in Malawi from July 2006 to March 2007, collected 12-month outcome data on 87% of 1024 children admitted to the nutrition ward[12]. A total of 427 (42%) died and 44% of these deaths occurred after discharge from hospital. Survival was greatest among those who were nutritionally cured upon discharge from outpatient therapeutic feeding centres, defined as two consecutive visits with >80% expected weight-for-height, no oedema and clinically stable. The risk of mortality after hospital discharge was four-fold higher for HIV-infected compared to HIV-uninfected children, but the outcomes among HEU children were not reported. The loss to follow-up was high in the FuSAM study because there was only one follow-up visit, one year after discharge from outpatient-feeding centres.

The impact of SAM appears to persist beyond the first year after discharge from hospital. The ChroSAM study, which followed children with SAM seven years post-discharge, showed that children had poorer growth, body composition and physical function compared to siblings and community controls, which are all indicators of future cardiovascular and metabolic disease[13].

For peer review only

While anthropometry is used to assess nutritional recovery following discharge from hospital, the pattern and quality of growth recovery in SAM and HIV-SAM are poorly understood. The observation that children treated for SAM have a deficit in lean tissue despite regaining weight suggests that assessing body composition in addition to anthropometry may help to identify children who have not completely recovered and are at potential risk of long-term metabolic diseases[13]. Children with HIV-SAM appear to have potential for catch-up growth since weight-for-age and/or weight-for-height have been shown to normalise with treatment even prior to widespread availability of ART[16]. However, the body composition of children with HIV-SAM compared to SAM has not been described. Whether children with SAM (and in particular HIV-SAM) recover fat mass at the expense of lean mass is unknown, but differences in tissue accretion patterns may have implications for survival and long-term metabolic health[17 18]. There is also a need to consider the effect of SAM on the size of body parts which grow at different rates: relatively shorter legs, for example, are associated with epidemiologic risk of overweight, coronary artery disease, liver dysfunction and diabetes[19 20].

Taken together, there is clearly an elevated risk of mortality among children with HIV-SAM compared to SAM, and an ongoing mortality risk among all children with SAM that persists after discharge from hospital. There are several gaps in our understanding of the long-term outcomes of SAM and HIV-SAM: (i) causes of death have not been clearly defined; (ii) no studies have systematically and longitudinally collected morbidity and mortality data or documented repeat hospitalisations post-discharge; and, (iii) the long-term outcomes of HIV-infected children with SAM in the era of ART availability are unclear.

Pathogenesis of SAM and HIV-SAM

Better understanding the pathogenesis of SAM may help to explain the high mortality

For peer review only

of children both during and after hospitalisation and identify new targets for interventions to supplement existing treatment strategies. Consistent evidence that immune mediators are altered in malnutrition[21] and that systemic and intestinal inflammation are associated with poor outcomes in SAM[22], suggest that immune dysfunction contributes to infectious susceptibility[23]. Malnutrition is also characterised by a complex derangement in gut microbial[24] metabolic,[25] immune[26] and hormonal pathways, organ dysfunction and micronutrient deficiencies in the context of co-infections, enteropathy and chronic inflammation. Several studies have recently provided insights into these perturbations using new tools[25 27-29], including metabolomics and metagenomics, but we still lack a clear understanding of many of the pathogenic pathways driving malnutrition, the interactions between these pathways, and which are the most tractable targets for intervention.

SAM shares several pathological and clinical features with HIV, which may explain clinical outcomes in these co-occuring conditions: 1) both are characterised by intestinal damage, leading to impairment of the mucosal barrier and increased intestinal permeability; 2) both have underlying systemic immune activation; and 3) both are frequently complicated by persistent diarrhoea, pneumonia and sepsis that may plausibly arise due to loss of intestinal barrier function[30]. Understanding the overlapping impact of HIV and SAM is critical to inform additional interventions to improve outcomes of children with HIV-SAM.

OBJECTIVES OF HOPE-SAM

The Health Outcomes, Pathogenesis and Epidemiology of Severe Acute Malnutrition (HOPE-SAM) study has two primary objectives:

1) To describe the short- and long-term clinical outcomes of children with complicated SAM and HIV-SAM, and to identify the risk factors at admission and discharge from hospital that independently predict these outcomes.

For peer review only - http://bmjopen.bmj.com/site/about/guidelines.xhtml

2) To better characterise the pathogenesis of SAM and HIV-SAM, through nested laboratory sub-studies evaluating enteropathy, gut microbiota, metabolomics and immune cell function.

STUDY DESIGN

HOPE-SAM is a longitudinal observational cohort study, enrolling between 600-800 children aged 0-59 months admitted with SAM to the tertiary pediatric wards at two sites in Zimbabwe (Parirenyatwa Hospital and Harare Children's Hospital) and one in Zambia (University Teaching Hospital, Lusaka). Both HIV-infected and HIV-uninfected children will be enrolled. All participants with SAM are followed for 48 weeks post-discharge, with longitudinal data collection and blood sampling; a subgroup of 200 children will be recruited to the enteropathy substudy for which they will have the same follow-up procedures but more intensive biological specimen collection including stool (all time-points), urine after lactulose-mannitol (LM) challenge as an assessment of intestinal permeability, and nasogastric aspirate (baseline only). A group of 200 healthy children recruited from the same hospitals, who are well-nourished and matched to children in the enteropathy substudy by age and HIV status, will have data and specimens collected to provide normative data for the laboratory substudies; these healthy controls will not be followed longitudinally. The study overview is shown in **Figure 1**.

The study protocol, data collection forms and standard operating procedures are available as Supplementary Materials.

RECRUITMENT

Screening: Caregivers of all hospitalised children are sensitised about the study. All new admissions aged 0-59 months are screened for SAM, which is defined

For peer review only

according to WHO criteria as any of: weight-for-height Z-score (WHZ) <-3, mid-upper arm circumference <115 mm (if aged 6-59mo) and/or bilateral pitting oedema.

Eligibility for observational cohort: All children with SAM whose caregivers are willing to provide written informed consent and to learn their child's HIV status are offered enrolment. Any children who die prior to study enrolment and those with a known malignancy are ineligible.

Eligibility for enteropathy substudy: Cases (Groups A and C, **Table 1**) are children with SAM aged 6-59 months with a nasogastric tube in place (or due to be placed). Controls (Groups B and D) are children receiving inpatient or outpatient care at the study sites, who are aged 6-59 months (matched to cases within age bands), well-nourished (weight-for-height Z-score >-1) and clinically well (no acute illness or current infections) with known HIV status. Children with underlying chronic gastrointestinal disease or a known malignancy are ineligible.

For peer review only

Table 1: Enteropathy substudy groups

**Children aged 6-59 months**	**Severe acute malnutrition[1]**		**Well nourished** WHZ>-1
	**Oedematous[2]**	**Non-oedematous**	
**HIV-infected** (HIV PCR+ if <18mo; HIV antibody + if >18mo)	N=50 (Group A-I)	N=50 (Group A-II)	N=100[3] (Group B)
**HIV-uninfected** (HIV PCR- if <18mo; HIV antibody - if >18mo)	N=50 (Group C-I)	N=50 (Group C-II)	N=100[4] (Group D)

[1]SAM defined according to WHO criteria
[2]Presence of bilateral pitting pedal oedema.
[3]Age-matched to group A (within the following age bands: 6-11 months; 12-23 months; 24-59 months).
[4]Age-matched to group C (within the following age bands: 6-11 months; 12-23 months; 24-59 months)
Note that children age 0-5 months are excluded from the enteropathy substudy

WHZ: Weight-for-height Z score; MUAC: Mid-upper arm circumference; PCR: polymerase chain reaction.

Eligibility for other sub-studies: Children enrolled into the enteropathy substudy are also included in the microbiome and metabolome substudies. The immunology substudy comprises all children with SAM providing a blood sample of sufficient volume (>2ml) for cellular assays.

Informed consent procedures: Written informed consent is obtained from the primary caregiver using consent forms translated into local languages; we generally include other family members in the consent process. Illiterate caregivers who have understood a verbal explanation of the study can provide a thumb imprint in the presence of a witness. Assent from children is not sought because all are <5 years old.

STUDY PROCEDURES

Study procedures are outlined in **Table 2**.

Table 2: Summary of procedures in observational SAM cohort

**Assessment (Form used)**	**Hospitalization**		**Post-discharge[3]**				
	**Baseline[1]**	**Discharge[2]**	**2w**	**4w**	**12w**	**24w**	**48w**
Caregiver informed consent to join observational cohort **(HOPE-SAM Form_2_Observational Cohort Informed Consent Form** and **HOPE-SAM Form_6_Specimen Storage and Shipment form**)	x						
Summary checklist (**HOPE-SAM Form_3_Study Checklist**)	x						
Locator information[4] (**HOPE-SAM Form_9_Locator Information**)	x						
Acute admission information **(HOPE-SAM**	x						

**Form_4_Acute Admission Proforma)**							
Baseline data (**HOPE-SAM Form_5_Baseline Form)**	x						
Daily clinical review[5] (**HOPE-SAM Form_7_Daily Follow-up Form**)	Daily during hospitalisation						
Blood collection[6]	x	x			x	x	x
HIV testing[7]	x						
CD4 count and viral load (HIV-infected children only)	x				x	x	x
Full blood count[8]	x	x			x	x	x
Anthropometry	x	x	x	x	x	x	x
Skinfold thickness[9]		x	x	x	x	x	x
Body composition[10]	x	x	x	x	x	x	x
Discharge data collection (**HOPE-SAM Form_8_Discharge Form**)		x					
Daily morbidity diary (**HOPE-SAM Form_10_Morbidity Diary**)			Daily during follow-up period by caregivers				
Follow-up clinic: history, examination, morbidity and mortality data (**HOPE-SAM Form_11_Clinical Follow-up Form** and **HOPE-SAM Form_12_Nurse Follow-up Form)**			x	x	x	x	x

[1]Children will be enrolled as soon as possible after hospitalisation and will undergo baseline investigations as soon as possible after enrolment. This is to provide a window of opportunity to time collection of research specimens with clinical specimens, and to ensure that the child is clinically stable before undertaking research investigations.
[2]The discharge procedures will be undertaken on the day of discharge, or as close as possible to that date.
[3]Windows will be created around these post-discharge time-points to maximize follow-up for caregivers who miss visits or are unavailable, as follows: 2 weeks (1-3 weeks); 4 weeks (3-5 weeks); 12 weeks (10-14 weeks); 24 weeks (20-28 weeks); 48 weeks (44-52 weeks).
[4]Locator information will updated using **HOPE-SAM Form_9_Locator Information** at subsequent visits if caregivers have moved or changed contact details.
[5]A clinical review will be undertaken every day between admission and discharge by the study clinician, using **HOPE-SAM Form_7_Daily Follow-up Form.**
[6]5.4 mL of blood (depending on child weight; amount will not exceed 2 mL/kg total over 2 week period) will be collected by a study nurse into endotoxin-free EDTA tubes. Samples will be used to store whole blood, PBMC and plasma for subsequent measurement of CRP and albumin. Where blood sample volumes allow (≥2mL sample), bacterial binding assays and whole blood stimulations will be conducted and culture supernatants and cells stored for subsequent assessment of immune cell function at each time-point.
Study blood samples will not be collected from children with known haemoglobin <6 g/dL.

For peer review only

[7]Infant HIV testing is conducted as part of routine clinical practice, but if it has not been undertaken, the study sample will be used to test for HIV, as stated in the informed consent form, since HIV status is required to allocate children to study groups.
[8]Full blood count results will be transcribed from clinical records; if not done by clinical teams, the EDTA sample will be used to measure FBC in clinical laboratories at each site
[9]Skinfold thickness (triceps, subscapular, supra-iliac) and mid-thigh circumference will be measured using Holtain calipers or tape measure.
[10]Body composition will be assessed by bioimpedance vector analysis.

Baseline procedures:

Baseline data on maternal and household characteristics, the child's past medical history and current illness are collected by a study nurse. Anthropometry, including body composition measured by whole-body (wrist-ankle) bio-electrical impedance analysis (BodyStat 1500MD; BodyStat Ltd., Douglas, Isle of Man), leg length using an electronic knemometer (Zimbabwe only) and triceps, subscapular and supra-iliac skinfold thickness using calipers (Holtain Ltd., Crymych, UK) are undertaken at baseline. Blood (1mL/kg up to 5.4mL maximum) is collected at baseline into an endotoxin-free EDTA tube for all children and, in the enteropathy substudy, additionally into a PAXgene tube (PreAnalytiX GmbH, Hombrechtikon, Switzerland) for subsequent transcriptomic analysis. Blood is not collected from children with severe anaemia (known haemoglobin <6 g/dL). HIV testing is carried out in accordance with national guidelines as part of routine clinical practice; where it has not been done, the child's HIV status is ascertained using a rapid test antibody algorithm for children over 18 months, or HIV DNA PCR for children under 18 months. CD4 count/percentage and viral load are measured in HIV-infected children. Maternal HIV status is documented where available, so that HIV-exposed uninfected children can be identified. Blood samples are sent to research laboratories at each site to conduct whole blood stimulation and bacterial binding assays (as described in the immunology substudy) and to store aliquots of whole blood, peripheral blood cells and plasma at -80ºC[31]. In the enteropathy substudy, nasogastric aspirate, stool and urine (after an oral dose of lactulose and mannitol) are also collected. Lactulose and

For peer review only - http://bmjopen.bmj.com/site/about/guidelines.xhtml

mannitol are ingested by the child after fasting and urine is collected over a two-hour period to measure recovery of lactulose and mannitol, a measure of intestinal absorptive capacity and permeability, as previously described[32].

Daily procedures: Routine inpatient management is undertaken by ward clinical teams according to local hospital protocols, which are based on WHO guidelines[33]. In addition, the HOPE-SAM study clinician at each hospital site collects daily data until discharge on clinical parameters (including daily examination), resolution of acute infections, nutritional recovery (loss of oedema, restoration of appetite, weight gain), and treatment/nutritional supplements received. Children with HIV-SAM who are ART-naïve start ART according to WHO guidelines[34].

Discharge: The clinical team decides when the child is ready to be discharged. Children receive ready-to-use therapeutic feeds (RUTF) to take at home according to local guidelines. At discharge, the study nurse collects data and a repeat blood sample (including full blood count) and undertakes discharge anthropometry, body composition, leg length (Zimbabwe only) and triceps, subscapular and supra-iliac skinfold thickness measurements (**Table 2**). The caregiver is given a daily morbidity diary and pre-prepared stickers corresponding to different illnesses and shown how to complete the diary. The caregiver is provided with the date of the first follow-up appointment and contact details of the study nurse.

Follow-up: Children attend follow-up appointments at dedicated study clinics at 2, 4, 12, 24 and 48 weeks post-discharge. At each visit, the study physician undertakes a clinical assessment and the study nurse captures illness, medication and feeding data. Clinic data are transcribed from handheld medical records if available and the morbidity diary is reviewed and a new diary and stickers supplied. Anthropometry, body composition, leg length (Zimbabwe only) and triceps, subscapular and supra-

For peer review only - http://bmjopen.bmj.com/site/about/guidelines.xhtml

iliac skinfold thicknesses are measured at each visit. Acute illnesses are treated in the study clinic, or the child is referred to hospital if necessary. Children with relapsed malnutrition are provided with nutritional supplements according to local guidelines. Transport reimbursement for clinic attendance is provided to caregivers for each visit.

Blood is collected at weeks 12, 24 and 48 post-discharge into endotoxin-free EDTA tubes to measure full blood count, CD4 count and viral load (HIV-infected group only), conduct whole blood stimulation and bacterial binding assays, and store peripheral blood cells and plasma aliquots for subsequent analysis, including soluble and cellular markers of immune activation, as outlined in **Supplementary Table 1**. Children in the enteropathy substudy have additional stool and urine collection following lactulose-mannitol dosing as shown in **Table 3.**

For peer review

Table 3: Summary of procedures for cases in the enteropathy substudy

**Assessment (Form used)**	**Hospitalization**		**Post-discharge[3]**				
	**Baseline[1]**	**Discharge[2]**	**2w**	**4w**	**12w**	**24w**	**48w**
Caregiver informed consent to join observational cohort and enteropathy substudy **(HOPE-SAM Form_14_Enteropathy Substudy Case Consent Form** and **Form_6_Specimen Storage and Shipment form**)	x						
Summary checklist (**HOPE-SAM Form_3_Study Checklist**)	x						
Locator information[4] (**HOPE-SAM Form_9_Locator Information**)	x						
Acute admission information **(HOPE-SAM Form_4_Acute**	x						

**Admission Proforma)**							
Baseline data (**HOPE-SAM Form_5_Baseline Form)**	x						
Daily clinical review[5] (**HOPE-SAM Form_7_Daily Follow-up Form**)	Daily during hospitalisation						
Blood collection[6]	x	x			x	x	x
HIV testing[7]	x						
CD4 count and viral load (HIV-infected children only)	x				x	x	x
Full blood count[8]	x	x			x	x	x
Gastric aspirate[9]	x						
Stool collection[10]	x	x			x	x	x
Lactulose-mannitol testing[11]	x	x			x		x
Anthropometry	x	x	x	x	x	x	x
Skinfold thickness[12]		x	x	x	x	x	x
Body composition[13]	x	x	x	x	x	x	x
Discharge data collection (**HOPE-SAM Form_8_Discharge Form**)		x					
Daily morbidity diary (**HOPE-SAM Form_10_Morbidity Diary**)			Daily during follow-up period by caregivers				
Follow-up clinic: history, examination, morbidity and mortality data (**HOPE-SAM Form_11_Clinical Follow-up Form** and **HOPE-SAM Form_12_Nurse Follow-up Form)**			x	x	x	x	x

[1]Children will be enrolled within 24h of hospitalization and will undergo baseline investigations within 72h of hospitalization. This is to provide a window of opportunity to time collection of research specimens with clinical specimens, and to ensure that the child is clinically stable before undertaking research investigations.
[2]The discharge procedures will be undertaken on the day of discharge, or as close as possible to that date.
[3]Windows will be created around these post-discharge time-points to maximize follow-up for caregivers who miss visits or are unavailable, as follows: 2 weeks (1-3 weeks); 4 weeks (3-5 weeks); 12 weeks (10-14 weeks); 24 weeks (20-28 weeks); 48 weeks (44-52 weeks).
[4]Locator information will updated using **HOPE-SAM Form_9_Locator Information** at subsequent visits if caregivers have moved or changed contact details.

[5]Daily clinical review will be conducted every day between admission and discharge by the study clinician, using **HOPE-SAM Form_7_Daily Follow-up Form**
[6]During hospitalisation, 5.4 mL of blood (depending on child weight; amount will not exceed 2 mL/kg total over 2 week period) will be collected by a study nurse into a 2.7 mL endotoxin-free EDTA tube and a 2.7 mL PAXGene tube, for subsequent isolation of RNA and gene expression analysis (see Table 7). After discharge (weeks 12, 24 and 48), 5.4 mL of blood (depending on child weight; amount will not exceed 2 mL/kg total over 2 week period) will be collected by a study nurse into two 2.7 mL endotoxin-free EDTA tubes.
[7]Infant HIV testing is conducted as part of routine clinical practice, but if it has not been undertaken, the study sample will be used to test for HIV (see section 9.4), as stated in the informed consent form, since HIV status is required to allocate children to study groups.
[8]Full blood count results will be transcribed from clinical records; if not done by clinical teams, the EDTA sample will be used to measure FBC in clinical laboratories at each site
[9]A gastric juice sample will be collected at the same time as the blood draw by aspirating the nasogastric tube with a sterile feeding syringe, to test for gastric pH; sterile water or saline will then be instilled and a sample of gastric juice collected for storage for subsequent PCR and culture (section 7.5.2)
[10]Stool collection will be undertaken at the same time as the blood draw as described in section 7.5.3.
[11]Lactulose-mannitol testing will be conducted as described in section 7.5.4, with collection of a baseline urine sample, followed by a 2hr urine collection post-LM ingestion. This test will be deferred until children are judged to be clinically stable by the study physician during daily reviews. In general, this will be a child in the nutritional rehabilitation phase, who has no cardiorespiratory compromise.
[12]Skinfold thickness (triceps, subscapular, supra-iliac) and mid-thigh circumference will be measured using Holtain calipers or tape measure as explained in section 7.5.7.
[13]Body composition will be assessed by bioimpedance vector analysis (see section 7.5.6)

Caregivers are reminded of follow-up visits by phone, and visit completion is tracked on a dedicated database. If caregivers do not attend follow-up appointments, attempts are made to contact them by phone and home visits are made if feasible, particularly for those defaulting the 48-week visit, so that long-term outcome data can be collected. For post-discharge deaths, a home visit is undertaken by study nurses where possible to conduct a verbal autopsy. Children who are readmitted to one of the study sites with relapsed SAM have data collected during the new episode of hospitalisation. The study ends for each participant at the week 48 visit.

SUBSTUDIES

Several nested substudies will utilise biological specimens to address mechanistic questions related to enteropathy, microbiota, metabolomics and immune function.

For peer review only

Enteropathy substudy

The gut, which acts as an internal interface between humans and the environment, must contain the nutrient stream and the symbiotic microbiota while allowing molecular intimacy to permit absorption. The mechanism underlying this duality is the integrity of the gastrointestinal barrier; intestinal damage (enteropathy) can impair this critical barrier function. A spectrum of enteropathies affect children in developing countries[30]. Environmental enteric dysfunction (EED), characterised by small intestinal inflammation, blunted villi and increased intestinal permeability, is almost universal and is morphologically indistinguishable from HIV enteropathy[30]. Children in resource-poor settings also suffer from frequent diarrhoea, food insecurity and micronutrient deficiencies, which all exacerbate enteropathy[30]. As a result, a cycle of intestinal infection, impaired mucosal function and malnutrition commonly arises, which may ultimately precipitate SAM, especially in the context of HIV infection[35 36]. It is not yet established if the enteropathy seen in children with SAM[37], which we here refer to as malnutrition enteropathy[37], is qualitatively or quantitatively distinguishable from EED. In addition to local intestinal pathology, enteropathies may cause systemic pathology due to persistent immune activation arising from enteric inflammation and microbial translocation across the damaged gut wall[30]. It is becoming apparent that chronic inflammation may be particularly deleterious in malnourished individuals[22]; in children with SAM, systemic inflammation arising from underlying enteropathy may further increase morbidity and mortality.

We hypothesize that i) the degree of enteropathy during hospitalisation differs between oedematous and non-oedematous SAM and is independently associated with morbidity, mortality and nutritional recovery during hospitalization; ii) the degree of enteropathy at discharge is independently associated with morbidity, mortality and relapse of SAM; and iii) children with HIV-SAM have more severe enteropathy than children with SAM alone, which contributes to their poorer outcomes.

For peer review only

Using stored samples, a longitudinal series of investigations will compare gastric and small intestinal barrier function, using a range of biomarkers to capture the domains of malnutrition enteropathy (**Supplementary Table 2**). To understand better the extra-intestinal consequences of enteropathy, we will first compare the microbial composition of the upper gut and plasma using deep sequencing in a subgroup of children with paired gastric and blood samples. Secondly, we will undertake transcriptomics using PAXGene blood samples to determine i) whether there are differences in gene expression profiles between healthy controls, SAM and HIV-SAM (including comparison of oedematous and non-oedematous types); and ii) whether specific patterns of gene expression are associated with morbidity and mortality in SAM.

Microbiota substudy

Normal assembly of the gut microbiota in early life is critical for many aspects of physiological, neurological and immune development[38]. Recent evidence suggests that an immature or pathogenic microbiota plays a causative role in the pathogenesis of SAM[27]. For example, a number of microbial taxa have been identified, including *Faecalibacteium prausnitzii*, which discriminate and predict gut microbiota maturity and child growth[39]. Other pathogenic microorganisms, including IgA-targeted *Enterobacteriaceae*, are associated with impaired growth and may contribute to SAM[40]. Nutritional rehabilitation with RUTF induces temporary recovery of a disturbed microbiota; however, the microbiota appears to revert back to an immature diseased state following nutritional recovery[41]. HIV infection is also associated with a disturbed gut microbiota[42], which may further compound enteropathy phenotypes. Furthermore, there is some evidence that differences exist in malnutrition enteropathy between oedematous and non-oedematous SAM[43]; however, few

For peer review only - http://bmjopen.bmj.com/site/about/guidelines.xhtml

studies have investigated differences in the gut microbiota between the two forms of the disease.

We hypothesize that (i) a unique gut microbial signature exists in HIV-SAM, compared with SAM, that is independently associated with morbidity, mortality, nutritional recovery and degree of enteropathy during hospitalisation; (ii) a unique gut microbial signature exists in oedematous compared with non-oedematous SAM; (iii) specific microorganisms or gut microbial diversity indices are independently associated with morbidity, mortality, nutritional recovery and degree of enteropathy during hospitalisation; and (iv) the gut microbiota is partially restored to a healthy state with nutritional rehabilitation but reverts to a dysbiotic state during follow-up, which predicts morbidity, mortality and relapse of SAM.

Using stored stool samples collected at baseline, a cross-sectional investigation will determine differences in the gut microbial composition and predicted function between SAM versus HIV-SAM, oedematous versus non-oedematous SAM, and healthy controls. Gut microbial composition and predicted function will be compared between groups at discharge and at 12, 24 and 48 weeks post-discharge. Briefly, total DNA and/or RNA will be extracted from stool samples and used as template for next-generation sequencing library preparation and for quantitative polymerase chain reaction (qPCR). Whole metagenome shotgun sequencing will be performed using the HiSeq 2500 system. Raw metagenomic sequencing data will be quality-filtered and analysed through a well-validated bioinformatics pipeline using MetaPhlAn[44] and HUMAnN[45]. The compositional and predicted functional metagenomic data generated will be used to identify signatures of SAM and to investigate associative links between specific gut microbial signatures and clinical outcomes.

For peer review only - http://bmjopen.bmj.com/site/about/guidelines.xhtml

Metabolomics substudy

During SAM, metabolic processes are altered in response to a starved environment, and may plausibly contribute to long-term clinical outcomes. Previous studies suggest that amino acid turnover, lipid metabolism, oxidative stress and other metabolic pathways are disrupted in SAM and may be associated with disease state and clinical outcome[25 46 47]; however, little is known about how the metabolic phenotype responds to nutritional therapy. It is hypothesised that disturbed gut microbiota composition and function may drive microbial metabolic dysregulation in addition to host-derived dysregulation. Of particular interest are differences in the metabolic phenotype between oedematous and non-oedematous SAM. The 'reductive adaptation' seen in non-oedematous SAM (utilisation of fat and muscle stores) is disrupted in oedematous SAM, which may contribute to differences in clinical outcomes. Specifically, protein turnover, inflammation, oxidative stress and bile acid metabolism are disrupted in oedematous-SAM, which may contribute to co-morbidities including diarrhoea, steatosis and enteropathy[48 49].

We hypothesize that: (i) a unique plasma and urine metabolic phenotype exists in oedematous compared with non-oedematous SAM during hospitalisation, which is independently associated with morbidity, mortality and nutritional recovery; (ii) the metabolic phenotype is partially restored to a healthy state with nutritional rehabilitation but reverts to a disturbed state during follow-up, which predicts morbidity, mortality and relapse; and (iii) both host-derived and gut microbial-driven metabolic dysregulation underlie clinical outcomes.

Using stored urine and plasma samples collected during hospitalisation, a cross-sectional investigation will determine differences in the metabolic phenotype between children with oedematous SAM, non-oedematous SAM and healthy controls. Urine and plasma metabolic phenotypes will be compared between groups at discharge

For peer review only - http://bmjopen.bmj.com/site/about/guidelines.xhtml

and 12, 24 and 48 weeks post-discharge. Briefly, global untargeted metabolomic phenotyping will be performed via ^{1}H nuclear magnetic resonance (NMR) spectroscopy using a 700 MHz Bruker NMR spectrometer to identify metabolic signatures of SAM. Targeted analysis via ultra-performance liquid chromatography-mass spectrometry will be performed to examine specific pathways of interest, including tryptophan and bile acid metabolism.

Immunology substudy

Bacterial infections are common among children hospitalised for SAM[50-53] and mortality is driven by a range of species[51 53-56], consistent with generalised defects in innate anti-bacterial defence. Increased infectious morbidity and mortality persist after discharge from hospital[57-59], suggesting that restoration of anti-bacterial immune responses may lag behind nutritional rehabilitation. A recent randomised trial in children with SAM confirmed that deaths following hospitalisation were predominantly due to bacterial infections but were not prevented by daily co-trimoxazole prophylaxis[59]. Collectively, these observations highlight that children remain vulnerable to infection despite current treatment approaches; targeting persistent immune dysfunction could plausibly reduce infectious mortality after discharge[60].

Multiple innate and adaptive immune mediators are dysregulated in malnutrition[60-62]. However, few studies have assessed cellular immune function in malnourished children; most existing studies were undertaken decades ago on small cross-sectional cohorts without the benefit of recent advances in immunology techniques[61]. Immune dysfunction in SAM likely reflects both *intrinsic* defects, whereby immune cells lack capacity to adequately respond to infection, and *extrinsic* defects, where cells have intact anti-bacterial capacity but are chronically modulated by the systemic pro-inflammatory environment which characterises SAM (i.e. heightened pro-inflammatory cytokines[50 63] and circulating bacterial antigens[64 65]). Systemic

For peer review only - http://bmjopen.bmj.com/site/about/guidelines.xhtml

inflammation is directly associated with mortality in SAM[50] and driven by multiple comorbidities, including bacterial translocation from the damaged gut into the blood, sub-clinical infections and metabolic dysregulation[63 66-68]. The implications of innate immune cell dysfunction for subsequent acquisition of infections and infectious mortality have not been investigated.

We hypothesise that: (i) anti-bacterial functions of innate immune cells are compromised in SAM due to a combination of intrinsic and extrinsic defects; ii) innate immune cell function is independently associated with infectious morbidity and mortality during hospitalisation for SAM; and iii) nutritional rehabilitation only partly restores innate immune cell function, leading to an ongoing risk of bacterial infections post-discharge.

Using blood samples collected at baseline, discharge and 12, 24 and 48 weeks post-discharge, the longitudinal relationship between circulating innate immune cell function and bacterial infections will be assessed. The intrinsic phagocytic capacity, secreted cytokine response and maturation state of innate immune cells after culture with bacterial antigens will be assessed. Plasma concentrations of endotoxin and pro-inflammatory cytokines will be quantified at each time-point and the degree to which these extrinsic factors influence innate immune cell antibacterial function will be assessed via plasma co-culture with innate immune cells from healthy donors. Bacterial infections during hospitalisation will be diagnosed using clinical criteria and blood culture, stool culture and urinalysis where available.

SAMPLE SIZE

Observational study: The observational cohort will recruit as many children with SAM as possible during the period of enrolment (July 2016 to March 2018), estimated at 600-800 children, to provide a robust assessment of outcomes among children

For peer review only - http://bmjopen.bmj.com/site/about/guidelines.xhtml

hospitalised with SAM. Assuming 15% mortality and 15% loss to follow-up, there would be at least 420 evaluable children post-discharge, of whom up to 168 would have HIV-SAM, based on an estimated inpatient HIV prevalence of 40%.

Enteropathy substudy: The sample size was estimated using previously reported values for LM ratios, which remain a widely used non-invasive marker of enteropathy. Comparing 100 versus 100 children with two-sided alpha=0.025 (to allow for two primary comparisons, i.e. HIV-SAM versus SAM, and HIV-SAM versus HIV) provides >80% power to detect differences in mean LM ratio during hospitalisation of at least 0.16 (assuming SD=0.36), a difference which would be clinically relevant given the LM ratios previously reported for well-nourished children (0.42), malnourished children (1.3) and children with persistent diarrhoea (2.85) in the Gambia[69]. It also provides >80% power to detect differences of at least 0.1 in the mean change in LM ratio from enrolment (assuming SD for change=0.23 and 7% missing samples). For inflammatory markers, comparing 100 versus 100 children with two-sided alpha=0.025 provides >80% power to detect differences in mean $\log_{10}$ concentrations of at least 0.44 times their standard deviation, or 2.75-fold differences between groups. Inclusion of healthy controls provides an indication of normal ranges in young African children. SAM groups will be stratified to include approximately 50 children with and without oedematous malnutrition, if possible.

Microbiota and metabolomics substudy

Power calculations are difficult in metagenomics and metabolomic analyses due to the large number of observed outcomes and unknown effect sizes and variance. Previous studies using smaller sample sizes have identified significant taxonomic differences in twin pairs discordant for oedematous-SAM (n=13)[24] and metabolic differences between the two forms of SAM (n=40)[70]. These studies suggest that a difference of 50% in metabolites could be expected. Using ANCOVA, setting α=0.05

For peer review only - http://bmjopen.bmj.com/site/about/guidelines.xhtml

and assuming either low (ρ=0.1) or high (ρ=0.7) correlation, the study would require 95-126 subjects to achieve 80% power[71]. False discovery rate (FDR) multiple correction testing will be applied to reduce the high-dimensionality of the data and limit false-positives.

Immunology substudy

Up to 200 children with SAM and 200 healthy controls will be included in a cross-sectional analysis of innate immune cell function during hospitalisation. Assuming similar infectious mortality to a recent Kenyan study (15%)[59], a cohort of 200 provides 80% power to detect associations between immune profiles and infectious mortality at an odds ratio of 1.7 and 2-sided alpha of 0.05. We will aim for 100 children with longitudinal analysis of innate immune cell function at discharge, 12, 24 and 48 weeks post-discharge[59].

ANALYSIS

Observational Cohort

The primary comparison will be the clinical and nutritional outcomes of children with SAM compared to HIV-SAM. We will review all deaths and adjudicate clinical diagnoses and causes of death to ensure robust and consistent data across sites. We will compare each participant's clinical management to WHO guidelines to identify any contributory factors in hospital care. Factors associated with outcomes during hospitalisation (e.g. mortality, nutritional recovery) will be determined for each group using multivariate analysis (Cox models for time-to-event data, linear models for continuous outcomes). Factors associated with outcomes over 48 weeks post-discharge (hospital re-admission, morbidity and mortality, relapse, anthropometry, body composition and response to ART) will be determined for each group using multivariate analysis (Cox models for time-to-event data, linear models for continuous outcomes). We will evaluate the ability of MUAC at discharge to predict

For peer review only - http://bmjopen.bmj.com/site/about/guidelines.xhtml

long-term outcomes using receiver-operator-characteristic (ROC) analysis, in the whole cohort and within the HIV-SAM and SAM subgroups. We will then evaluate whether addition of other variables improves the predictive capacity of MUAC for each group, including body composition, haemoglobin, albumin and CRP, plus CD4%, viral load and timing of ART initiation (HIV-SAM only). We will construct multivariable models and compare them with MUAC alone using the net-reclassification index.

Body composition analysis

Previous work in body composition by bio-electrical impedance in Ethiopian infants and children with SAM has shown that the conventional approach, predicting total body weight from height-adjusted impedance, fails due to confounding by oedema[72]. The same project validated an alternative approach, known as Bio-electrical Impedance Vector Analysis (BIVA), and described significant differences between each of three groups: healthy controls, oedematous-SAM and non-oedematous SAM. Vector analysis splits impedance into two height-adjusted components, resistance and reactance, which are further linked through phase angle (PA). Variability in these components is associated with biochemical parameters[73]. These variables will be explored using graphical analysis, or transformed into age- and sex-adjusted z-scores for statistical comparison, including longitudinal analyses. Higher phase angle indicates better nutritional status, while declining height-adjusted resistance over time indicates loss of oedema.

Enteropathy substudy

The primary comparison for the enteropathy substudy will be between children with HIV-SAM (group A) and SAM (group C), stratified by presence or absence of oedema. Control groups (B and D) are well-nourished children with or without HIV, to provide normative data for biomarkers. For each continuous outcome, simple

For peer review only - http://bmjopen.bmj.com/site/about/guidelines.xhtml

descriptive analysis will be used to compare groups (HIV-SAM versus HIV, and HIV-SAM versus SAM) during hospitalisation using t-tests on appropriately transformed data. For any outcome with moderate ($p<0.05$) evidence of difference between either group a regression model will be constructed including groups A, B, C, D to directly test (using interactions) whether there is a synergistic effect of HIV-SAM versus SAM versus HIV versus neither. These models will also be used to explore whether there is any evidence for heterogeneity in effects between oedematous and non-oedematous SAM. Associations between enrolment factors (e.g. intestinal permeability and microbial translocation) will be explored using pairwise Spearman correlations and principal components analysis. Mean changes at the follow-up time-points in each group will be estimated, and groups compared (as above) using generalised estimating equations. For outcomes that differ across SAM groups over time, multilevel models will be used to explore possible predictors from the other factors measured. Time to nutritional recovery will be compared using Kaplan-Meier and log-rank tests.

Microbiota and metabolomics substudy

The primary comparison will be between HIV-uninfected children with oedematous and non-oedematous SAM, with a separate comparison between children with HIV-SAM and SAM. Analyses will examine: (i) differences in metagenomic/metabolomic variables between groups at each time-point; (ii) differences in metagenomic/metabolomic variables within groups over time; (iii) correlations between metagenomic and metabolomic variables; and (iv) correlations between metagenomic/metabolomic variables and clinical outcomes. A systematic analysis will be undertaken to reduce high-dimensional data, integrate the multi-omics datasets and minimise false discovery.

For peer review only

Compositional metagenomic data will be compared between groups for indices of alpha and beta diversity. Principal coordinate analysis and partial least squares discriminant analysis will be performed on metabolomics data to identify overall differences between groups. High-dimensional datasets will be reduced using random forest models to identify taxa, microbial gene families and metabolites that most strongly contribute to differences between groups, corrected by Benjamani-Hochburg false discovery rate detection. Targeted analysis by qRT-PCR will validate differential abundance or expression of candidate microbial genes. Longitudinal comparisons will be performed within and between groups using multilevel simultaneous component analysis. Orthogonal projections to latent structures models will integrate metabolomic and metagenomic data whilst linear regression, canonical correlation and hierarchal clustering analysis will measure correlations between -omics datasets. Finally, ROC analysis will identify the ability of different analytes to predict long-term nutritional and clinical outcomes.

Immunology substudy

Integrated profiles of innate immune cell function will be generated for each child using principal components analysis followed by hierarchical clustering[74 75]. This data-reduction method identifies whether absolute levels of specific markers or relative differences between markers differentiate children into groups. The resulting innate immune profiles will be compared between SAM, HIV-SAM and well-nourished groups using univariate tests and multivariate analysis of variance (MANOVA) of the principal components.

To address the relationship between immune function and infections, regression analyses will determine whether baseline innate immune profiles (or the individual parameters defining them) are associated with the infectious morbidity or mortality during hospitalisation, using logistic models for binary outcomes and linear models

For peer review only - http://bmjopen.bmj.com/site/about/guidelines.xhtml

for duration. Key clinical characteristics, including age, sex, oedema and baseline WHZ, will be added to models to investigate their confounding effects. Multivariate stacked regression methods will be used to compare the impact of different factors on severe bacterial infections based on heterogeneity tests.

To determine whether treatment for SAM restores innate immune cell antibacterial function, mixed effects regression models will compare longitudinal changes in individual immune parameters, and the principal components calculated from the weights identified at baseline (which include healthy controls). Similarities and differences in longitudinal immune profiles will be compared between groups using nonmetric multi-dimensional scaling[74 76 77]. This approach will group children according to their composite innate immune function, allowing the duration and variability of immune restoration to be evaluated over the course of nutritional rehabilitation. Binary logistic regression will determine whether innate immune profiles at discharge are associated with morbidity or mortality during follow-up.

PATIENT AND PUBLIC INVOLVEMENT

Patients and their caregivers were not involved in the design of the study. During recruitment, all caregivers of children admitted to hospital were given information about the study; those whose children had severe acute malnutrition were approached to give written informed consent. A meeting to disseminate results of the study to participants and their caregivers will be held at the end of the study. An interactive game to engage caregivers in the science underlying malnutrition is being developed in collaboration with experts from the Centre of the Cell, a unique science education centre based at Queen Mary University of London (https://www.centreofthecell.org/).

For peer review only - http://bmjopen.bmj.com/site/about/guidelines.xhtml

SAFETY REPORTING

For all adverse events, the study team will assess expectedness and relatedness to study activities. Since this is an observational study without interventions, we anticipate that the risk is minimal; however, serious adverse events will be reported to local ethical review boards (Medical Research Council of Zimbabwe, and University of Zambia Biomedical Research Ethics Committee) and the study sponsor (Queen Mary University of London) according to their respective guidelines.

DATA COLLECTION AND MONITORING

Clinical and demographic data are recorded on paper case report forms. All data are checked for completeness and plausibility before data entry and problems flagged for resolution by the clinical team. All data are double-entered onto a dedicated password-protected study database, and any discrepancies resolved. Study participants are identified on electronic databases only by study numbers (assigned at enrolment); no personal identifiers are entered.

ETHICS AND DISSEMINATION

The study complies with the principles of the Declaration of Helsinki (2008) and is conducted in compliance with the principles of Good Clinical Practice (GCP) and local regulatory requirements in each country. Ethical approval was obtained from the University of Zambia Biomedical Research Ethics Committee, the Joint Research Ethics Committee of the University of Zimbabwe and the Medical Research Council of Zimbabwe. The ethical review board of the Sponsor, Queen Mary University of London, provided an advisory review of the study. Since this is an observational study, there is no Data and Safety Monitoring Board.

For peer review only

For peer review only - http://bmjopen.bmj.com/site/about/guidelines.xhtml

Results will be disseminated through conference abstracts and peer-reviewed publications and discussed with relevant policymakers and programmers. Study findings will be disseminated to families of participants at face-to-face meetings.

TIME FRAME AND STUDY STATUS

Enrolment into the study began in July 2016 and is expected to end in March 2018. All participants will be followed for 48 weeks, with an expected study completion date of March 2019.

DISCUSSION

HOPE-SAM aims to document the short- and long-term outcomes of children with SAM and HIV-SAM, and to identify the factors at presentation and at discharge from hospital that independently predict these outcomes. Mechanistic substudies aim to evaluate the contribution of enteropathy, microbiota, metabolome and innate immune cell function to these clinical outcomes. The prevalence of malnutrition in HIV-infected children is as high as 40% in some settings and the challenges of managing this population are well recognised[78]. The WHO protocol on management of SAM aims to reduce case fatality below 10%, but rates as high as 35% are still reported among HIV-infected children[79 80]. No studies have systematically and longitudinally collected morbidity data in HIV-SAM, or documented repeat hospitalisations and mortality after discharge from hospital, particularly in the current era where ART is available upon diagnosis. HOPE-SAM will provide a unique opportunity to enrol and follow a cohort of children managed for SAM in three large hospitals across two sub-Saharan African countries at several time-points over a one-year period. Nested longitudinal laboratory substudies aim to better characterise the pathogenesis of SAM and HIV-SAM, to determine whether pathogenic processes are normalised during nutritional rehabilitation and follow-up, and to identify potential mechanistic pathways for new intervention approaches.

For peer review only - http://bmjopen.bmj.com/site/about/guidelines.xhtml

ACKNOWLEDGEMENTS

Members of the HOPE-SAM study team not listed in the author list: *Harare, Zimbabwe:* Virginia Sauramba, Adlight Dandadzi, Chipo Kureva, Johnson Mushonga, Eddington Mpofu, Washington Dune, Tafadzwa Chidhanguro, Sibongile Nkiwane, Sandra Rukobo, Margaret Govha, Patience Mashayanembwa, Leah Chidamba, Bernard Chasekwa, Joice Tome, Rachel Makasi, Wellington Murenjekekwa, Theodore Chidawanyika, Blessing Tsenesa, Stephen Moyo, Penias Nyamwino, Pururudzai Simango, Shepherd Seremwe, Lovemore Chingaoma and Sarudzai Kasaru. *Lusaka, Zambia*: Andreck Tembo, Mary Mpundu, Evelyn Nyendwa, Gwendolyn Nayame, Dalitso Tembo, Sophreen Mwaba, Esther Chilala, Lucy Macwani, Tenzeni Dumba, Miyoba Chipunza, Lydia Kazhila, Temwaninge Gondwe, Dennis Phiri, Mpala Mwanza, Kanekwa Zyambo.

We thank Philippa Rambanepasi, Karen Gwanzura and Agatha Muyenga for financial management of the study; Zinah Sorefan and Daniela Azurunwa for study coordination at Queen Mary University of London; Professor Kim Michaelsen for the use of the electronic knemometers and advice on their use; and we remember the hard work of Edith Mukusho who sadly passed away during the HOPE-SAM study. We thank the staff and management and members of the Department of Paediatrics and Child Health at the three hospital sites who have made this study possible. We are indebted to the caregivers, families and children who are participating in the HOPE-SAM study.

For peer review only - http://bmjopen.bmj.com/site/about/guidelines.xhtml

AUTHOR CONTRIBUTIONS

Designed study: MB-D, BA, CDB, RCR, BM, KC, CK, KCh, DN, PC, NC, FM, JW, ARM, JS, ASW, KJN, PK, AJP

Sought funding: MB-D, BA, CDB, RCR, JHH, ARM, JS, ASW, KJN, PK, AJP

Undertaking study: BM, KC, CK, KCh, FM, DN, PC, NC, FM, IM, EB, KM, SM, TR

Study oversight: MB-D, BA, JHH, KJN, PK, AJP

Analysis: MB-D, BA, CDB, RCR, RN, JW, ARM, JS, ASW, KJN, PK, AJP

Wrote first draft of manuscript: MB-D, CDB, RCR, AJP

Critically revised manuscript: All

FUNDING

This work was supported by the Medical Research Council UK (MR/K012711/1), the Wellcome Trust (107634/Z/15/Z to MB-D; 206225/Z/17/Z to CDB, an award funded in partnership with the Royal Society; 206455/Z/17/Z to RCR; and 108065/Z/15/Z to AJP), and a Bio-Resource Grant from the Centre for Genomic Health within the Life Sciences Initiative at Queen Mary University of London.

COMPETING INTERESTS

None of the authors have any competing interests to declare.

For peer review only

REFERENCES

1. Black R E, Victora C G, Walker S P, et al. Maternal and child undernutrition and overweight in low-income and middle-income countries. *Lancet* 2013;382:427–51.

2. World Health Organisation G. Guideline Updates on the management of severe acute malnutrition in infants and children: World Health Organisation, Geneva, 2013.

3. Lenters LM, Wazny K, Webb P, et al. Treatment of severe and moderate acute malnutrition in low- and middle-income settings: a systematic review, meta-analysis and Delphi process. *BMC Public Health* 2013;13 (Supp 3):S23.

4. Golden MHN. Oedematous malnutrition. *British Medical Bulletin* 1998;54(2):433-44.

5. Schofield C, A. A. Why have mortality rates for severe malnutrition remained so high? *Bull World Health Organ* 1996;74(2):223-29.

6. Munthali T, Jacobs C, Sitali L, et al. Mortality and morbidity patterns in under-five children with severe acute malnutrition (SAM) in Zambia: a five-year retrospective review of hospital-based records (2009–2013). *Archives of Public Health* 2015;73(23)

7. Preidis G A, McCollum E D, Mwansambo C, et al. Pneumonia and Malnutrition are Highly Predictive of Mortality among African Children Hospitalized with Human Immunodeficiency Virus Infection or Exposure in the Era of Antiretroviral Therapy. *Journal of Pediatrics* 2011;159(3):484-89.

8. World Health Organisation G. Global guidance on criteria and processes for validation: Elimination of Mother-to-Child transmission of HIV and Syphilis. Geneva: World Health Organization, 2017.

9. Ceri E, Jones C-E, AJ. P. HIV-exposed, uninfected infants: new global challenges in the era of paediatric HIV elimination. *Lancet Infectious Diseases* 2016 doi: 10.1016/S1473

For peer review only

For peer review only - http://bmjopen.bmj.com/site/about/guidelines.xhtml

10. Heikens GT, Bunn J, Amadi A, et al. Case management of HIV-infected severely malnourished children: challenges in the area of highest prevalence. *Lancet* 2008;371:1305-7.

11. Heikens GT, Bunn J, Amadi B, et al. Case management of HIV-infected severely malnourished children: challenges in the area of highest prevalence. *Lancet* 2008;371(9620):1305-7.

12. Kerac M, Bunn J, George Chagaluka G, et al. Follow-Up of Post-Discharge Growth and Mortality after Treatment for Severe Acute Malnutrition (FuSAM Study): A Prospective Cohort Study. *Plos One* 2014;9(6)

13. Lelijveld M, Seal A, Wells J C, et al. Chronic disease outcomes after severe acute malnutrition in Malawian children (ChroSAM): a cohort study. *Lancet Global Health* 2016;4:e654-62.

14. Moïsi JC, Gatakaa H, Berkley JA, et al. Excess child mortality after discharge from hospital in Kilifi, Kenya: a retrospective cohort analysis. *Bull World Health Organ* 2011;89:725-32A.

15. Wiens MO, Pawluk S, Kissoon N, et al. Pediatric Post-Discharge Mortality in Resource Poor Countries: A Systematic Review. *Plos One* 2013;8(6)

16. Fergusson P, Chinkhumba J, Grijalva-Eternod C, et al. Nutritional recovery in HIV-infected and HIVuninfected children with severe acute malnutrition. *Archives of Diseases in Children* 2009;94:512-16.

17. DeBoer M D, Lima A A M, Oría R B, et al. Early childhood growth failure and the developmental origins of adult disease: Do enteric infections and malnutrition increase risk for the metabolic syndrome? *Nutrition Reviews* 2012 70(11):642-53.

18. Barouki R, Gluckman P D, Grandjean P, et al. Developmental origins of non-communicable disease: Implications for research and public health. *Environmental Health* 2012;11(42)

For peer review only

19. Davey Smith G, Greenwood R, Gunnell D, et al. Leg length, insulin resistance, and coronary heart disease risk: The Caerphilly Study. *Journal of Epidemiology Community Health* 2001;55:867-72.

20. Gunnell D, Whitley E, Upton MN, et al. Associations of height, leg length, and lung function with cardiovascular risk factors in the Midspan Family Study. *Journal of Epidemiology Community Health* 2002;57:141-46.

21. Jones K D, Thitiri J, Ngari M, et al. Childhood malnutrition: Toward an understanding of infections, inflammation, and antimicrobials. *Food and Nutrition Bulletin* 2014;35(2)

22. Attia S, Versloot CJ, Voskuijl W, et al. Mortality in children with complicated severe acute malnutrition is related to intestinal and systemic inflammation: an observational cohort study. *American Journal of Clinical Nutrition* 2016;104:1441-9.

23. Bourke CD, Berkley J A, A. P. Immune Dysfunction as a Cause and Consequence of Malnutrition. *Trends in Immunology* 2016;37(6):386-89.

24. Smith MI, Yatsunenko T, Manary MJ, et al. Gut microbiomes of Malawian twin pairs discordant for kwashiorkor. *Science* 2013;339(6119):548-54.

25. Di Giovanni V, Bourdon C, Wang DX, et al. Metabolomic Changes in Serum of Children with Different Clinical Diagnoses of Malnutrition. *Journal of Nutrition* 2016;146:2436-44.

26. Rytter MJH, Kolte L, Briend A, et al. The Immune System in Children with Malnutrition—A Systematic Review. *Plos One* 2014;9(8)

27. Smith MI, Yatsunenko T, Manary MJ, et al. Gut microbiomes of Malawian twin pairs discordant for kwashiorkor. *Science (New York, NY)* 2013;339(6119):548-54.

28. Blanton LV, Charbonneau MR, Salih T, et al. Gut bacteria that prevent growth impairments transmitted by microbiota from malnourished children. *Science* 2016;351(6275)

29. Subramanian S, Huq S, Yatsunenko T, et al. Persistent gut microbiota immaturity in malnourished Bangladeshi children. *Nature* 2014;510(7505):417-21.

For peer review only

30. Prendergast A, Kelly P. Review: Enteropathies in the developing world: neglected effects on global health. *Am J Trop Med Hyg* 2012;86:756-63.

31. Lutwama F, Kagina BM, Wajja A, et al. Distinct T-Cell Responses When BCG Vaccination Is Delayed From Birth to 6 Weeks of Age in Ugandan Infants. *The Journal of Infectious Diseases* 2014;209(6):887-97.

32. Prendergast AJ, Humphrey JH, Mutasa K, et al. Assessment of Environmental Enteric Dysfunction in the SHINE Trial: Methods and Challenges. *Clin Infect Dis* 2015;61 (suppl 7):S685-702.

33. World Health Organization. Management of severe malnutrition: a manual for physicians and other senior health workers. WHO, Geneva, 1999. Available athttp://www.who.int/nutrition/publications/severemalnutrition/9241545119/en/.

34. World Health Organization. Consolidated guidelines on the use of antiretroviral drugs for treating and preventing HIV infection: Recommendations for a public health approach. WHO, Geneva, 2003. Available at http://www.who.int/hiv/pub/guidelines/arv2013/download/en/.

35. Costiniuk CT, JB. A. Human immunodeficiency virus and the gastrointestinal immune system: does highly active antiretroviral therapy restore gut immunity? *Nature* 2012;5(6):596-604.

36. Brenchley JM, Douek DC. HIV infection and the gastrointestinal immune system. *Nature* 2008;1(1) doi: doi:10.1038/mi.2007.1

37. Amadi B, Besa E, Zyambo K, et al. Impaired Barrier Function and Autoantibody Generation in Malnutrition Enteropathy in Zambia. *EBioMedicine* 2017;22(Supplement C):191-99. doi: https://doi.org/10.1016/j.ebiom.2017.07.017

38. Kau AL, Ahern PP, Griffin NW, et al. Human nutrition, the gut microbiome and the immune system. *Nature* 2011;474(7351):327-36.

39. Blanton LV, Charbonneau MR, Salih T, et al. Gut bacteria that prevent growth impairments transmitted by microbiota from malnourished children. *Science* 2016;351(6275) doi: 10.1126/science.aad3311

For peer review only

40. Kau AL, Planer JD, Liu J, et al. Functional characterization of IgA-targeted bacterial taxa from undernourished Malawian children that produce diet-dependent enteropathy. *Sci Transl Med* 2015;7(276):276ra24. doi: 10.1126/scitranslmed.aaa4877

41. Subramanian S, Huq S, Yatsunenko T, et al. Persistent gut microbiota immaturity in malnourished Bangladeshi children. *Nature* 2014;510(7505):417-21. doi: 10.1038/nature13421

42. Monaco CL, Gootenberg DB, Zhao G, et al. Altered Virome and Bacterial Microbiome in Human Immunodeficiency Virus-Associated Acquired Immunodeficiency Syndrome. *Cell Host Microbe* 2016;19(3):311-22. doi: 10.1016/j.chom.2016.02.011

43. Amadi B, Fagbemi AO, Kelly P, et al. Reduced production of sulfated glycosaminoglycans occurs in Zambian children with kwashiorkor but not marasmus. *Am J Clin Nutr* 2009;89(2):592-600. doi: 10.3945/ajcn.2008.27092

44. Segata N, Waldron L, Ballarini A, et al. Metagenomic microbial community profiling using unique clade-specific marker genes. *Nature methods* 2012;9(8):811-4. doi: 10.1038/nmeth.2066

45. Abubucker S, Segata N, Goll J, et al. Metabolic reconstruction for metagenomic data and its application to the human microbiome. *PLoS computational biology* 2012;8(6):e1002358. doi: 10.1371/journal.pcbi.1002358

46. Bartz S, Mody A, Hornik C, et al. Severe Acute Malnutrition in Childhood: Hormonal and Metabolic Status at Presentation, Response to Treatment and Predictors of Mortality. *Journal of Clinical and Endocrine Metabolism* 2014;99(6):2128-37.

47. Farras M, Chandwe K, Mayneris-Perxachs J, et al. Characterizing the metabolic phenotype of intestinal villus blunting in Zambian children with severe acute malnutrition and persistent diarrhoea. *Plos One* 2018, in press

For peer review only

For peer review only - http://bmjopen.bmj.com/site/about/guidelines.xhtml

48. Mayneris-Perxachs J, Lima AA, Guerrant RL, et al. Urinary N-methylnicotinamide and β-aminoisobutyric acid predict catch-up growth in undernourished Brazilian children. *Sci Rep* 2016;6:19780. doi: 10.1038/srep19780

49. Manary MJ, Leeuwenburgh C, Heinecke JW. Increased oxidative stress in kwashiorkor. *J Pediatr* 2000;137(3):421-4.

50. Attia S, Versloot CJ, Voskuijl W, et al. Mortality in children with complicated severe acute malnutrition is related to intestinal and systemic inflammation: an observational cohort study. *The American Journal of Clinical Nutrition* 2016 doi: 10.3945/ajcn.116.130518

51. Chisti MJ, Salam MA, Bardhan PK, et al. Severe Sepsis in Severely Malnourished Young Bangladeshi Children with Pneumonia: A Retrospective Case Control Study. *PLoS ONE* 2015;10(10):e0139966. doi: 10.1371/journal.pone.0139966

52. Page A-L, de Rekeneire N, Sayadi S, et al. Infections in Children Admitted with Complicated Severe Acute Malnutrition in Niger. *PLoS ONE* 2013;8(7):e68699. doi: 10.1371/journal.pone.0068699

53. Jones KDJ, Berkley JA. Severe acute malnutrition and infection. *Paediatrics & International Child Health* 2014;34(Suppl 1):1-29. doi: 10.1179/2046904714Z.000000000218

54. Bahwere P, Levy J, Hennart P, et al. Community-acquired bacteremia among hospitalized children in rural Central Africa. *International Journal of Infectious Diseases* 2001;5(4):180-88.

55. Thuo N, Ohuma E, Karisa J, et al. The prognostic value of dipstick urinalysis in children admitted to hospital with severe malnutrition. *Arch Dis Child* 2010;95(6):422-6.

56. Berkley JA, Lowe BS, Mwangi I, et al. Bacteremia among Children Admitted to a Rural Hospital in Kenya. *New England Journal of Medicine* 2005;352(1):39-47.

For peer review only

57. Chang CY, Trehan I, Wang RJ, et al. Children Successfully Treated for Moderate Acute Malnutrition Remain at Risk for Malnutrition and Death in the Subsequent Year after Recovery. *The Journal of nutrition* 2013;143(2):215-20.

58. Kerac M, Bunn J, Chagaluka G, et al. Follow-Up of Post-Discharge Growth and Mortality after Treatment for Severe Acute Malnutrition (FuSAM Study): A Prospective Cohort Study. *PLoS ONE* 2014;9(6):e96030. doi: 10.1371/journal.pone.0096030

59. Berkley JA, Ngari M, Thitiri J, et al. Daily co-trimoxazole prophylaxis to prevent mortality in children with complicated severe acute malnutrition: a multicentre, double-blind, randomised placebo-controlled trial. *The Lancet Global Health* 2016;4(7):e464-e73. doi: 10.1016/S2214-109X(16)30096-1

60. Bourke CD, Berkley JA, Prendergast AJ. Immune Dysfunction as a Cause and Consequence of Malnutrition. *Trends Immunol* 2016 doi: 10.1016/j.it.2016.04.003

61. Rytter MJ, Kolte L, Briend A, et al. The immune system in children with malnutrition--a systematic review. *PLoS One* 2014;9(8):e105017.

62. Ibrahim MK, Zambruni M, Melby CL, et al. Impact of Childhood Malnutrition on Host Defense and Infection. *Clinical Microbiology Reviews* 2017;30(4):919-71.

63. Bartz S, Mody A, Hornik C, et al. Severe acute malnutrition in childhood: hormonal and metabolic status at presentation, response to treatment, and predictors of mortality. *Journal of Clinical Endocrinology & Metabolism* 2014;99(6):2128-37.

64. Hughes SM, Amadi B, Mwiya M, et al. Dendritic cell anergy results from endotoxemia in severe malnutrition. *Journal of immunology* 2009;183(4):2818-26.

65. Jones KD, Hunten-Kirsch B, Laving AM, et al. Mesalazine in the initial management of severely acutely malnourished children with environmental enteric dysfunction: a pilot randomized controlled trial. *BMC Medicine* 2014;12:133. doi: 10.1186/s12916-014-0133-2

For peer review only

66. Prendergast A, Kelly P. Enteropathies in the developing world: neglected effects on global health. *American Journal of Tropical Medicine & Hygiene* 2012;86(5):756-63.

67. Watanabe K, Petri WA, Jr. Environmental Enteropathy: Elusive but Significant Subclinical Abnormalities in Developing Countries. *EBioMedicine* 2016 doi: 10.1016/j.ebiom.2016.07.030

68. Kelly P, Besa E, Zyambo K, et al. Endomicroscopic and Transcriptomic Analysis of Impaired Barrier Function and Malabsorption in Environmental Enteropathy. *PLoS Negl Trop Dis* 2016;10(4):e0004600.

69. Behrens RH, Lunn PG, Northrop CA, et al. Factors affecting the integrity of the intestinal mucosa of Gambian children. *American Journal of Clinical Nutrition* 1987;45:1433-41.

70. Di Giovanni V, Bourdon C, Wang DX, et al. Metabolomic Changes in Serum of Children with Different Clinical Diagnoses of Malnutrition. *J Nutr* 2016 doi: 10.3945/jn.116.239145

71. Borm GF, Fransen J, Lemmens WA. A simple sample size formula for analysis of covariance in randomized clinical trials. *J Clin Epidemiol* 2007;60(12):1234-8.

72. Girma T, Kæstel P, Workeneh N, et al. Bioimpedance index for measurement of total body water in severely malnourished children: Assessing the effect of nutritional oedema. *Clinical Nutrition* 2015

73. Girma T, Hother Nielsen AL, Kæstel P, et al. Biochemical and anthropometric correlates of bio-electrical impedance parameters in severely malnourished children: A cross-sectional study. *Clinical Nutrition* 2017 doi: 10.1016/j.clnu.2017.02.017

74. Bourke CD, Nausch N, Rujeni N, et al. Integrated Analysis of Innate, Th1, Th2, Th17, and Regulatory Cytokines Identifies Changes in Immune Polarisation Following Treatment of Human Schistosomiasis. *The Journal of Infectious Diseases* 2013;208(1):159-69.

For peer review only

For peer review only - http://bmjopen.bmj.com/site/about/guidelines.xhtml

75. Prendergast AJ, Szubert AJ, Berejena C, et al. Baseline Inflammatory Biomarkers Identify Subgroups of HIV-Infected African Children With Differing Responses to Antiretroviral Therapy. *Journal of Infectious Diseases* 2016;214:226-36

76. Bourke CD, Mutapi F, Nausch N, et al. Trichuris suis ova therapy for allergic rhinitis does not affect allergen-specific cytokine responses despite a parasite-specific cytokine response. *Clinical and Experimental Allergy* 2012;42:1582-95.

77. Bourke CD, Nausch N, Rujeni N, et al. Cytokine Responses to the Anti-schistosome Vaccine Candidate Antigen Glutathione-S-transferase Vary with Host Age and Are Boosted by Praziquantel Treatment. *Plos Neglected Tropical Diseases* 2014;8(5)

78. Fergusson P, A. T. HIV prevalence and mortality among children undergoing treatment for severe acute malnutrition in sub-Saharan Africa: a systematic review and meta-analysis. *Transactions of the Royal Society of Tropical Medicine and Hygiene* 2009;103(6):541-48.

79. Schofiled C, A. A. Why have mortality rates for severe malnutrition remained so high? *Bulletin of the World Health Organization* 1996;74(2):223-29.

80. Chinkhumba J, Tomkins A, Banda C, et al. The impact of HIV on mortality during in-patient rehabilitation of severely malnourished children in Malawi. *Transactions of the Royal Society of Tropical Medicine and Hygiene* 2008;102(7):639-44.

For peer review only

FIGURE LEGENDS

Figure 1: **Study flow chart.**

All hospital admissions are screened for eligibility for the observational cohort and enteropathy substudy, with procedures undertaken as shown in the flow chart during hospitalisation and post-discharge. Well-nourished children from outpatient clinics meeting eligibility criteria as healthy controls are enrolled and undergo a single baseline assessment as shown.

For peer review only

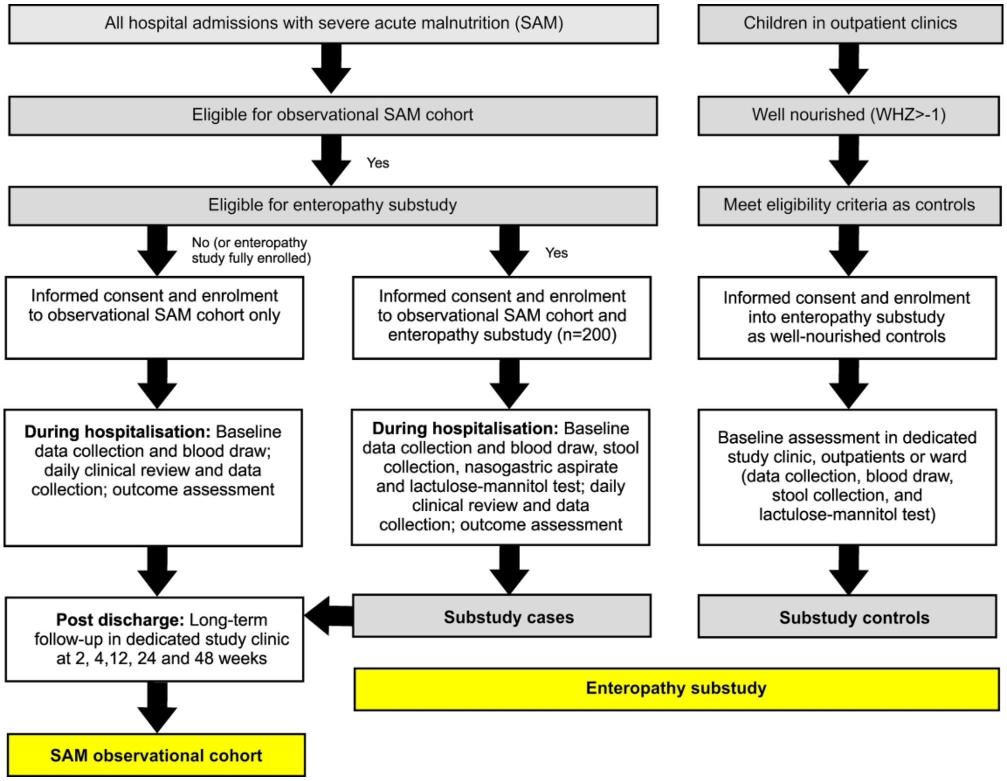

197x152mm (300 x 300 DPI)

Supplementary Table 1: Assays undertaken on stored samples for children in the observational cohort.

Sample type	Assay (method)	Location of work	Study subjects	Time-points
Blood	HIV testing (rapid antibody test algorithm if >18 mo old or HIV DNA PCR <18 mo old)[1]	TROPGAN, Zvitambo or clinical sites	All	Baseline
Blood	CD4 count (flow cytometry or PIMA)[1]	TROPGAN, Zvitambo or clinical sites	All HIV-positive	Baseline, wk 12, 24, 48
Plasma	HIV viral load (real-time PCR)[1]	TROPGAN, Zvitambo or clinical sites	All HIV-positive	Baseline, wk 12, 24, 48
Plasma	C-reactive protein (ELISA)	TROPGAN, Zvitambo	All	Baseline, discharge, wk 12, 24, 48
Plasma	Albumin (ELISA)	TROPGAN, Zvitambo	All	Baseline, discharge, wk 12, 24, 48
Plasma	Lipopolysaccharide (LAL assay)	TROPGAN and Zvitambo	Subgroup[3]	Baseline, discharge, wk 12, 24, 48
Plasma	Lipopolysaccharide binding protein (LBP)	TROPGAN and Zvitambo	Subgroup[3]	Baseline, discharge, wk 12, 24, 48
Plasma	sCD14 (ELISA)	TROPGAN and Zvitambo	Subgroup[3]	Baseline, discharge, wk 12, 24, 48
Plasma	sCD163 (ELISA)	TROPGAN and Zvitambo	Subgroup[3]	Baseline, discharge, wk 12, 24, 48
Plasma	IL-6, TNF-alpha, IL-1β (ELISA) and/or multiplex cytokines	TROPGAN and Zvitambo	Subgroup[3]	Baseline, discharge, wk 12, 24, 48
Plasma	Total PAMP activity (THP1 reporter cell line[2])	TROPGAN and Zvitambo	Subgroup[3]	Baseline, discharge, wk 12, 24, 48
Whole blood	Molecular techniques for bacterial detection (broad-range and specific PCR and next-generation sequencing)	QMUL, London[2]	Subgroup[3]	Baseline, discharge, wk 12, 24, 48
Whole blood	*In vitro* binding to bacterial products, cytokine expression and cellular responses to PAMP stimulation[4]	TROPGAN, Zvitambo	All	Baseline, discharge, wk 12, 24, 48
Plasma	Co-culture with healthy immune cells[5]	Blizard Institute	All	Baseline, discharge, wk 12, 24, 48

[1]If HIV test, CD4 and viral load have already been conducted as part of routine clinical care, they will not be repeated on the research sample.
[2]THP1 reporter cells are derived from THP1, a human monocytic cell line that naturally expresses many pattern recognition receptors (PRR). The cell line stably expresses an NF-κB/AP-1 inducible reporter (SEAP) system to facilitate the monitoring of PRR-induced NF-κB/AP-1 activation.
[3]Assays will be undertaken in a subgroup of children, using a case-control or case-cohort design to evaluate the impact of biomarkers on immune activation and mortality.
[4]Whole blood will be stimulated with pathogen-associated molecular patterns (PAMP) in culture plates and bacterial antigens labelled with fluorescent tags in test tubes, and incubated for 1-24hr. Supernatant will be removed and stored at -80C for subsequent analysis of pro- and anti-inflammatory cytokines, and cells will be fixed as described in section 12.2.2 for subsequent analysis of bacterial binding, cellular activation, proliferation and cytokine elaboration by flow cytometry.
[5]To determine the effect of the systemic milieu on healthy immune cell function, plasma samples will be transported to the Blizard Institute and co-cultured with healthy immune cells, which will be functionally analysed via multi-parameter flow cytometry in the Flow Cytometry Core Facility.

IFABP: Intestinal fatty acid binding protein; ELISA: Enzyme-linked immunosorbent assay; GLP-2: glucagon-like peptide 2; sCD14: soluble CD14; sCD163: soluble CD163; PAMP: pathogen-associated molecular pattern; QMUL: Queen Mary University of London; CRP: C-reactive protein; LAL: limulus amoebocyte lysate assay.

For peer review only

Supplementary table 2: Additional laboratory analyses for enteropathy substudy

Sample type	Assay (method)	Location of work	Study groups[1]	Time-points
Urine	Lactulose-mannitol ratio (mass spectrometry)	Orgeon Analytics, USA[2]	A, B, C, D (all)	Baseline, discharge, wk 12 and 48
Stool	Neopterin, myeloperoxidase, alpha-1 antitrypsin and REG-1B (ELISA)	TROPGAN and Zvitambo	A, B, C, D (all)	Baseline, discharge, wk 12, 24, 48
Plasma	I-FABP (ELISA)	TROPGAN and Zvitambo	A, B, C, D (all)	Baseline, discharge, wk 12, 24, 48
Plasma	GLP-2	TROPGAN and Zvitambo	A, B, C, D (all)	Baseline, discharge, wk 12, 24, 48
Plasma	Citrulline (mass spectrometry)	Imperial College London	A, B, C, D (all)	Baseline, discharge, wk 12, 24, 48
Plasma	Kynurenine:tryptophan ratio and metabolites along tryptophan pathway (mass spectrometry)	Imperial College London	A, B, C, D (all)	Baseline, discharge, wk 12, 24, 48
Stool	Microbiome analysis[3]	BCCDC, Vancouver[2]	A, B, C, D (all)	Baseline, discharge, wk 12, 24, 48
Stool	*Helicobacter pylori* antigen	TROPGAN and Zvitambo	A, B, C, D (all)	Baseline
Gastric juice	Culture and molecular techniques for bacterial detection (broad-range and specific PCR and next-generation sequencing)	QMUL, London[2]	Subgroup of A, C (n=50 per group)	Baseline
RNA extracted from PAXGene tubes	Gene expression analysis (RNASeq)	QMUL, London[2]	A, B, C, D (all)	Baseline and discharge
Plasma and urine	Targeted and untargeted metabolic phenotyping	Imperial College London[2]	A, B, C, D (all)	Baseline, discharge, wk 12, 24 and 48

For peer review only - http://bmjopen.bmj.com/site/about/guidelines.xhtml

[1]Enteropathy substudy groups are shown in **Table 1.** Note that controls only have blood taken at baseline as per Table 4.
[2]Assay methodology not available in country. See section 12.1.4 for details of shipment to UK/USA/Canada.
[3]For microbiome analyses,total DNA and/or RNA will be extracted from stool samples and used as template for next generation sequencing library preparation and for quantitative polymerase chain reaction (qPCR), then sequenced via whole metagenome shotgun sequencing (see section 12.3.3.2).

QMUL: Queen Mary University of London; BCCDC: British Columbia Centre for Disease Control.

For peer review only

BMJ Open

Health Outcomes, Pathogenesis and Epidemiology of Severe Acute Malnutrition (HOPE-SAM): rationale and methods of a longitudinal observational study

Journal:	*BMJ Open*
Manuscript ID	bmjopen-2018-023077.R1
Article Type:	Protocol
Date Submitted by the Author:	03-Aug-2018
Complete List of Authors:	Bwakura-Dangarembizi, Mutsa; University of Zimbabwe College of Health Sciences, Paediatrics and Child Health<br>Amadi, Beatrice; 2Tropical Gastroenterology and Nutrition Group, University of Zambia, Lusaka, Zambia<br>Bourke, Claire ; Blizard Institute, Queen Mary University of London, UK<br>Robertson, Ruairi; Blizard Institute, Queen Mary University of London, UK<br>Mwapenya, Benjamin; Zvitambo Institute for Maternal and Child Health Research, Harare, Zimbabwe<br>Chandwe, Kanta; Tropical Gastroenterology and Nutrition Group, University of Zambia, Lusaka, Zambia<br>Kapoma, Chanda; Tropical Gastroenterology and Nutrition Group, University of Zambia, Lusaka, Zambia, Paediatrics; Livingstone Central Hospital, Paediatrics<br>Chifunda, Kapula; Tropical Gastroenterology and Nutrition Group, University of Zambia, Lusaka, Zambia, paediatrics and child health<br>Majo, Florence; Zvitambo Institute for Maternal and Child Health Research, Harare, Zimbabwezi<br>Ngosa, Deophine; Tropical Gastroenterology and Nutrition Group, University of Zambia, Lusaka, Zambia<br>Chakara, Pamela; Zvitambo Institute for Maternal and Child Health Research, Harare, Zimbabwe<br>Chulu, Nivea; 2Tropical Gastroenterology and Nutrition Group, University of Zambia, Lusaka, Zambia<br>Masimba, Faithfull; Zvitambo Institute for Maternal and Child Health Research, Harare, Zimbabwe<br>Mapurisa, Idah; Zvitambo Institute for Maternal and Child Health Research, Harare, Zimbabwe<br>Besa, Ellen; Zvitambo Institute for Maternal and Child Health Research, Harare, Zimbabwe<br>Mutasa, Kuda; Zvitambo Institute for Maternal and Child Health Research, Harare, Zimbabwe<br>Mwakamui, Simutanyi; 2Tropical Gastroenterology and Nutrition Group, University of Zambia, Lusaka, Zambia<br>Runodamoto, Thompson ; Zvitambo Institute for Maternal and Child Health Research, Harare, Zimbabwe<br>Humphrey, Jean; Zvitambo Institute for Maternal and Child Health Research, Harare, Zimbabwe<br>Ntozini, Robert; Zvitambo Institute, Biostatistics & IT

	Wells, Jonathan C. K.; UCL Great Ormond Street Institute of Child Health<br>Manges, Amee; University of British Columbia<br>Swann, Jon; Imperial College London<br>Walker, Sarah; MRC Clinical Trials Unit at UCL<br>Nathoo, Kusum; University of Zimbabwe, College of Health Sciences<br>Kelly, Paul; Barts and The London School of Medicine<br>Prendergast, Andrew ; Queen Mary University of London,

**Primary Subject Heading**:	Global health
Secondary Subject Heading:	Epidemiology, Nutrition and metabolism, Paediatrics, Immunology (including allergy)
Keywords:	Malnutrition, HIV, Africa, mortality, microbiota, enteropathy

SCHOLARONE™ Manuscripts

Health Outcomes, Pathogenesis and Epidemiology of Severe Acute Malnutrition (HOPE-SAM): rationale and methods of a longitudinal observational study

Mutsa Bwakura-Dangarembizi[1], Beatrice Amadi[2], Claire D Bourke[3], Ruairi C Robertson[3], Benjamin Mwapenya[4], Kanta Chandwe[2], Chanda Kapoma[2], Kapula Chifunda[2], Florence Majo[4], Deophine Ngosa[2], Pamela Chakara[4], Nivea Chulu[2], Faithfull Masimba[4], Idah Mapurisa[4], Ellen Besa[2], Kuda Mutasa[4], Simutanyi Mwakamui[2], Thompson Runodamoto[4], Jean H Humphrey[4], Robert Ntozini[4], Jonathan Wells[5], Amee R Manges[6], Jon R Swann[7], A Sarah Walker[8], Kusum J Nathoo[1], Paul Kelly[2,3], Andrew J Prendergast[3,4] for the HOPE-SAM Study Team[9].

[1]Department of Paediatrics and Child Health, University of Zimbabwe College of Health Sciences, Harare, Zimbabwe

[2]Tropical Gastroenterology and Nutrition Group, University of Zambia, Lusaka, Zambia

[3]Blizard Institute, Queen Mary University of London, UK

[4]Zvitambo Institute for Maternal and Child Health Research, Harare, Zimbabwe

[5]UCL Great Ormond Street Institute of Child Health, London, UK

[6]University of British Columbia, Vancouver, Canada

[7]Imperial College London, UK

[8]MRC Clinical Trials Unit at UCL, London, UK

[9]Other members of the HOPE-SAM study team are listed in the Acknowledgements section.

Corresponding author: Dr Mutsa Bwakura-Dangarembizi, Senior Lecturer, Department of Paediatrics and Child Health, University of Zimbabwe College of

For peer review only

Health Sciences, Box A178, Avondale, Harare, Zimbabwe. Email: mbwakura@medsch.uz.ac.zw. Tel +263 772 601 735. Fax +263 4 700 877.

Word count 5926

Abstract 261

Key words: Malnutrition, HIV, Africa, mortality, microbiota, enteropathy, immunology

For peer review only

ABSTRACT

Introduction

Mortality among children hospitalised for complicated severe acute malnutrition (SAM) remains high despite the implementation of WHO guidelines, particularly in settings of high HIV prevalence. Children continue to be at high risk of morbidity, mortality and relapse after discharge from hospital although long-term outcomes are not well documented. Better understanding the pathogenesis of SAM and the factors associated with poor outcomes may inform new therapeutic interventions.

Methods and analysis

The Health Outcomes, Pathogenesis and Epidemiology of Severe Acute Malnutrition (HOPE-SAM) study is a longitudinal observational cohort that aims to evaluate the short- and long-term clinical outcomes of HIV-positive and HIV-negative children with complicated SAM, and to identify the risk factors at admission and discharge from hospital that independently predict poor outcomes. Children aged 0-59mo hospitalised for SAM are being enrolled at three tertiary hospitals in Harare, Zimbabwe, and Lusaka, Zambia. Longitudinal mortality, morbidity and nutritional data are being collected at admission, discharge and for 48 weeks post-discharge. Nested laboratory substudies are exploring the role of enteropathy, gut microbiota, metabolomics and cellular immune function in the pathogenesis of SAM using stool, urine and blood collected from participants and from well-nourished controls.

Ethics and dissemination

The study is approved by the local and international institutional review boards in the participating countries (the Joint Research Ethics Committee of the University of Zimbabwe, Medical Research Council of Zimbabwe and University of Zambia Biomedical Research Ethics Committee) and the study sponsor (Queen Mary University of London). Caregivers provide written informed consent for each

For peer review only

participant. Findings will be disseminated through peer-reviewed journals, conference presentations and to caregivers at face-to-face meetings.

Strengths and limitations of this study

Strengths:

- The primary strength of this study is the rigorous collection of longitudinal data on morbidity, mortality and nutritional status during inpatient care and for 48 weeks after initial admission for SAM.
- Laboratory sub-studies investigating enteropathy, microbiota, metabolomics and immune cell function provide a unique opportunity to understand which pathogenic pathways contribute to SAM and whether these processes normalise with nutritional rehabilitation, capitalising on a well-characterised cohort with inclusion of well-nourished controls.
- This study will compare longitudinal clinical outcomes among HIV-negative and HIV-positive children with SAM in the current ART era.

Potential limitations:

- High loss to follow-up due to participants returning to home settings following hospital discharge. A dedicated clinical study team is in place to maximise follow-up through phone reminders and community visits.
- The clinical heterogeneity of the study participants, including comorbidities such as infections, may make it challenging to identify the specific causes of clinical outcomes. However, the embedded sub-studies will enable multiple pathways to be explored within the same cohort.
- Potential bias in recruiting well-nourished controls only from hospitals will be reduced by inclusion of community-based controls, including well-nourished siblings of children with SAM.

For peer review only - http://bmjopen.bmj.com/site/about/guidelines.xhtml

INTRODUCTION

Malnutrition underlies almost half of all childhood deaths in developing countries[1]. Severe acute malnutrition (SAM) is defined by a weight-for-height Z-score <-3, mid-upper arm circumference (MUAC) <115mm and/or bilateral pitting oedema[2]. Current treatment guidelines distinguish two groups: i) children with uncomplicated SAM who can be managed in the community; and ii) children with complicated SAM, who are hospitalised and undergo resuscitation, stabilisation and nutritional rehabilitation. In-hospital mortality in children with complicated SAM remains high despite the implementation of WHO guidelines[3]. Furthermore, SAM presents as two major clinical phenotypes: non-oedematous SAM (marasmus), characterised by severe wasting, and oedematous SAM (kwashiorkor), a more complex syndrome characterised by bilateral pitting oedema, steatosis and diarrhea[4 5]. Despite differing clinical outcomes, treatment protocols are the same for both oedematous and non-oedematous SAM.

A contributory factor to high in-patient mortality is the co-occurrence of HIV infection in around one-third of children hospitalised for SAM in sub-Saharan Africa[6 7]. While new HIV infections in children have declined[8], a substantial number of infected children are diagnosed late and present with malnutrition. There is also a growing population of HIV-exposed uninfected (HEU) children who have immune abnormalities, poor growth and higher risk of mortality and infectious morbidity[9]. Hence, HIV has transformed the epidemiology and outcomes of SAM[10]. Even with standardised treatment approaches, inpatient deaths are almost four-fold higher among HIV-positive children with SAM (herein termed HIV-SAM), compared to HIV-negative children with SAM (30.4% vs 8.4%), for reasons that remain unclear[10]; this mortality is three-fold higher than would be expected from anthropometric parameters alone[10]. Management of HIV-SAM is particularly challenging because HIV fundamentally alters the clinical presentation of malnutrition and the response to

For peer review only

treatment. Children with HIV-SAM are more stunted and wasted; have a higher frequency of persistent diarrhoea; tend to have delayed nutritional recovery and have a more complicated clinical course than HIV-negative children with SAM[10].

Long-term outcomes of SAM

Following resolution of complications and return of appetite, children are discharged from hospital to continue therapeutic feeds at home. However, emerging data indicate high post-discharge mortality following in-hospital management of SAM[11-13]. Malnutrition together with young age, HIV infection and pneumonia have been associated with higher post-discharge mortality[14]. One of the largest prospective studies of growth and mortality in children with SAM (FuSAM), conducted in Malawi from July 2006 to March 2007, collected 12-month outcome data on 87% of 1024 children admitted to the nutrition ward[11]. A total of 427 (42%) died and 44% of these deaths occurred after discharge from hospital. Survival was greatest among those who were nutritionally cured upon discharge from outpatient therapeutic feeding centres, defined as two consecutive visits with >80% expected weight-for-height, no oedema and clinically stable. The risk of mortality after hospital discharge was four-fold higher for HIV-SAM compared to HIV-negative children with SAM, but the outcomes among HEU children were not reported. The loss to follow-up was high in the FuSAM study because there was only one follow-up visit, one year after discharge from outpatient-feeding centres. A recent study from Kenya identified malnutrition and HIV infection as key drivers for post-discharge mortality, with 52% of deaths attributable to MUAC <11.5cm and 11% to HIV infection[15].

The impact of SAM appears to persist beyond the first year after discharge from hospital. The ChroSAM study, which followed children with SAM seven years post-discharge, showed that children had poorer growth, body composition and physical function compared to siblings and community controls, which are all indicators of

For peer review only

future cardiovascular and metabolic disease[12].

While anthropometry is used to assess nutritional recovery after discharge from hospital, the pattern and quality of growth recovery following SAM is poorly understood. The observation that children treated for SAM have a deficit in lean tissue despite regaining weight suggests that assessing body composition in addition to anthropometry may help to identify children who have not completely recovered and are at potential risk of long-term metabolic diseases[12]. Children with HIV-SAM appear to have potential for catch-up growth in weight-for-age and/or weight-for-height, which have been shown to normalise with treatment even prior to widespread availability of ART[16]; by contrast, height-for-age shows less potential for catch-up growth[17]. However, the body composition of children with HIV-SAM compared to HIV-negative children with SAM has not been described. Whether children recover fat mass at the expense of lean mass is unknown, but differences in tissue accretion patterns may have implications for survival and long-term metabolic health[18 19]. There is also a need to consider the effect of SAM on the size of body parts which grow at different rates: relatively shorter legs, for example, are associated with epidemiologic risk of overweight, coronary artery disease, liver dysfunction and diabetes[20 21].

Taken together, there is clearly an elevated risk of mortality among HIV-positive children with SAM compared to HIV-negative children with SAM, and an ongoing mortality risk among all children with SAM that persists after discharge from hospital. There are several gaps in our understanding of the long-term outcomes: (i) causes of death have not been clearly defined; (ii) no studies have systematically and longitudinally collected morbidity and mortality data or documented repeat hospitalisations post-discharge; and, (iii) the long-term outcomes of HIV-positive children with SAM in the era of ART availability are unclear.

For peer review only - http://bmjopen.bmj.com/site/about/guidelines.xhtml

Pathogenesis of SAM

Better understanding the pathogenesis of SAM may help to explain the high mortality of children both during and after hospitalisation and identify new targets for interventions to supplement existing treatment strategies. Consistent evidence that immune mediators are altered in malnutrition[22] and that systemic and intestinal inflammation are associated with poor outcomes in SAM[23], suggest that immune dysfunction contributes to infectious susceptibility[24]. Malnutrition is also characterised by a complex derangement in gut microbial[25] metabolic,[26] immune[27] and hormonal pathways, organ dysfunction and micronutrient deficiencies in the context of co-infections, enteropathy and chronic inflammation. Several studies have recently provided insights into these perturbations using new tools[25 26 28 29], including metabolomics and metagenomics, but we still lack a clear understanding of many of the pathogenic pathways driving malnutrition, the interactions between these pathways, and which are the most tractable targets for intervention.

SAM shares several pathological and clinical features with HIV, which may explain clinical outcomes in these co-occuring conditions: 1) both are characterised by intestinal damage, leading to impairment of the mucosal barrier and increased intestinal permeability; 2) both have underlying systemic immune activation; and 3) both are frequently complicated by persistent diarrhoea, pneumonia and sepsis that may plausibly arise due to loss of intestinal barrier function[30]. Understanding the overlapping impact of HIV and SAM is critical to inform additional interventions to improve outcomes of children with HIV-SAM.

OBJECTIVES OF HOPE-SAM

The Health Outcomes, Pathogenesis and Epidemiology of Severe Acute Malnutrition (HOPE-SAM) study has two primary objectives:

For peer review only - http://bmjopen.bmj.com/site/about/guidelines.xhtml

1) To describe the short- and long-term clinical outcomes of children with complicated SAM, with and without HIV infection, and to identify the risk factors at admission and discharge from hospital that independently predict these outcomes.
2) To better characterise the pathogenesis of SAM through nested laboratory sub-studies evaluating enteropathy, gut microbiota, metabolomics and immune cell function.

STUDY DESIGN

HOPE-SAM is a longitudinal observational cohort study, enrolling between 600-800 children aged 0-59 months admitted with complicated SAM to the tertiary pediatric wards at two sites in Zimbabwe (Parirenyatwa Hospital and Harare Children's Hospital) and one in Zambia (University Teaching Hospital, Lusaka). Both HIV-positive and HIV-negative children will be enrolled. Throughout this paper, 'SAM' refers to all children, regardless of HIV status; where analyses specifically compare children by HIV status, groups are identified as HIV-positive children with SAM (or HIV-SAM) and HIV-negative children with SAM. All participants with SAM are followed for 48 weeks post-discharge, with longitudinal data collection and blood sampling. The study contains four nested sub-studies as shown in **Figure 1.** A subgroup of children will be recruited to the enteropathy substudy for which they will have the same follow-up procedures but more intensive biological specimen collection including stool (all time-points), urine after lactulose-mannitol (LM) challenge as an assessment of intestinal permeability, and nasogastric aspirate (baseline only); these children are also included in microbiota and metabolomics substudies. Children with SAM for whom blood samples are available are included in the immunology substudy, for which circulating inflammatory mediators will be assayed; functional cellular immunology assays will be conducted for all children in the immunology sub-study with sufficient sample volume (> 2mL) recruited after June

For peer review only

2017. A group of healthy children recruited from the same hospitals and communities, who are well-nourished and matched to children in the enteropathy substudy by age and HIV status, will have data and specimens collected to provide normative data for the laboratory substudies; these well-nourished controls will not be followed longitudinally.

The study protocol, data collection forms and standard operating procedures are available online at osf.io/29uaw.

RECRUITMENT

Screening: Caregivers of all hospitalised children are sensitised about the study. All new admissions aged 0-59 months are screened for SAM, which is defined according to WHO criteria as any of: weight-for-height Z-score (WHZ) <-3, MUAC <115 mm (if aged 6-59mo) and/or bilateral pitting oedema. All children with SAM are recruited from hospital and this study therefore focuses on complicated SAM; children with uncomplicated SAM will not be enrolled.

Eligibility for observational cohort: All children with SAM whose caregivers are willing to provide written informed consent and to learn their child's HIV status are offered enrolment. Any children who die prior to study enrolment and those with a known malignancy are ineligible.

Eligibility for enteropathy substudy: Children with SAM aged 6-59 months with a nasogastric tube in place (or due to be placed) are categorized into 4 groups (HIV-positive oedematous (Group A-I); HIV-positive non-oedematous (Group A-II); HIV-negative oedematous (Group C-I) and HIV-negative non-oedematous (Group C-II), as shown in Table 1. Children meeting eligibility criteria will be enrolled throughout the study recruitment period until sufficient specimens have been collected from the

For peer review only

groups shown in Table 1. Children in the enteropathy substudy are stratified into age bands (6-11 months; 12-23 months and 24-59 months) to enable age-matching of well-nourished controls. Children with underlying chronic gastrointestinal disease or a known malignancy are ineligible.

Table 1: Enteropathy substudy groups

**Children aged 6-59 months**	**Severe acute malnutrition[1]**		**Well nourished controls** WHZ>-1
	**Oedematous[2]**	**Non-oedematous**	
**HIV-positive** (HIV PCR+ if <18mo; HIV antibody + if >18mo)	N=50 (Group A-I)	N=50 (Group A-II)	N=100[3] (Group B)
**HIV-negative** (HIV PCR- if <18mo; HIV antibody - if >18mo)	N=50 (Group C-I)	N=50 (Group C-II)	N=100[4] (Group D)

[1]SAM defined according to WHO criteria
[2]Presence of bilateral pitting pedal oedema.
Note that children below 6 months of age are excluded from the enteropathy substudy to avoid interrupting exclusive breastfeeding during the lactulose-mannitol test.
WHZ: Weight-for-height Z score; PCR: polymerase chain reaction.

Eligibility for microbiota and metabolomics substudies: Children enrolled into the enteropathy substudy are also included in the microbiota and metabolomics substudies, since these substudies utilize the stool, urine and plasma samples collected for enteropathy analyses.

Eligibility for immunology substudy: The immunology substudy comprises all children with SAM (drawn from both the observational cohort and the enteropathy substudy, as shown in Figure 1) providing a blood sample of sufficient volume (>2ml) for cellular assays after 1st June 2017.

Well-nourished controls: Controls are children drawn from the same hospitals and communities as cases with SAM (including well-nourished sibling controls), who are

For peer review only

aged 6-59 months (matched to enteropathy substudy children within age bands), well-nourished (weight-for-height Z-score >-1) and clinically well (no acute illness or current infections) with known HIV status. Controls are categorized into two groups: well-nourished HIV-positive (Group B) and well-nourished HIV-negative (Group D), as shown in Table 1. Children with underlying chronic gastrointestinal disease or a known malignancy are ineligible. Well-nourished controls provide comparison biomarker data for all the laboratory substudies.

Informed consent procedures: Written informed consent is obtained from the primary caregiver using consent forms translated into local languages; where possible, other family members are included in the consent process. Illiterate caregivers who have understood a verbal explanation of the study can provide a thumb imprint in the presence of a witness. Assent from children is not sought because all are <5 years old.

STUDY PROCEDURES

Study procedures are outlined in **Table 2**.

Table 2: Summary of procedures in observational cohort

Assessment	Hospitalization		Post-discharge[3]				
	Baseline[1]	Discharge[2]	2w	4w	12w	24w	48w
Caregiver informed consent to join observational cohort	x						
Summary checklist	x						
Locator information[4]	x						
Acute admission information	x						
Baseline data	x						
Daily clinical review[5]	Daily during hospitalisation						
Blood collection[6]	x	x			x	x	x
HIV testing[7]	x						
CD4 count and viral load	x				x	x	x

For peer review only

(HIV-infected children only)							
Full blood count[8]	x	x			x	x	x
Anthropometry	x	x	x	x	x	x	x
Skinfold thickness[9]		x	x	x	x	x	x
Body composition[10]	x	x	x	x	x	x	x
Discharge data collection		x					
Daily morbidity diary			Daily during follow-up period by caregivers				
Follow-up clinic: history, examination, morbidity and mortality data			x	x	x	x	x

[1]Children will be enrolled as soon as possible after hospitalisation and will undergo baseline investigations as soon as possible after enrolment. This is to provide a window of opportunity to time collection of research specimens with clinical specimens, and to ensure that the child is clinically stable before undertaking research investigations.
[2]The discharge procedures will be undertaken on the day of discharge, or as close as possible to that date.
[3]Windows will be created around these post-discharge time-points to maximize follow-up for caregivers who miss visits or are unavailable, as follows: 2 weeks (1-3 weeks); 4 weeks (3-5 weeks); 12 weeks (10-14 weeks); 24 weeks (20-28 weeks); 48 weeks (44-52 weeks).
[4]Locator information will updated at subsequent visits if caregivers have moved or changed contact details.
[5]A clinical review will be undertaken every day between admission and discharge by the study clinician**.**
[6]5.4 mL of blood (depending on child weight; amount will not exceed 2 mL/kg total over 2 week period) will be collected by a study nurse into endotoxin-free EDTA tubes. Samples will be used to store whole blood, PBMC and plasma for subsequent measurement of CRP and albumin. Where blood sample volumes allow (≥2mL sample), bacterial binding assays and whole blood stimulations will be conducted and culture supernatants and cells stored for subsequent assessment of immune cell function at each time-point.
Study blood samples will not be collected from children with known haemoglobin <6 g/dL.
[7]HIV testing is conducted as part of routine clinical practice, but if it has not been undertaken, the study sample will be used to test for HIV, as stated in the informed consent form, since HIV status is required to allocate children to study groups.
[8]Full blood count results will be transcribed from clinical records; if not done by clinical teams, the EDTA sample will be used to measure FBC in clinical laboratories at each site
[9]Skinfold thickness (triceps, subscapular, supra-iliac) and mid-thigh circumference will be measured using Holtain calipers or tape measure.
[10]Body composition will be assessed by bioimpedance vector analysis.

Baseline procedures:

Baseline data on maternal and household characteristics, the child's past medical history and current illness are collected by a study nurse. Anthropometry, including body composition measured by whole-body (wrist-ankle) bio-electrical impedance analysis (BodyStat 1500MD; BodyStat Ltd., Douglas, Isle of Man), leg length using an electronic knemometer (Zimbabwe only) and triceps, subscapular and supra-iliac

For peer review only

skinfold thickness using calipers (Holtain Ltd., Crymych, UK) are undertaken at baseline. Blood (1mL/kg up to 5.4mL maximum) is collected at baseline into an endotoxin-free EDTA tube for all children and, in the enteropathy substudy, additionally into a PAXgene tube (PreAnalytiX GmbH, Hombrechtikon, Switzerland) for subsequent transcriptomic analysis. Blood is not collected from children with severe anaemia (known haemoglobin <6 g/dL). HIV testing is carried out in accordance with national guidelines as part of routine clinical practice; where it has not been done, the child's HIV status is ascertained using a rapid test antibody algorithm for children over 18 months, or HIV DNA PCR for children under 18 months. CD4 count/percentage and viral load are measured in HIV-positive children. Maternal HIV status is documented where available, so that HIV-exposed uninfected children can be identified. Blood samples are sent to research laboratories at each site to conduct whole blood stimulation and bacterial binding assays (as described in the immunology substudy) and to store aliquots of whole blood, peripheral blood cells and plasma at -80ºC[31]. In the enteropathy substudy, nasogastric aspirate, stool and urine (after an oral dose of lactulose and mannitol) are also collected. Lactulose and mannitol are ingested by the child after fasting and urine is collected over a two-hour period to measure recovery of lactulose and mannitol, a measure of intestinal absorptive capacity and permeability, as previously described[32].

Daily procedures: Routine inpatient management is undertaken by ward clinical teams according to local hospital protocols, which are based on WHO guidelines[2,33]. In addition, the HOPE-SAM study clinician at each hospital site collects daily data until discharge on clinical parameters (including daily examination), resolution of acute infections, nutritional recovery (loss of oedema, restoration of appetite, weight gain), and treatment/nutritional supplements received; this will allow us to evaluate differences in management between countries. Children with HIV-SAM who are ART-

For peer review only - http://bmjopen.bmj.com/site/about/guidelines.xhtml

naïve start ART according to national guidelines, which are based on WHO recommendations[2 34].

Discharge: The clinical team decides when the child is ready to be discharged, which is generally when their medical complications are resolving and the child has a good appetite and is clinically well and alert[2]. Children receive ready-to-use therapeutic feeds (RUTF) to take at home according to local guidelines. At discharge, the study nurse collects data and a repeat blood sample (including full blood count) and undertakes discharge anthropometry, body composition, leg length (Zimbabwe only) and triceps, subscapular and supra-iliac skinfold thickness measurements (**Table 2**). The caregiver is given a daily morbidity diary and pre-prepared stickers corresponding to different illnesses and shown how to complete the diary. The caregiver is provided with the date of the first follow-up appointment and contact details of the study nurse.

Follow-up: Children attend follow-up appointments at dedicated study clinics at 2, 4, 12, 24 and 48 weeks post-discharge. At each visit, the study physician undertakes a clinical assessment and the study nurse captures illness, medication and feeding data. Clinic data are transcribed from handheld medical records if available and the morbidity diary is reviewed and a new diary and stickers supplied. Anthropometry, body composition, leg length (Zimbabwe only) and triceps, subscapular and supra-iliac skinfold thicknesses are measured at each visit. Acute illnesses are treated in the study clinic, or the child is referred to hospital if necessary. Children with relapsed malnutrition are provided with nutritional supplements or RUTF according to local guidelines, or readmitted to hospital if they develop complicated SAM. Transport reimbursement for clinic attendance is provided to caregivers for each visit.

Blood is collected at weeks 12, 24 and 48 post-discharge into endotoxin-free EDTA

For peer review only

tubes to measure full blood count, CD4 count and viral load (HIV-positive children only), conduct whole blood stimulation and bacterial binding assays (where blood volumes >2mL), and store peripheral blood cells and plasma aliquots for subsequent analysis (all blood samples), including soluble and cellular markers of immune activation, as outlined in **Supplementary Table 1**. Children in the enteropathy substudy have additional stool and urine collection following lactulose-mannitol dosing as shown in **Table 3.**

Table 3: Summary of procedures for cases in the enteropathy substudy

Assessment	Hospitalization		Post-discharge[3]				
	Baseline[1]	Discharge[2]	2w	4w	12w	24w	48w
Caregiver informed consent to join observational cohort and enteropathy substudy	X						
Summary checklist	X						
Locator information[4]	X						
Acute admission information	x						
Baseline data	X						
Daily clinical review[5]	Daily during hospitalisation						
Blood collection[6]	x	x			x	x	x
HIV testing[7]	X						
CD4 count and viral load (HIV-infected children only)	X				x	x	x
Full blood count[8]	X	x			x	x	x
Gastric aspirate[9]	x						
Stool collection[10]	x	x			x	x	x
Lactulose-mannitol testing[11]	x	x			x		x
Anthropometry	X	x	x	x	x	x	x
Skinfold thickness[12]		x	x	x	x	x	x
Body composition[13]	X	x	x	x	x	x	x
Discharge data collection		x					

For peer review only - http://bmjopen.bmj.com/site/about/guidelines.xhtml

Daily morbidity diary			Daily during follow-up period by caregivers				
Follow-up clinic: history, examination, morbidity and mortality data			x	x	x	x	x

[1]Children will be enrolled within 24h of hospitalization and will undergo baseline investigations within 72h of hospitalization. This is to provide a window of opportunity to time collection of research specimens with clinical specimens, and to ensure that the child is clinically stable before undertaking research investigations.
[2]The discharge procedures will be undertaken on the day of discharge, or as close as possible to that date.
[3]Windows will be created around these post-discharge time-points to maximize follow-up for caregivers who miss visits or are unavailable, as follows: 2 weeks (1-3 weeks); 4 weeks (3-5 weeks); 12 weeks (10-14 weeks); 24 weeks (20-28 weeks); 48 weeks (44-52 weeks).
[4]Locator information will updated at subsequent visits if caregivers have moved or changed contact details.
[5]Daily clinical review will be conducted every day between admission and discharge by the study clinician.
[6]During hospitalisation, 5.4 mL of blood (depending on child weight; amount will not exceed 2 mL/kg total over 2 week period) will be collected by a study nurse into a 2.7 mL endotoxin-free EDTA tube and a 2.7 mL PAXGene tube, for subsequent isolation of RNA and gene expression analysis. After discharge (weeks 12, 24 and 48), 5.4 mL of blood (depending on child weight; amount will not exceed 2 mL/kg total over 2 week period) will be collected by a study nurse into two 2.7 mL endotoxin-free EDTA tubes.
[7]HIV testing is conducted as part of routine clinical practice, but if it has not been undertaken, the study sample will be used to test for HIV (see section 9.4), as stated in the informed consent form, since HIV status is required to allocate children to study groups.
[8]Full blood count results will be transcribed from clinical records; if not done by clinical teams, the EDTA sample will be used to measure FBC in clinical laboratories at each site
[9]A gastric juice sample will be collected at the same time as the blood draw by aspirating the nasogastric tube with a sterile feeding syringe, to test for gastric pH; sterile water or saline will then be instilled and a sample of gastric juice collected for storage for subsequent PCR and culture (section 7.5.2)
[10]Stool collection will be undertaken at the same time as the blood draw
[11]Lactulose-mannitol testing will be conducted, with collection of a baseline urine sample, followed by a 2hr urine collection post-LM ingestion. This test will be deferred until children are judged to be clinically stable by the study physician during daily reviews. In general, this will be a child in the nutritional rehabilitation phase, who has no cardiorespiratory compromise.
[12]Skinfold thickness (triceps, subscapular, supra-iliac) and mid-thigh circumference will be measured using Holtain calipers or tape measure.
[13]Body composition will be assessed by bioimpedance vector analysis.

Caregivers are reminded of follow-up visits by phone, and visit completion is tracked on a dedicated database. If caregivers do not attend follow-up appointments, attempts are made to contact them by phone and home visits are made if feasible, particularly for those defaulting the 48-week visit, so that long-term outcome data can be collected. For post-discharge deaths, a home visit is undertaken by study nurses where possible to conduct a verbal autopsy. Children who are readmitted to one of

For peer review only - http://bmjopen.bmj.com/site/about/guidelines.xhtml

the study sites with relapsed SAM have data collected during the new episode of hospitalisation. The study ends for each participant at the week 48 visit.

SUBSTUDIES

As outlined in Figure 1, four nested substudies will utilise biological specimens to address mechanistic questions related to enteropathy, microbiota, metabolomics and immune function.

Enteropathy substudy

The gut, which acts as an internal interface between humans and the environment, must contain the nutrient stream and the symbiotic microbiota while allowing molecular intimacy to permit absorption. The mechanism underlying this duality is the integrity of the gastrointestinal barrier; intestinal damage (enteropathy) can impair this critical barrier function. A spectrum of enteropathies affect children in developing countries[30]. Environmental enteric dysfunction (EED), characterised by small intestinal inflammation, blunted villi and increased intestinal permeability, is almost universal and is morphologically indistinguishable from HIV enteropathy[30]. Children in resource-poor settings also suffer from frequent diarrhoea, food insecurity and micronutrient deficiencies, which all exacerbate enteropathy[30]. As a result, a cycle of intestinal infection, impaired mucosal function and malnutrition commonly arises, which may ultimately precipitate SAM, especially in the context of HIV infection[35 36]. It is not yet established if the enteropathy seen in children with SAM[37], which we here refer to as malnutrition enteropathy[37], is qualitatively or quantitatively distinguishable from EED. In addition to local intestinal pathology, enteropathies may cause systemic pathology due to persistent immune activation arising from enteric inflammation and microbial translocation across the damaged gut wall[30]. It is becoming apparent that chronic inflammation may be particularly deleterious in malnourished individuals[23]; in

For peer review only - http://bmjopen.bmj.com/site/about/guidelines.xhtml

children with SAM, systemic inflammation arising from underlying enteropathy may further increase morbidity and mortality.

We hypothesize that i) the degree of enteropathy during hospitalisation differs between oedematous and non-oedematous SAM and is independently associated with morbidity, mortality and nutritional recovery during hospitalization; ii) the degree of enteropathy at discharge is independently associated with morbidity, mortality and relapse of SAM; and iii) children with HIV-SAM have more severe enteropathy than HIV-negative children with SAM, which contributes to their poorer outcomes.

Using stored samples, a longitudinal series of investigations will compare gastric and small intestinal barrier function, using a range of biomarkers to capture the domains of malnutrition enteropathy (**Supplementary Table 2**). To understand better the extra-intestinal consequences of enteropathy, we will first compare the microbial composition of the upper gut and plasma using deep sequencing in a subgroup of children with paired gastric and blood samples. Secondly, we will undertake transcriptomics using PAXGene blood samples to determine i) whether there are differences in gene expression profiles between well-nourished controls, HIV-negative children with SAM and HIV-positive children with SAM (including comparison of oedematous and non-oedematous types); and ii) whether specific patterns of gene expression are associated with morbidity and mortality in SAM.

Microbiota substudy

Normal assembly of the gut microbiota in early life is critical for many aspects of physiological, neurological and immune development[38]. Recent evidence suggests that an immature or pathogenic microbiota plays a causative role in the pathogenesis of SAM[25]. For example, a number of microbial taxa have been identified, including *Faecalibacteium prausnitzii*, which discriminate and predict gut microbiota maturity

For peer review only - http://bmjopen.bmj.com/site/about/guidelines.xhtml

and child growth[28]. Other pathogenic microorganisms, including IgA-targeted *Enterobacteriaceae*, are associated with impaired growth and may contribute to SAM[39]. Nutritional rehabilitation with RUTF induces temporary recovery of a disturbed microbiota; however, the microbiota appears to revert back to an immature diseased state following nutritional recovery[29]. HIV infection is also associated with a disturbed gut microbiota[40], which may further compound enteropathy phenotypes. Furthermore, there is some evidence that differences exist in malnutrition enteropathy between oedematous and non-oedematous SAM[41]; however, few studies have investigated differences in the gut microbiota between the two forms of the disease.

We hypothesize that (i) a unique gut microbial signature exists in HIV-SAM, compared with HIV-negative children with SAM, that is independently associated with morbidity, mortality, nutritional recovery and degree of enteropathy during hospitalisation; (ii) a unique gut microbial signature exists in oedematous compared with non-oedematous SAM; (iii) specific microorganisms or gut microbial diversity indices are independently associated with morbidity, mortality, nutritional recovery and degree of enteropathy during hospitalisation; and (iv) the gut microbiota is partially restored to a healthy state with nutritional rehabilitation but reverts to a dysbiotic state during follow-up, which predicts morbidity, mortality and relapse of SAM.

Using stored stool samples collected at baseline, a cross-sectional investigation will determine differences in the gut microbial composition and predicted function between: HIV-negative children with SAM versus HIV-SAM, oedematous versus non-oedematous SAM, and well-nourished controls. Gut microbial composition and predicted function will be compared between groups at discharge and at 12, 24 and 48 weeks post-discharge. Briefly, total DNA and/or RNA will be extracted from stool

For peer review only

samples and used as template for next-generation sequencing library preparation and for quantitative polymerase chain reaction (qPCR). Whole metagenome shotgun sequencing will be performed using the HiSeq 2500 system. Raw metagenomic sequencing data will be quality-filtered and analysed through a well-validated bioinformatics pipeline using MetaPhlAn[42] and HUMAnN[43]. The compositional and predicted functional metagenomic data generated will be used to identify signatures of SAM and to investigate associative links between specific gut microbial signatures and clinical outcomes.

Metabolomics substudy

During SAM, metabolic processes are altered in response to a starved environment, and may plausibly contribute to long-term clinical outcomes. Previous studies suggest that amino acid turnover, lipid metabolism, oxidative stress and other metabolic pathways are disrupted in SAM and may be associated with disease state and clinical outcome[26 44 45]; however, little is known about how the metabolic phenotype responds to nutritional therapy. It is hypothesised that disturbed gut microbiota composition and function may drive microbial metabolic dysregulation in addition to host-derived dysregulation. Of particular interest are differences in the metabolic phenotype between oedematous and non-oedematous SAM. The 'reductive adaptation' seen in non-oedematous SAM (utilisation of fat and muscle stores) is disrupted in oedematous SAM, which may contribute to differences in clinical outcomes. Specifically, protein turnover, inflammation, oxidative stress and bile acid metabolism are disrupted in oedematous-SAM, which may contribute to co-morbidities including diarrhoea, steatosis and enteropathy[46 47].

We hypothesize that: (i) a unique plasma and urine metabolic phenotype exists in oedematous compared with non-oedematous SAM during hospitalisation, which is

For peer review only - http://bmjopen.bmj.com/site/about/guidelines.xhtml

independently associated with morbidity, mortality and nutritional recovery; (ii) the metabolic phenotype is partially restored to a healthy state with nutritional rehabilitation but reverts to a disturbed state during follow-up, which predicts morbidity, mortality and relapse; and (iii) both host-derived and gut microbial-driven metabolic dysregulation underlie clinical outcomes.

Using stored urine and plasma samples collected during hospitalisation, a cross-sectional investigation will determine differences in the metabolic phenotype between children with oedematous SAM, non-oedematous SAM and well-nourished controls. Urine and plasma metabolic phenotypes will be compared between groups at discharge and 12, 24 and 48 weeks post-discharge. Briefly, global untargeted metabolomic phenotyping will be performed via ^{1}H nuclear magnetic resonance (NMR) spectroscopy using a 700 MHz Bruker NMR spectrometer to identify metabolic signatures of SAM. Targeted analysis via ultra-performance liquid chromatography-mass spectrometry will be performed to examine specific pathways of interest, including tryptophan and bile acid metabolism.

Immunology substudy

Bacterial infections are common among children hospitalised for SAM[23 48-50] and mortality is driven by a range of species[48 50-53], consistent with generalised defects in innate anti-bacterial defence. Increased infectious morbidity and mortality persist after discharge from hospital[10 17 54], suggesting that restoration of anti-bacterial immune responses may lag behind nutritional rehabilitation. A recent randomised trial in children with SAM confirmed that deaths following hospitalisation were predominantly due to bacterial infections but were not prevented by daily co-trimoxazole prophylaxis[17]. Collectively, these observations highlight that children remain vulnerable to infection despite current treatment approaches; targeting

For peer review only - http://bmjopen.bmj.com/site/about/guidelines.xhtml

persistent immune dysfunction could plausibly reduce infectious mortality after discharge[24].

Multiple innate and adaptive immune mediators are dysregulated in malnutrition[24 27 55]. However, few studies have assessed cellular immune function in malnourished children; most existing studies were undertaken decades ago on small cross-sectional cohorts without the benefit of recent advances in immunology techniques[27]. Immune dysfunction in SAM likely reflects both *intrinsic* defects, whereby immune cells lack capacity to adequately respond to infection, and *extrinsic* defects, where cells have intact anti-bacterial capacity but are chronically modulated by the systemic pro-inflammatory environment which characterises SAM (i.e. heightened pro-inflammatory cytokines[44] and circulating bacterial antigens[23 56 57]). Systemic inflammation is directly associated with mortality in SAM[23] and driven by multiple comorbidities, including bacterial translocation from the damaged gut into the blood, sub-clinical infections and metabolic dysregulation[44 58 59]. The implications of innate immune cell dysfunction for subsequent acquisition of infections and infectious mortality have not been investigated.

We hypothesise that: (i) anti-bacterial functions of innate immune cells are compromised in SAM due to a combination of intrinsic and extrinsic defects; ii) innate immune cell function is independently associated with infectious morbidity and mortality during hospitalisation for SAM; and iii) nutritional rehabilitation only partly restores innate immune cell function, leading to an ongoing risk of bacterial infections post-discharge.

Using blood samples collected at baseline, discharge and 12, 24 and 48 weeks post-discharge, the longitudinal relationship between circulating innate immune cell function and bacterial infections will be assessed. The intrinsic phagocytic capacity,

For peer review only

secreted cytokine response and maturation state of innate immune cells after culture with bacterial antigens will be assessed. Plasma concentrations of endotoxin and pro-inflammatory mediators will be quantified at each time-point and the degree to which these extrinsic factors influence innate immune cell antibacterial function will be assessed via plasma co-culture with innate immune cells from healthy donors. Bacterial infections during hospitalisation will be diagnosed using clinical criteria and blood culture, stool culture and urinalysis where available.

SAMPLE SIZES

Observational study: The observational cohort will recruit as many children with SAM as possible during the period of enrolment (July 2016 to March 2018), estimated at 600-800 children (capped at 800 maximum), to assess clinical and nutritional outcomes among HIV-positive and HIV-negative children hospitalised with SAM. Assuming mortality of 15%, overall loss to follow-up of 15% and recruitment target of 800 children, there would be 560 evaluable children at 48 weeks, of whom 224 would have HIV-SAM based on an estimated inpatient HIV prevalence of 40%. This will provide >80% power to detect absolute differences of 17% in binary outcomes between HIV-SAM and HIV-negative children with SAM, and of 0.33 times the standard deviation in continuous outcomes.

Enteropathy substudy: The sample size was estimated using previously reported values for LM ratios, which remain a widely used non-invasive marker of enteropathy. Comparing 100 versus 100 children with two-sided alpha=0.025 (to allow for two primary comparisons, i.e. HIV-SAM versus HIV-negative children with SAM, and HIV-SAM versus well-nourished HIV-positive children) provides >80% power to detect differences in mean LM ratio during hospitalisation of at least 0.16

For peer review only - http://bmjopen.bmj.com/site/about/guidelines.xhtml

(assuming SD=0.36), a difference which would be clinically relevant given the LM ratios previously reported for well-nourished children (0.42), malnourished children (1.3) and children with persistent diarrhoea (2.85) in the Gambia[60]. It also provides >80% power to detect differences of at least 0.1 in the mean change in LM ratio from enrolment (assuming SD for change=0.23 and 7% missing samples). For inflammatory markers, comparing 100 versus 100 children with two-sided alpha=0.025 provides >80% power to detect differences in mean $\log_{10}$ concentrations of at least 0.44 times their standard deviation, or 2.75-fold differences between groups. Inclusion of well-nourished controls provides an indication of normal ranges in young African children. HIV-positive and HIV-negative SAM groups will be stratified to include approximately 50 children with and without oedematous malnutrition, if possible.

Microbiota and metabolomics substudy

Power calculations are difficult in metagenomics and metabolomic analyses due to the large number of observed outcomes and unknown effect sizes and variance. Previous studies using smaller sample sizes have identified significant taxonomic differences in twin pairs discordant for oedematous-SAM (n=13)[25] and metabolic differences between the two forms of SAM (n=40)[26]. These studies suggest that a difference of 50% in metabolites could be expected. Using ANCOVA, setting $\alpha=0.05$ and assuming either low ($\rho=0.1$) or high ($\rho=0.7$) correlation, the study would require 95-126 subjects to achieve 80% power[61]. False discovery rate (FDR) multiple correction testing will be applied to reduce the high-dimensionality of the data and limit false-positives.

Immunology substudy

Up to 200 children with SAM and 200 well-nourished controls will be included in a cross-sectional analysis of innate immune cell function during hospitalisation.

For peer review only - http://bmjopen.bmj.com/site/about/guidelines.xhtml

Assuming similar infectious mortality to a recent Kenyan study (15%)[17], a cohort of 200 provides 80% power to detect associations between immune profiles and infectious mortality at an odds ratio of 1.7 and 2-sided alpha of 0.05. We will aim for 100 children with longitudinal analysis of innate immune cell function at discharge, 12, 24 and 48 weeks post-discharge[17].

STUDY OUTCOMES AND RISK FACTORS

The main study outcomes are clinical (mortality, morbidity and relapse of malnutrition) and nutritional (weight, height, mid-upper arm circumference, leg length, head circumference, mid-thigh circumference, skin-fold thickness and body composition by bioimpedance vector analysis) assessed over 48 weeks of follow-up. Mortality is assessed in hospital by daily physician review and, post-discharge, through study visits and by telephone where possible for children who are lost to follow-up. Morbidity during hospitalization is assessed through daily clinical assessments and available hospital laboratory tests. Morbidity after discharge is assessed, first, using daily morbidity diaries, in which caregivers record episodes of illness (lethargy interfering with feeding; respiratory distress; diarrhoea; oedema and fever); second, from caregiver recall and review of handheld medical records at each follow-up visit; and, third, from data collected during hospitalization for children who are readmitted during the follow-up period. Time-to-recovery from malnutrition will be evaluated during hospitalization; relapse of malnutrition during follow-up will be categorized as moderate acute malnutrition, uncomplicated SAM and complicated SAM, according to WHO definitions. Nutritional outcomes will be expressed both as continuous variables (attained Z-score and change in Z-score between visits), and as categorical variables (moderate wasting, WHZ<-2; severe wasting, WHZ<-3; stunting, HAZ<-2; severe stunting, HAZ<-3; underweight, WAZ <-2; and microcephaly, head circumference-for-age <-2).

For peer review only - http://bmjopen.bmj.com/site/about/guidelines.xhtml

Risk factors will be evaluated at baseline, hospital discharge and over the period of follow-up for associations with clinical and nutritional outcomes. In addition to baseline clinical and demographic factors, the following laboratory parameters will be evaluated: haemoglobin, serum albumin, C-reactive protein, CD4 count and HIV viral load (for HIV-positive children). Haemoglobin, CD4 and HIV-viral load will be measured in real time and the results reviewed during follow-up clinics.

Data on potential confounders are collected at baseline, discharge and during the follow-up period, including child feeding practices, household socioeconomic status (defined by household income and cooking method), maternal employment and education, and household factors such as water, sanitation and hygiene practices, availability of electricity, location (rural, peri-urban or urban) and household size.

ANALYSIS

All analyses will be interpreted exploratively since HOPE-SAM is an observational study with multiple risk factors, outcomes and substudies. For all analyses, P values will not be artificially adjusted, but interpreted as exploring the strength of evidence supporting any association. The only exception is the use of approaches to minimise false discovery when analysing high-dimensional data from the microbiota and metabolomics substudies, as described.

Observational Cohort

The primary comparison will be the clinical and nutritional outcomes of children with HIV-SAM compared to HIV-negative children with SAM. We will review all deaths and adjudicate clinical diagnoses and causes of death to ensure robust and consistent data across sites. We will compare each participant's clinical management to WHO guidelines to identify any contributory factors in hospital care. Factors associated with outcomes during hospitalisation (e.g. mortality, nutritional recovery)

For peer review only

will be determined for each group (HIV-SAM and HIV-negative children with SAM) using multivariable analysis (Cox models for time-to-event data, linear models for continuous outcomes). Factors associated with outcomes over 48 weeks post-discharge (hospital re-admission, morbidity and mortality, relapse, anthropometry, body composition and response to ART) will be determined for each group (HIV-SAM and HIV-negative children with SAM) using multivariable analysis (Cox models for time-to-event data, linear models for continuous outcomes). HIV-positive children with SAM and HIV-negative children with SAM will be included in one model together with the risk factors, and interaction tests will be used to investigate whether associations between risk factors and outcomes differ between the two groups of children. We will evaluate the ability of mid-upper arm circumference (MUAC) at discharge to predict long-term outcomes using receiver-operator-characteristic (ROC) analysis, in the whole cohort and within the subgroups of HIV-SAM and HIV-negative children with SAM. We will then evaluate whether addition of other variables improves the predictive capacity of MUAC (using WHO criteria in those >6 months old, and published data for children <6 months[62]) for each group, including body composition, haemoglobin, albumin and CRP, plus CD4%, viral load and timing of ART initiation (HIV-SAM only). We will construct multivariable models and compare them with MUAC alone using the net-reclassification index.

Body composition analysis

Previous work in body composition by bio-electrical impedance in Ethiopian infants and children with SAM has shown that the conventional approach, predicting total body weight from height-adjusted impedance, fails due to confounding by oedema[63]. The same project validated an alternative approach, known as Bio-electrical Impedance Vector Analysis (BIVA), and described significant differences between each of three groups: healthy controls, oedematous-SAM and non-oedematous SAM. Vector analysis splits impedance into two height-adjusted components,

For peer review only - http://bmjopen.bmj.com/site/about/guidelines.xhtml

resistance and reactance, which are further linked through phase angle (PA). Variability in these components is associated with biochemical parameters[64]. These variables will be explored using graphical analysis, or transformed into age- and sex-adjusted Z-scores for statistical comparison, including longitudinal analyses. Higher phase angle indicates better nutritional status, while declining height-adjusted resistance over time indicates loss of oedema.

Enteropathy substudy

The primary comparison for the enteropathy substudy will be between HIV-positive children with SAM (group A) and HIV-negative children with SAM (group C), stratified by presence or absence of oedema. Control groups (B and D) are well-nourished children with or without HIV, to provide normative data for biomarkers and to evaluate the impact of SAM within each HIV group. Thus, biomarkers among HIV-positive children with SAM will first be compared to HIV-negative children with SAM (to evaluate the impact of HIV) and, second, to well-nourished HIV-positive children (to evaluate the impact of SAM). Biomarkers among HIV-negative children with SAM will be compared to well-nourished HIV-negative children. For each continuous outcome, simple descriptive analysis will be used to compare groups during hospitalisation using t-tests on appropriately transformed data. For any outcome with moderate ($p<0.05$) evidence of difference between either group a regression model will be constructed including groups A, B, C, D to directly test (using interactions) whether there is a synergistic effect of HIV-SAM versus HIV-negative SAM versus HIV alone versus neither. These models will also be used to explore whether there is any evidence for heterogeneity in effects between oedematous and non-oedematous SAM. Associations between enrolment factors (e.g. intestinal permeability and microbial translocation) will be explored using pairwise Spearman correlations and principal components analysis. Mean changes at the follow-up time-points in each group will be estimated, and groups compared (as above) using generalised

For peer review only - http://bmjopen.bmj.com/site/about/guidelines.xhtml

estimating equations. For outcomes that differ across SAM groups over time, multilevel models will be used to explore possible predictors from the other factors measured. Time to nutritional recovery will be compared using Kaplan-Meier and log-rank tests, and Cox models to adjust for baseline differences between groups.

Microbiota and metabolomics substudy

The primary comparison will be between HIV-negative children with oedematous and non-oedematous SAM, with a separate comparison between HIV-positive children with SAM and HIV-negative children with SAM. Analyses will examine: (i) differences in metagenomic/metabolomic variables between groups at each time-point; (ii) differences in metagenomic/metabolomic variables within groups over time; (iii) correlations between metagenomic and metabolomic variables; and (iv) correlations between metagenomic/metabolomic variables and clinical outcomes. A systematic analysis will be undertaken to reduce high-dimensional data, integrate the multi-omics datasets and minimise false discovery.

Compositional metagenomic data will be compared between groups for indices of alpha and beta diversity. Principal coordinate analysis and partial least squares discriminant analysis will be performed on metabolomics data to identify overall differences between groups. High-dimensional datasets will be reduced using random forest models to identify taxa, microbial gene families and metabolites that most strongly contribute to differences between groups, corrected by Benjamani-Hochburg false discovery rate detection. Targeted analysis by qRT-PCR will validate differential abundance or expression of candidate microbial genes. Longitudinal comparisons will be performed within and between groups using multilevel simultaneous component analysis. Orthogonal projections to latent structures models will integrate metabolomic and metagenomic data whilst linear regression, canonical correlation and hierarchal clustering analysis will measure correlations between -

For peer review only - http://bmjopen.bmj.com/site/about/guidelines.xhtml

omics datasets. Finally, ROC analysis will identify the ability of different analytes to predict long-term nutritional and clinical outcomes.

Immunology substudy

Integrated profiles of innate immune cell function will be generated for each child using principal components analysis followed by hierarchical clustering[65 66]. This data-reduction method identifies whether absolute levels of specific markers or relative differences between markers differentiate children into groups. The resulting innate immune profiles will be compared between HIV-SAM, HIV-negative children with SAM and well-nourished groups using univariable tests and multivariable analysis of variance (MANOVA) of the principal components.

To address the relationship between immune function and infections, regression analyses will determine whether baseline innate immune profiles (or the individual parameters defining them) are associated with the infectious morbidity or mortality during hospitalisation, using logistic models for binary outcomes and linear models for duration. Key clinical characteristics, including age, sex, oedema and baseline WHZ, will be added to models to investigate their confounding effects. Multivariable stacked regression methods will be used to compare the impact of different factors on severe bacterial infections based on heterogeneity tests.

To determine whether treatment for SAM restores innate immune cell antibacterial function, mixed effects regression models will compare longitudinal changes in individual immune parameters, and the principal components calculated from the weights identified at baseline (which include well-nourished controls). Similarities and differences in longitudinal immune profiles will be compared between groups using nonmetric multi-dimensional scaling[65 67 68]. This approach will group children according to their composite innate immune function, allowing the duration and

For peer review only - http://bmjopen.bmj.com/site/about/guidelines.xhtml

variability of immune restoration to be evaluated over the course of nutritional rehabilitation. Binary logistic regression will determine whether innate immune profiles at discharge are associated with morbidity or mortality during follow-up.

PATIENT AND PUBLIC INVOLVEMENT

Patients and their caregivers were not involved in the design of the study. During recruitment, all caregivers of children admitted to hospital were given information about the study; those whose children had severe acute malnutrition were approached to give written informed consent. A meeting to disseminate results of the study to participants and their caregivers will be held at the end of the study. An interactive game to engage caregivers in the science underlying malnutrition is being developed in collaboration with experts from the Centre of the Cell, a unique science education centre based at Queen Mary University of London (https://www.centreofthecell.org/).

SAFETY REPORTING

For all adverse events, the study team will assess expectedness and relatedness to study activities. Since this is an observational study without interventions, we anticipate that the risk is minimal; however, serious adverse events will be reported to local ethical review boards (Medical Research Council of Zimbabwe, and University of Zambia Biomedical Research Ethics Committee) and the study sponsor (Queen Mary University of London) according to their respective guidelines.

DATA COLLECTION AND MONITORING

Clinical and demographic data are recorded on paper case report forms. All data are checked for completeness and plausibility before data entry and problems flagged for resolution by the clinical team. All data are double-entered onto a dedicated

For peer review only

password-protected online study database, and any discrepancies resolved. Study participants are identified on electronic databases only by study numbers (assigned at enrolment); no personal identifiers are entered.

ETHICS AND DISSEMINATION

The study complies with the principles of the Declaration of Helsinki (2008) and is conducted in compliance with the principles of Good Clinical Practice (GCP) and local regulatory requirements in each country. Ethical approval was obtained from the University of Zambia Biomedical Research Ethics Committee, the Joint Research Ethics Committee of the University of Zimbabwe and the Medical Research Council of Zimbabwe. The ethical review board of the Sponsor, Queen Mary University of London, provided an advisory review of the study. Since this is an observational study, there is no Data and Safety Monitoring Board.

Results will be disseminated through conference abstracts and peer-reviewed publications and discussed with relevant policymakers and programmers. Study findings will be disseminated to families of participants at face-to-face meetings.

TIME FRAME AND STUDY STATUS

Enrolment into the study began in July 2016 and is expected to end in March 2018. All participants will be followed for 48 weeks, with an expected study completion date of March 2019.

DISCUSSION

HOPE-SAM aims to document the short- and long-term clinical and nutritional outcomes of HIV-positive and HIV-negative children with SAM, and to identify the factors at presentation and at discharge from hospital that independently predict these outcomes. Mechanistic substudies aim to evaluate the contribution of

For peer review only

enteropathy, microbiota, metabolome and innate immune cell function to these clinical outcomes. The prevalence of malnutrition in HIV-positive children is as high as 40% in some settings and the challenges of managing this population are well recognised[69]. The WHO protocol on management of SAM aims to reduce case fatality below 10%, but rates as high as 35% are still reported among HIV-positive children[5 70]. No studies have systematically and longitudinally collected morbidity data in HIV-SAM, or documented repeat hospitalisations and mortality after discharge from hospital, particularly in the current era where ART is available upon diagnosis. HOPE-SAM will provide a unique opportunity to enrol and follow a cohort of children managed for SAM in three large hospitals across two sub-Saharan African countries at several time-points over a one-year period. Nested longitudinal laboratory substudies aim to better characterise the pathogenesis of SAM in HIV-positive and HIV-negative children, to determine whether pathogenic processes are normalised during nutritional rehabilitation and follow-up, and to identify potential mechanistic pathways. Our ultimate goal is to utilise the findings generated in this study to inform new intervention approaches that can be evaluated in clinical trials to improve outcomes among children with SAM.

ACKNOWLEDGEMENTS

Members of the HOPE-SAM study team not listed in the author list: *Harare, Zimbabwe:* Virginia Sauramba, Adlight Dandadzi, Chipo Kureva, Johnson Mushonga, Eddington Mpofu, Washington Dune, Tafadzwa Chidhanguro, Sibongile Nkiwane, Sandra Rukobo, Margaret Govha, Patience Mashayanembwa, Leah Chidamba, Bernard Chasekwa, Joice Tome, Rachel Makasi, Wellington Murenjekekwa, Theodore Chidawanyika, Blessing Tsenesa, Stephen Moyo, Penias Nyamwino, Pururudzai Simango, Shepherd Seremwe, Lovemore Chingaoma and Sarudzai Kasaru. *Lusaka, Zambia*: Andreck Tembo, Mary Mpundu, Evelyn Nyendwa, Gwendolyn Nayame, Dalitso Tembo, Sophreen Mwaba, Esther Chilala, Lucy

For peer review only

Macwani, Tenzeni Dumba, Miyoba Chipunza, Lydia Kazhila, Temwaninge Gondwe, Dennis Phiri, Mpala Mwanza, Kanekwa Zyambo.

We thank Philippa Rambanepasi, Karen Gwanzura and Agatha Muyenga for financial management of the study; Zinah Sorefan and Daniela Azurunwa for study coordination at Queen Mary University of London; Professor Kim Michaelsen for the use of the electronic knemometers and advice on their use; and we remember the hard work of Edith Mukusho who sadly passed away during the HOPE-SAM study. We thank the staff and management and members of the Department of Paediatrics and Child Health at the three hospital sites who have made this study possible. We are indebted to the caregivers, families and children who are participating in the HOPE-SAM study.

For peer review only

AUTHOR CONTRIBUTIONS

Designed study: MB-D, BA, CDB, RCR, BM, KC, CK, KCh, DN, PC, NC, FM, JW, ARM, JS, ASW, KJN, PK, AJP

Sought funding: MB-D, BA, CDB, RCR, JHH, ARM, JS, ASW, KJN, PK, AJP

Undertaking study: BM, KC, CK, KCh, FM, DN, PC, NC, FM, IM, EB, KM, SM, TR

Study oversight: MB-D, BA, JHH, KJN, PK, AJP

Analysis: MB-D, BA, CDB, RCR, RN, JW, ARM, JS, ASW, KJN, PK, AJP

Wrote first draft of manuscript: MB-D, CDB, RCR, AJP

Critically revised manuscript: All

FUNDING

This work was supported by the Medical Research Council UK (MR/K012711/1), the Wellcome Trust (107634/Z/15/Z to MB-D; 206225/Z/17/Z to CDB, an award funded in partnership with the Royal Society; 206455/Z/17/Z to RCR; and 108065/Z/15/Z to AJP), and a Bio-Resource Grant from the Centre for Genomic Health within the Life Sciences Initiative at Queen Mary University of London.

COMPETING INTERESTS

None of the authors have any competing interests to declare.

For peer review only

References

1. Black R E, Victora C G, Walker S P, et al. Maternal and child undernutrition and overweight in low-income and middle-income countries . *Lancet* 2013;382:427–51.

2. World Health Organisation G. Guideline Updates on the management of severe acute malnutrition in infants and children: World Health Organisation, Geneva, 2013.

3. Lenters LM, Wazny K, Webb P, et al. Treatment of severe and moderate acute malnutrition in low- and middle-income settings: a systematic review, meta-analysis and Delphi process. *BMC Public Health* 2013;13 (Supp 3):S23.

4. Golden MHN. Oedematous malnutrition. *British Medical Bulletin* 1998;54(2):433-44.

5. Schofield C, A. A. Why have mortality rates for severe malnutrition remained so high? *Bull World Health Organ* 1996;74(2):223-29.

6. Munthali T, Jacobs C, Sitali L, et al. Mortality and morbidity patterns in under-five children with severe acute malnutrition (SAM) in Zambia: a five-year retrospective review of hospital-based records (2009–2013). *Archives of Public Health* 2015;73(23)

7. Preidis G A, McCollum E D, Mwansambo C, et al. Pneumonia and Malnutrition are Highly Predictive of Mortality among African Children Hospitalized with Human Immunodeficiency Virus Infection or Exposure in the Era of Antiretroviral Therapy. *Journal of Pediatrics* 2011;159(3):484-89.

8. World Health Organisation G. Global guidance on criteria and processes for validation: Elimination of Mother-to-Child transmission of HIV and Syphilis. Geneva: World Health Organization, 2017.

9. Evans C, Jones CE, Prendergast AJ. HIV-exposed, uninfected infants: new global challenges in the era of paediatric HIV elimination . *Lancet Infectious Diseases* 2016 doi: 10.1016/S1473 [published Online First: March 31,2016]

10. Heikens GT, Bunn J, Amadi A, et al. Case management of HIV-infected severely malnourished children: challenges in the area of highest prevalence. *Lancet* 2008;371:1305-7.

11. Kerac M, Bunn J, George Chagaluka G, et al. Follow-Up of Post-Discharge Growth and Mortality after Treatment for Severe Acute Malnutrition (FuSAM Study): A Prospective Cohort Study. *Plos One* 2014;9(6)

12. Lelijveld M, Seal A, Wells J C, et al. Chronic disease outcomes after severe acute malnutrition in Malawian children (ChroSAM): a cohort study. *Lancet Global Health* 2016;4:e654-62.

13. Moïsi JC, Gatakaa H, Berkley JA, et al. Excess child mortality after discharge from hospital in Kilifi, Kenya: a retrospective cohort analysis. *Bull World Health Organ* 2011;89:725-32A.

For peer review only - http://bmjopen.bmj.com/site/about/guidelines.xhtml

14. Wiens MO, Pawluk S, Kissoon N, et al. Pediatric Post-Discharge Mortality in Resource Poor Countries: A Systematic Review. *Plos One* 2013;8(6)
15. Ngari MM, Fegan G, Mwangome MK, et al. Mortality after Inpatient Treatment for Severe Pneumonia in Children: a Cohort Study. *Paediatric and Perinatal Epidemiology* 2017;31:233-42.
16. Fergusson P, Chinkhumba J, Grijalva-Eternod C, et al. Nutritional recovery in HIV-infected and HIVuninfected children with severe acute malnutrition. *Archives of Diseases in Children* 2009;94:512-16.
17. Berkley JA, Ngari M, Johnstone T, et al. Daily co-trimoxazole prophylaxis to prevent mortality in children with complicated severe acute malnutrition: a
multicentre, double-blind, randomised placebo-controlled trial. *Lancet Global Health* 2016;4(7):e464 - e73
18. DeBoer M D, Lima A A M, Oría R B, et al. Early childhood growth failure and the developmental origins of adult disease: Do enteric infections and malnutrition increase risk for the metabolic syndrome? *Nutrition Reviews* 2012 70(11):642-53.
19. Barouki R, Gluckman P D, Grandjean P, et al. Developmental origins of non-communicable disease: Implications for research and public health. *Environmental Health* 2012;11(42)
20. Davey Smith G, Greenwood R, Gunnell D, et al. Leg length, insulin resistance, and coronary heart disease risk: The Caerphilly Study. *Journal of Epidemiology Community Health* 2001;55:867-72.
21. Gunnell D, Whitley E, Upton MN, et al. Associations of height, leg length, and lung function with cardiovascular risk factors in the Midspan Family Study. *Journal of Epidemiology Community Health* 2002;57:141-46.
22. Jones K D, Thitiri J, Ngari M, et al. Childhood malnutrition: Toward an understanding of infections, inflammation, and antimicrobials. *Food and Nutrition Bulletin* 2014;35(2)
23. Attia S, Versloot CJ, Voskuijl W, et al. Mortality in children with complicated severe acute malnutrition is related to intestinal and systemic inflammation: an observational cohort study. *American Journal of Clinical Nutrition* 2016;104:1441-9.
24. Bourke CD, Berkley J A, Prendergast AJ. Immune Dysfunction as a Cause and Consequence of Malnutrition. *Trends in Immunology* 2016;37(6):386-89.
25. Smith MI, Yatsunenko T, Manary MJ, et al. Gut microbiomes of Malawian twin pairs discordant for kwashiorkor. *Science* 2013;339(6119):548-54.
26. Di Giovanni V, Bourdon C, Wang DX, et al. Metabolomic Changes in Serum of Children with Different Clinical Diagnoses of Malnutrition. *Journal of Nutrition* 2016;146:2436-44.
27. Rytter MJH, Kolte L, Briend A, et al. The Immune System in Children with Malnutrition—A Systematic Review. *Plos One* 2014;9(8)
28. Blanton LV, Charbonneau MR, Salih T, et al. Gut bacteria that prevent growth impairments transmitted by microbiota from malnourished children. *Science* 2016;351(6275)
29. Subramanian S, Huq S, Yatsunenko T, et al. Persistent gut microbiota immaturity in malnourished Bangladeshi children. *Nature* 2014;510(7505):417-21.

For peer review only - http://bmjopen.bmj.com/site/about/guidelines.xhtml

30. Prendergast A, Kelly P. Review: Enteropathies in the developing world: neglected effects on global health. *Am J Trop Med Hyg* 2012;86:756-63.
31. Lutwama F, Kagina BM, Wajja A, et al. Distinct T-Cell Responses When BCG Vaccination Is Delayed From Birth to 6 Weeks of Age in Ugandan Infants. *The Journal of Infectious Diseases* 2014;209(6):887-97.
32. Prendergast AJ, Humphrey JH, Mutasa K, et al. Assessment of Environmental Enteric Dysfunction in the SHINE Trial: Methods and Challenges. *Clin Infect Dis* 2015;61 (suppl 7):S685-702.
33. World Health Organisation G. Management of severe malnutrition : a manual for physicians and other senior health workers. 1999
34. World Health Organisation G. Consolidated guidleines on the use of antiretroviral drugs for treating and preventing HIV infection: Recommendations for a public health approach. 2016.
35. Costiniuk CT, JB. A. Human immunodeficiency virus and the gastrointestinal immune system: does highly active antiretroviral therapy restore gut immunity? *Nature* 2012;5(6):596-604.
36. Brenchley JM, Douek DC. HIV infection and the gastrointestinal immune system

. *Nature* 2008;1(1) doi: doi:10.1038/mi.2007.1
37. Amadi B, Besa E, Zyambo K, et al. Impaired Barrier Function and Autoantibody Generation in Malnutrition Enteropathy in Zambia. *EBioMedicine* 2017;22(Supplement C):191-99. doi: https://doi.org/10.1016/j.ebiom.2017.07.017
38. Kau AL, Ahern PP, Griffin NW, et al. Human nutrition, the gut microbiome and the immune system. *Nature* 2011;474(7351):327-36.
39. Kau AL, Planer JD, Liu J, et al. Functional characterization of IgA-targeted bacterial taxa from undernourished Malawian children that produce diet-dependent enteropathy. *Sci Transl Med* 2015;7(276):276ra24. doi: 10.1126/scitranslmed.aaa4877
40. Monaco CL, Gootenberg DB, Zhao G, et al. Altered Virome and Bacterial Microbiome in Human Immunodeficiency Virus-Associated Acquired Immunodeficiency Syndrome. *Cell Host Microbe* 2016;19(3):311-22. doi: 10.1016/j.chom.2016.02.011
41. Amadi B, Fagbemi AO, Kelly P, et al. Reduced production of sulfated glycosaminoglycans occurs in Zambian children with kwashiorkor but not marasmus. *Am J Clin Nutr* 2009;89(2):592-600. doi: 10.3945/ajcn.2008.27092
42. Segata N, Waldron L, Ballarini A, et al. Metagenomic microbial community profiling using unique clade-specific marker genes. *Nature methods* 2012;9(8):811-4. doi: 10.1038/nmeth.2066 [published Online First: 2012/06/13]
43. Abubucker S, Segata N, Goll J, et al. Metabolic reconstruction for metagenomic data and its application to the human microbiome. *PLoS computational biology* 2012;8(6):e1002358. doi: 10.1371/journal.pcbi.1002358 [published Online First: 2012/06/22]

For peer review only - http://bmjopen.bmj.com/site/about/guidelines.xhtml

44. Bartz S, Mody A, Hornik C, et al. Severe Acute Malnutrition in Childhood: Hormonal and Metabolic Status at Presentation, Response to Treatment and Predictors of Mortality. *Journal of Clinical and Endocrine Metabolism* 2014;99(6):2128-37.
45. Farras M, Chandwe K, Mayneris-Perxachs J, et al. Characterizing the metabolic phenotype of intestinal villus blunting in Zambian children with severe acute malnutrition and persistent diarrhoea. *Plos One* 2018;13(3) doi: doi.org/10.1371/journal.pone.0192092
46. Mayneris-Perxachs J, Lima AA, Guerrant RL, et al. Urinary N-methylnicotinamide and β-aminoisobutyric acid predict catch-up growth in undernourished Brazilian children. *Sci Rep* 2016;6:19780. doi: 10.1038/srep19780
47. Manary MJ, Leeuwenburgh C, Heinecke JW. Increased oxidative stress in kwashiorkor. *J Pediatr* 2000;137(3):421-4. doi: 10.1067/mpd.2000.107512
48. Chisti MJ, Salam MA, Bardhan PK. Severe Sepsis in Severely Malnourished Young Bangladeshi Children with Pneumonia: A Retrospective Case Control Study. *Plos One* 2015;10(10):e0139966.
49. Page A-L, de Rekeneire N, Sayadi S, et al. Infections in children admitted with complicated severe acute malnutrition in Niger. *Plos One* 2013;8(7)
50. Jones KDJ, A BJ. Severe acute malnutrition and infection. *Paediatrics and International Child Health* 2014;34(sup 1):S1-S29. doi: DOI: 10.1179/2046904714Z.000000000218
51. Bahwere P, Levy J, Philippe H, et al. Community-Acquired Bacteremia among Hospitalized Children in Rural Central Africa. *International Journal of Infectious Diseases* 2001;5:180-88.
52. Thuo N, Ohuma E, Karisa J, et al. The prognostic value of dipstick urinalysis in children admitted to hospital with severe malnutrition. *Archives of Diseases in Childhood* 2010;95(6):422-6.
53. Berkley J A, Lowe BS, Mwangi I, et al. Bacteremia among Children admitted to a rural Hospital in Kenya. *New England Journal of Medicine* 2005;352:39-47.
54. Chang CY, Trehan I, Wang R J, et al. Children Successfully Treated for Moderate Acute Malnutrition Remain at Risk for Malnutrition and Death in the Subsequent Year after Recovery. *Journal of Nutrition* 2013;143:215-20.
55. Ibrahim MK, Zambruni M, Melby CL, et al. Impact of Childhood Malnutrition on Host Defense and Infection. *Clinical Microbiology Reviews* 2017;30(4):919-71.
56. Hughes SM, Amadi B, Mwiya M, et al. Dendritic cell anergy results from endotoxemia in severe malnutrition. *Journal of Immunology* 2009;183(4):2818-26.
57. Jones K. D, Hünten-Kirsch B, Laving AM, et al. Mesalazine in the initial management of severely acutely malnourished children with environmental enteric dysfunction: a pilot randomized controlled trial. *BMC Med* 2014;12 doi: 10.1186/s12916-014-0133-2
58. Watanabe K, A. PJW. Environmental Enteropathy: Elusive but Significant Subclinical Abnormalities in Developing Countries. *EBioMedicine* 2016;10:25-32.

For peer review only - http://bmjopen.bmj.com/site/about/guidelines.xhtml

59. Kelly P, Besa E, Zyambo K. Endomicroscopic and Transcriptomic Analysis of Impaired Barrier Function and Malabsorption in Environmental Enteropathy. . *Plos Neglected Tropical Diseases* 2016;10(4):e00046000.
60. Behrens RH, Lunn PG, Northrop CA, et al. Factors affecting the integrity of the intestinal mucosa of Gambian children. *American Journal of Clinical Nutrition* 1987;45:1433-41.
61. Borm GF, Fransen J, WA L. A simple sample size formula for analysis of covariance in randomized clinical trials. *Journal of Clinical Epidemiology* 2007;60(12):1234-8.
62. Mwangome M, Ngari M, Fegan G, et al. Diagnostic criteria for severe acute malnutrition among infants aged under 6 mo. *The American journal of clinical nutrition* 2017;105(6):1415-23. doi: 10.3945/ajcn.116.149815 [published Online First: 2017/04/21]
63. Girma T, Kæstel P, Workeneh N, et al. Bioimpedance index for measurement of total body water in severely malnourished children: Assessing the effect of nutritional oedema. *Clinical Nutrition* 2015
64. Girma T, Hother Nielsen AL, Kæstel P, et al. Biochemical and anthropometric correlates of bio-electrical impedance parameters in severely malnourished children: A cross-sectional study. . *Clinical Nutrition* 2017 doi: 10.1016/j.clnu.2017.02.017
65. Bourke CD, Nausch N, Rujeni N, et al. Integrated Analysis of Innate, Th1, Th2, Th17, and Regulatory Cytokines Identifies Changes in Immune Polarisation Following Treatment of Human Schistosomiasis. *The Journal of Infectious Diseases* 2013;208(1):159-69.
66. Prendergast AJ, Szubert AJ, Berejena C, et al. Baseline Inflammatory Biomarkers Identify Subgroups of HIV-Infected African Children With Differing Responses to Antiretroviral Therapy. *Journal of Infectious Diseases* 2016;214:226-36
.
67. Bourke CD, Mutapi F, Nausch N, et al. Trichuris suis ova therapy for allergic rhinitis does not affect allergen-specific cytokine responses despite a parasite-specific cytokine response. *Clinical and Experimental Allergy* 2012;42:1582-95.
68. Bourke CD, Nausch N, Rujeni N, et al. Cytokine Responses to the Anti-schistosome Vaccine Candidate Antigen Glutathione-S-transferase Vary with Host Age and Are Boosted by Praziquantel Treatment. *Plos Neglected Tropical Diseases* 2014;8(5)
69. Fergusson P, Tomkins A. HIV prevalence and mortality among children undergoing treatment for severe acute malnutrition in sub-Saharan Africa: a systematic review and meta-analysis. *Transactions of the Royal Society of Tropical Medicine and Hygiene* 2009;103(6):541-48.
70. Chinkhumba J, Tomkins A, Banda C, et al. The impact of HIV on mortality during in-patient rehabilitation of severely malnourished children in Malawi. *Transactions of the Royal Society of Tropical Medicine and Hygiene* 2008;102(7):639-44.

For peer review only

FIGURE LEGENDS

Figure 1: **Study flow chart.**

All hospital admissions are screened for eligibility for the observational cohort and enteropathy sub-study, with procedures undertaken as shown in the flow chart during hospitalisation and post-discharge. Well-nourished children from outpatient clinics and the community meeting eligibility criteria as well-nourished controls are enrolled and undergo a single baseline assessment as shown. The immunology, microbiota and metabolomics sub-studies enrol children as shown. All children with SAM, regardless of which arm of the study they are enrolled into, are followed for 48 weeks post-discharge.

[1]The immunology substudy started from 1st June 2017 and required children to have a blood sample >2mL to conduct cellular assays.

For peer review only

For peer review only - http://bmjopen.bmj.com/site/about/guidelines.xhtml

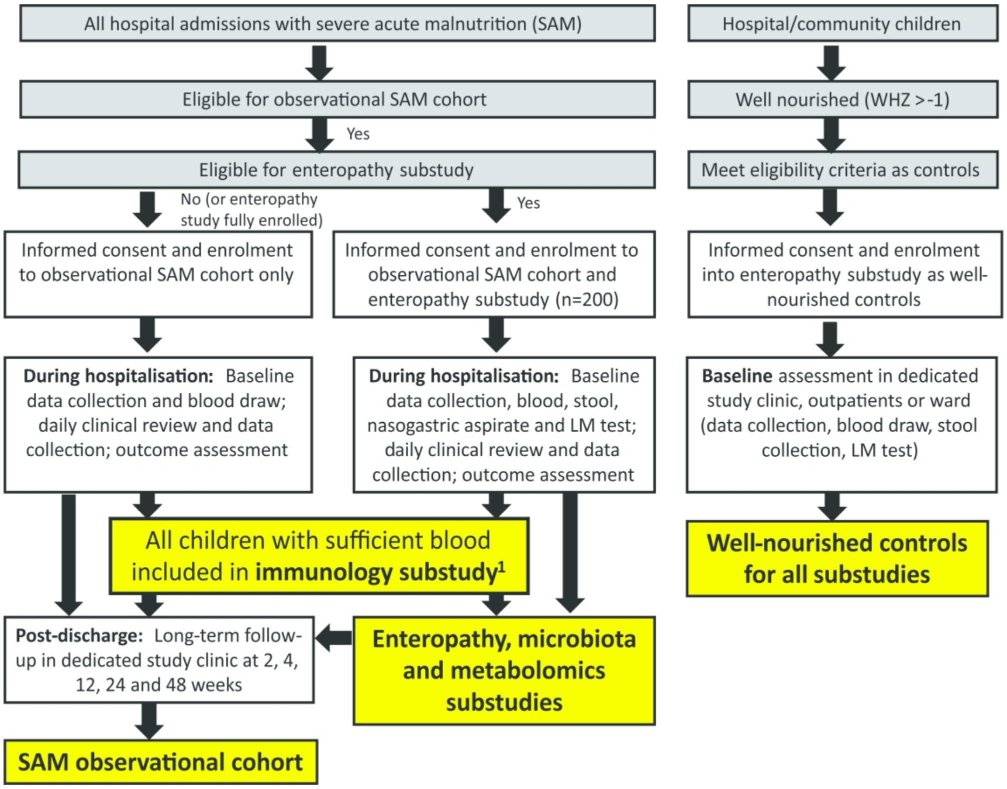

204x159mm (300 x 300 DPI)

Supplementary Table 1: Assays undertaken on stored samples for children in the observational cohort and immunology substudy.

Sample type	Assay (method)	Location of work	Study subjects	Baseline	Discharge	Week 12	Week 24	Week 48
Blood	HIV testing (rapid antibody test algorithm if >18 mo old or HIV DNA PCR <18 mo old)[1]	TROPGAN, Zvitambo or clinical sites	All	X				
Blood	CD4 count (flow cytometry or PIMA)[1]	TROPGAN, Zvitambo or clinical sites	All HIV-positive	X	X	X	X	X
Plasma	HIV viral load (real-time PCR)[1]	TROPGAN, Zvitambo or clinical sites	All HIV-positive	X	X	X	X	X
Plasma	C-reactive protein (ELISA)	TROPGAN, Zvitambo	All	X	X	X	X	X
Plasma	Albumin (ELISA)	TROPGAN, Zvitambo	All	X	X	X	X	X
Plasma	Lipopolysaccharide (LAL assay)	TROPGAN and Zvitambo	Subgroup[3]	X	X	X	X	X
Plasma	Lipopolysaccharide binding protein (LBP)	TROPGAN and Zvitambo	Subgroup[3]	X	X	X	X	X
Plasma	sCD14 (ELISA)	TROPGAN and Zvitambo	Subgroup[3]	X	X	X	X	X
Plasma	sCD163 (ELISA)	TROPGAN and Zvitambo	Subgroup[3]	X	X	X	X	X
Plasma	IL-6, TNF-alpha, IL-1β (ELISA) and/or multiplex cytokines	TROPGAN and Zvitambo	Subgroup[3]	X	X	X	X	X
Plasma	Total PAMP activity (THP1 reporter cell line[2])	TROPGAN and Zvitambo	Subgroup[3]	X	X	X	X	X
Whole	Molecular techniques	QMUL, London[2]	Subgroup[3]	X	X	X	X	X

For peer review only - http://bmjopen.bmj.com/site/about/guidelines.xhtml

blood	for bacterial detection (broad-range and specific PCR and next-generation sequencing)							
**Immunology substudy only**								
Whole blood	*In vitro* binding to bacterial products, cytokine expression and cellular responses to PAMP stimulation[4]	TROPGAN, Zvitambo	All	X	X	X	X	X
Plasma	Co-culture with healthy immune cells[5]	QMUL, London	All	X	X	X	X	X

[1]If HIV test, CD4 and viral load have already been conducted as part of routine clinical care, they will not be repeated on the research sample.
[2]THP1 reporter cells are derived from THP1, a human monocytic cell line that naturally expresses many pattern recognition receptors (PRR). The cell line stably expresses an NF-κB/AP-1 inducible reporter (SEAP) system to facilitate the monitoring of PRR-induced NF-κB/AP-1 activation.
[3]Assays will be undertaken in a subgroup of children, using a case-control or case-cohort design to evaluate the impact of biomarkers on immune activation and mortality.
[4]Whole blood will be stimulated with pathogen-associated molecular patterns (PAMP) in culture plates and bacterial antigens labelled with fluorescent tags in test tubes, and incubated for 1-24hr. Supernatant will be removed and stored at -80C for subsequent analysis of pro- and anti-inflammatory cytokines, and cells will be fixed for subsequent analysis of bacterial binding, cellular activation, proliferation and cytokine elaboration by flow cytometry.
[5]To determine the effect of the systemic milieu on healthy immune cell function, plasma samples will be transported to the Blizard Institute, QMUL and co-cultured with healthy immune cells, which will be functionally analysed via multi-parameter flow cytometry in the Flow Cytometry Core Facility.

IFABP: Intestinal fatty acid binding protein; ELISA: Enzyme-linked immunosorbent assay; GLP-2: glucagon-like peptide 2; sCD14: soluble CD14; sCD163: soluble CD163; PAMP: pathogen-associated molecular pattern; QMUL: Queen Mary University of London; CRP: C-reactive protein; LAL: limulus amoebocyte lysate assay.

For peer review only - http://bmjopen.bmj.com/site/about/guidelines.xhtml

Supplementary table 2: Additional laboratory analyses for enteropathy substudy

Sample type	Assay (method)	Location of work	Study groups[1]	Baseline	Discharge	Week 12	Week 24	Week 48
Urine	Lactulose-mannitol ratio (mass spectrometry)	Orgeon Analytics, USA	A, B, C, D (all)	X	X	X		X
Stool	Neopterin, myeloperoxidase, alpha-1 antitrypsin and REG-1B (ELISA)	TROPGAN and Zvitambo	A, B, C, D (all)	X	X	X	X	X
Plasma	I-FABP (ELISA)	TROPGAN and Zvitambo	A, B, C, D (all)	X	X	X	X	X
Plasma	GLP-2	TROPGAN and Zvitambo	A, B, C, D (all)	X	X	X	X	X
Plasma	Citrulline (mass spectrometry)	Imperial College London	A, B, C, D (all)	X	X	X	X	X
Plasma	Kynurenine:tryptophan ratio and metabolites along tryptophan pathway (mass spectrometry)	Imperial College London	A, B, C, D (all)	X	X	X	X	X
Stool	Microbiome analysis[2]	BCCDC, Vancouver	A, B, C, D (all)	X	X	X	X	X
Stool	*Helicobacter pylori* antigen	TROPGAN and Zvitambo	A, B, C, D (all)	X				
Gastric juice	Culture and molecular techniques for bacterial detection (broad-range and specific PCR and next-generation sequencing)	QMUL, London	Subgroup of A, C (n=50 per group)	X				

RNA extracted from PAXGene tubes	Gene expression analysis (RNASeq)	QMUL, London	A, B, C, D (all)	X	X			
Plasma and urine	Targeted and untargeted metabolic phenotyping	Imperial College London	A, B, C, D (all)	X	X	X	X	X

[1]Enteropathy substudy groups: Group A:,HIV-positive children with severe acute malnutrition; Group B: HIV-positive well-nourished controls; Group C: HIV-negative children with severe acute malnutrition; Group D: HIV-negative well-nourished controls.

Note that controls only have blood taken at baseline.

[2]For microbiome analyses,total DNA and/or RNA will be extracted from stool samples and used as template for next generation sequencing library preparation and for quantitative polymerase chain reaction (qPCR), then sequenced via whole metagenome shotgun sequencing

QMUL: Queen Mary University of London; BCCDC: British Columbia Centre for Disease Control.

For peer review only

For peer review only - http://bmjopen.bmj.com/site/about/guidelines.xhtml

BMJ Open

Health Outcomes, Pathogenesis and Epidemiology of Severe Acute Malnutrition (HOPE-SAM): rationale and methods of a longitudinal observational study

Journal:	*BMJ Open*
Manuscript ID	bmjopen-2018-023077.R2
Article Type:	Protocol
Date Submitted by the Author:	25-Oct-2018
Complete List of Authors:	Bwakura-Dangarembizi, Mutsa; University of Zimbabwe College of Health Sciences, Paediatrics and Child Health<br>Amadi, Beatrice; 2Tropical Gastroenterology and Nutrition Group, University of Zambia, Lusaka, Zambia<br>Bourke, Claire ; Blizard Institute, Queen Mary University of London, UK<br>Robertson, Ruairi; Blizard Institute, Queen Mary University of London, UK<br>Mwapenya, Benjamin; Zvitambo Institute for Maternal and Child Health Research, Harare, Zimbabwe<br>Chandwe, Kanta; Tropical Gastroenterology and Nutrition Group, University of Zambia, Lusaka, Zambia<br>Kapoma, Chanda; Tropical Gastroenterology and Nutrition Group, University of Zambia, Lusaka, Zambia, Paediatrics; Livingstone Central Hospital, Paediatrics<br>Chifunda, Kapula; Tropical Gastroenterology and Nutrition Group, University of Zambia, Lusaka, Zambia, paediatrics and child health<br>Majo, Florence; Zvitambo Institute for Maternal and Child Health Research, Harare, Zimbabwezi<br>Ngosa, Deophine; Tropical Gastroenterology and Nutrition Group, University of Zambia, Lusaka, Zambia<br>Chakara, Pamela; Zvitambo Institute for Maternal and Child Health Research, Harare, Zimbabwe<br>Chulu, Nivea; 2Tropical Gastroenterology and Nutrition Group, University of Zambia, Lusaka, Zambia<br>Masimba, Faithfull; Zvitambo Institute for Maternal and Child Health Research, Harare, Zimbabwe<br>Mapurisa, Idah; Zvitambo Institute for Maternal and Child Health Research, Harare, Zimbabwe<br>Besa, Ellen; Zvitambo Institute for Maternal and Child Health Research, Harare, Zimbabwe<br>Mutasa, Kuda; Zvitambo Institute for Maternal and Child Health Research, Harare, Zimbabwe<br>Mwakamui, Simutanyi; 2Tropical Gastroenterology and Nutrition Group, University of Zambia, Lusaka, Zambia<br>Runodamoto, Thompson ; Zvitambo Institute for Maternal and Child Health Research, Harare, Zimbabwe<br>Humphrey, Jean; Zvitambo Institute for Maternal and Child Health Research, Harare, Zimbabwe<br>Ntozini, Robert; Zvitambo Institute, Biostatistics & IT

	Wells, Jonathan C. K.; UCL Great Ormond Street Institute of Child Health<br>Manges, Amee; University of British Columbia<br>Swann, Jon; Imperial College London<br>Walker, Sarah; MRC Clinical Trials Unit at UCL<br>Nathoo, Kusum; University of Zimbabwe, College of Health Sciences<br>Kelly, Paul; Barts and The London School of Medicine<br>Prendergast, Andrew ; Queen Mary University of London,
**Primary Subject Heading**:	Global health
Secondary Subject Heading:	Epidemiology, Nutrition and metabolism, Paediatrics, Immunology (including allergy)
Keywords:	Malnutrition, HIV, Africa, mortality, microbiota, enteropathy

SCHOLARONE™
Manuscripts

Health Outcomes, Pathogenesis and Epidemiology of Severe Acute Malnutrition (HOPE-SAM): rationale and methods of a longitudinal observational study

Mutsa Bwakura-Dangarembizi[1], Beatrice Amadi[2], Claire D Bourke[3], Ruairi C Robertson[3], Benjamin Mwapenya[4], Kanta Chandwe[2], Chanda Kapoma[2], Kapula Chifunda[2], Florence Majo[4], Deophine Ngosa[2], Pamela Chakara[4], Nivea Chulu[2], Faithfull Masimba[4], Idah Mapurisa[4], Ellen Besa[2], Kuda Mutasa[4], Simutanyi Mwakamui[2], Thompson Runodamoto[4], Jean H Humphrey[4], Robert Ntozini[4], Jonathan Wells[5], Amee R Manges[6], Jonathan Swann[7], A Sarah Walker[8], Kusum J Nathoo[1], Paul Kelly[2,3], Andrew J Prendergast[3,4] for the HOPE-SAM Study Team[9].

[1]Department of Paediatrics and Child Health, University of Zimbabwe College of Health Sciences, Harare, Zimbabwe

[2]Tropical Gastroenterology and Nutrition Group, University of Zambia, Lusaka, Zambia

[3]Blizard Institute, Queen Mary University of London, UK

[4]Zvitambo Institute for Maternal and Child Health Research, Harare, Zimbabwe

[5]UCL Great Ormond Street Institute of Child Health, London, UK

[6]University of British Columbia, Vancouver, Canada

[7]Imperial College London, UK

[8]MRC Clinical Trials Unit at UCL, London, UK

[9]Other members of the HOPE-SAM study team are listed in the Acknowledgements section.

Corresponding author: Dr Mutsa Bwakura-Dangarembizi, Senior Lecturer, Department of Paediatrics and Child Health, University of Zimbabwe College of Health Sciences, Box

For peer review only

A178, Avondale, Harare, Zimbabwe. Email: mbwakura@medsch.uz.ac.zw. Tel +263 772 601 735. Fax +263 4 700 877.

Word count 5926

Abstract 261

Key words: Malnutrition, HIV, Africa, mortality, microbiota, enteropathy, immunology

For peer review only

For peer review only - http://bmjopen.bmj.com/site/about/guidelines.xhtml

ABSTRACT

Introduction

Mortality among children hospitalised for complicated severe acute malnutrition (SAM) remains high despite the implementation of WHO guidelines, particularly in settings of high HIV prevalence. Children continue to be at high risk of morbidity, mortality and relapse after discharge from hospital although long-term outcomes are not well documented. Better understanding the pathogenesis of SAM and the factors associated with poor outcomes may inform new therapeutic interventions.

Methods and analysis

The Health Outcomes, Pathogenesis and Epidemiology of Severe Acute Malnutrition (HOPE-SAM) study is a longitudinal observational cohort that aims to evaluate the short- and long-term clinical outcomes of HIV-positive and HIV-negative children with complicated SAM, and to identify the risk factors at admission and discharge from hospital that independently predict poor outcomes. Children aged 0-59mo hospitalised for SAM are being enrolled at three tertiary hospitals in Harare, Zimbabwe, and Lusaka, Zambia. Longitudinal mortality, morbidity and nutritional data are being collected at admission, discharge and for 48 weeks post-discharge. Nested laboratory substudies are exploring the role of enteropathy, gut microbiota, metabolomics and cellular immune function in the pathogenesis of SAM using stool, urine and blood collected from participants and from well-nourished controls.

Ethics and dissemination

The study is approved by the local and international institutional review boards in the participating countries (the Joint Research Ethics Committee of the University of Zimbabwe, Medical Research Council of Zimbabwe and University of Zambia Biomedical

For peer review only

Research Ethics Committee) and the study sponsor (Queen Mary University of London). Caregivers provide written informed consent for each participant. Findings will be disseminated through peer-reviewed journals, conference presentations and to caregivers at face-to-face meetings.

Strengths and limitations of this study

Strengths:

- Rigorous collection of longitudinal data on morbidity, mortality and nutritional status during inpatient care and for 48 weeks after initial admission for SAM in HIV-positive and HIV-negative children.
- Laboratory sub-studies investigating enteropathy, microbiota, metabolomics and immune cell function provide a unique opportunity to understand which pathogenic pathways contribute to SAM and whether these processes normalise with nutritional rehabilitation.

Potential limitations:

- High loss to follow-up due to participants returning to home settings following hospital discharge.
- The clinical heterogeneity of the study participants, including comorbidities such as infections, may make it challenging to identify the specific causes of clinical outcomes.
- Potential bias in recruiting well-nourished controls only from hospitals will be reduced by inclusion of community-based controls, including well-nourished siblings of children with SAM.

INTRODUCTION

For peer review only

Malnutrition underlies almost half of all childhood deaths in developing countries[1]. Severe acute malnutrition (SAM) is defined by a weight-for-height Z-score <-3, mid-upper arm circumference (MUAC) <115mm and/or bilateral pitting oedema[2]. Current treatment guidelines distinguish two groups: i) children with uncomplicated SAM who can be managed in the community; and ii) children with complicated SAM, who are hospitalised and undergo resuscitation, stabilisation and nutritional rehabilitation. In-hospital mortality in children with complicated SAM remains high despite the implementation of WHO guidelines[3]. Furthermore, SAM presents as two major clinical phenotypes: non-oedematous SAM (marasmus), characterised by severe wasting, and oedematous SAM (kwashiorkor), a more complex syndrome characterised by bilateral pitting oedema, steatosis and diarrhea[4 5]. Despite differing clinical outcomes, treatment protocols are the same for both oedematous and non-oedematous SAM.

A contributory factor to high in-patient mortality is the co-occurrence of HIV infection in around one-third of children hospitalised for SAM in sub-Saharan Africa[6 7]. While new HIV infections in children have declined[8], a substantial number of infected children are diagnosed late and present with malnutrition. There is also a growing population of HIV-exposed uninfected (HEU) children who have immune abnormalities, poor growth and higher risk of mortality and infectious morbidity[9]. Hence, HIV has transformed the epidemiology and outcomes of SAM[10]. Even with standardised treatment approaches, inpatient deaths are almost four-fold higher among HIV-positive children with SAM (herein termed HIV-SAM), compared to HIV-negative children with SAM (30.4% vs 8.4%), for reasons that remain unclear[10]; this mortality is three-fold higher than would be expected from anthropometric parameters alone[10]. Management of HIV-SAM is particularly challenging because HIV fundamentally alters the clinical presentation of malnutrition and the response to treatment. Children with HIV-SAM are more stunted

For peer review only - http://bmjopen.bmj.com/site/about/guidelines.xhtml

and wasted; have a higher frequency of persistent diarrhoea; tend to have delayed nutritional recovery and have a more complicated clinical course than HIV-negative children with SAM[10].

Long-term outcomes of SAM

Following resolution of complications and return of appetite, children are discharged from hospital to continue therapeutic feeds at home. However, emerging data indicate high post-discharge mortality following in-hospital management of SAM[11-13]. Malnutrition together with young age, HIV infection and pneumonia have been associated with higher post-discharge mortality[14]. One of the largest prospective studies of growth and mortality in children with SAM (FuSAM), conducted in Malawi from July 2006 to March 2007, collected 12-month outcome data on 87% of 1024 children admitted to the nutrition ward[11]. A total of 427 (42%) died and 44% of these deaths occurred after discharge from hospital. Survival was greatest among those who were nutritionally cured upon discharge from outpatient therapeutic feeding centres, defined as two consecutive visits with >80% expected weight-for-height, no oedema and clinically stable. The risk of mortality after hospital discharge was four-fold higher for HIV-SAM compared to HIV-negative children with SAM, but the outcomes among HEU children were not reported. The loss to follow-up was high in the FuSAM study because there was only one follow-up visit, one year after discharge from outpatient-feeding centres. A recent study from Kenya identified malnutrition and HIV infection as key drivers for post-discharge mortality, with 52% of deaths attributable to MUAC <11.5cm and 11% to HIV infection[15].

The impact of SAM appears to persist beyond the first year after discharge from hospital. The ChroSAM study, which followed children with SAM seven years post-discharge, showed that children had poorer growth, body composition and physical function

For peer review only

compared to siblings and community controls, which are all indicators of future cardiovascular and metabolic disease[12].

While anthropometry is used to assess nutritional recovery after discharge from hospital, the pattern and quality of growth recovery following SAM is poorly understood. The observation that children treated for SAM have a deficit in lean tissue despite regaining weight suggests that assessing body composition in addition to anthropometry may help to identify children who have not completely recovered and are at potential risk of long-term metabolic diseases[12]. Children with HIV-SAM appear to have potential for catch-up growth in weight-for-age and/or weight-for-height, which have been shown to normalise with treatment even prior to widespread availability of ART[16]; by contrast, height-for-age shows less potential for catch-up growth[17]. However, the body composition of children with HIV-SAM compared to HIV-negative children with SAM has not been described. Whether children recover fat mass at the expense of lean mass is unknown, but differences in tissue accretion patterns may have implications for survival and long-term metabolic health[18 19]. There is also a need to consider the effect of SAM on the size of body parts which grow at different rates: relatively shorter legs, for example, are associated with epidemiologic risk of overweight, coronary artery disease, liver dysfunction and diabetes[20 21].

Taken together, there is clearly an elevated risk of mortality among HIV-positive children with SAM compared to HIV-negative children with SAM, and an ongoing mortality risk among all children with SAM that persists after discharge from hospital. There are several gaps in our understanding of the long-term outcomes: (i) causes of death have not been clearly defined; (ii) no studies have systematically and longitudinally collected morbidity and mortality data or documented repeat hospitalisations post-discharge; and,

For peer review only

(iii) the long-term outcomes of HIV-positive children with SAM in the era of ART availability are unclear.

Pathogenesis of SAM

Better understanding the pathogenesis of SAM may help to explain the high mortality of children both during and after hospitalisation and identify new targets for interventions to supplement existing treatment strategies. Consistent evidence that immune mediators are altered in malnutrition[22] and that systemic and intestinal inflammation are associated with poor outcomes in SAM[23], suggest that immune dysfunction contributes to infectious susceptibility[24]. Malnutrition is also characterised by a complex derangement in gut microbial[25] metabolic,[26] immune[27] and hormonal pathways, organ dysfunction and micronutrient deficiencies in the context of co-infections, enteropathy and chronic inflammation. Several studies have recently provided insights into these perturbations using new tools[25 26 28 29], including metabolomics and metagenomics, but we still lack a clear understanding of many of the pathogenic pathways driving malnutrition, the interactions between these pathways, and which are the most tractable targets for intervention.

SAM shares several pathological and clinical features with HIV, which may explain clinical outcomes in these co-occuring conditions: 1) both are characterised by intestinal damage, leading to impairment of the mucosal barrier and increased intestinal permeability; 2) both have underlying systemic immune activation; and 3) both are frequently complicated by persistent diarrhoea, pneumonia and sepsis that may plausibly arise due to loss of intestinal barrier function[30]. Understanding the overlapping impact of HIV and SAM is critical to inform additional interventions to improve outcomes of children with HIV-SAM.

For peer review only - http://bmjopen.bmj.com/site/about/guidelines.xhtml

OBJECTIVES OF HOPE-SAM

The Health Outcomes, Pathogenesis and Epidemiology of Severe Acute Malnutrition (HOPE-SAM) study has two primary objectives:

1) To describe the short- and long-term clinical outcomes of children with complicated SAM, with and without HIV infection, and to identify the risk factors at admission and discharge from hospital that independently predict these outcomes.
2) To better characterise the pathogenesis of SAM through nested laboratory sub-studies evaluating enteropathy, gut microbiota, metabolomics and immune cell function.

STUDY DESIGN

HOPE-SAM is a longitudinal observational cohort study, enrolling between 600-800 children aged 0-59 months admitted with complicated SAM to the tertiary pediatric wards at two sites in Zimbabwe (Parirenyatwa Hospital and Harare Children's Hospital) and one in Zambia (University Teaching Hospital, Lusaka). Both HIV-positive and HIV-negative children will be enrolled. Throughout this paper, 'SAM' refers to all children, regardless of HIV status; where analyses specifically compare children by HIV status, groups are identified as HIV-positive children with SAM (or HIV-SAM) and HIV-negative children with SAM. All participants with SAM are followed for 48 weeks post-discharge, with longitudinal data collection and blood sampling. The study contains four nested sub-studies as shown in **Figure 1.** A subgroup of children will be recruited to the enteropathy substudy for which they will have the same follow-up procedures but more intensive biological specimen collection including stool (all time-points), urine after lactulose-mannitol (LM) challenge as an assessment of intestinal permeability, and

For peer review only

nasogastric aspirate (baseline only); these children are also included in microbiota and metabolomics substudies. Children with SAM for whom blood samples are available are included in the immunology substudy, for which circulating inflammatory mediators will be assayed; functional cellular immunology assays will be conducted for all children in the immunology sub-study with sufficient sample volume (> 2mL) recruited after June 2017. A group of healthy children recruited from the same hospitals and communities, who are well-nourished and matched to children in the enteropathy substudy by age and HIV status, will have data and specimens collected to provide normative data for the laboratory substudies; these well-nourished controls will not be followed longitudinally.

The study protocol, data collection forms and standard operating procedures are available online at osf.io/29uaw.

RECRUITMENT

Screening: Caregivers of all hospitalised children are sensitised about the study. All new admissions aged 0-59 months are screened for SAM, which is defined according to WHO criteria as any of: weight-for-height Z-score (WHZ) <-3, MUAC <115 mm (if aged 6-59mo) and/or bilateral pitting oedema. All children with SAM are recruited from hospital and this study therefore focuses on complicated SAM; children with uncomplicated SAM will not be enrolled.

Eligibility for observational cohort: All children with SAM whose caregivers are willing to provide written informed consent and to learn their child's HIV status are offered enrolment. Any children who die prior to study enrolment and those with a known malignancy are ineligible.

For peer review only - http://bmjopen.bmj.com/site/about/guidelines.xhtml

Eligibility for enteropathy substudy: Children with SAM aged 6-59 months with a nasogastric tube in place (or due to be placed) are categorized into 4 groups (HIV-positive oedematous (Group A-I); HIV-positive non-oedematous (Group A-II); HIV-negative oedematous (Group C-I) and HIV-negative non-oedematous (Group C-II), as shown in Table 1. Children meeting eligibility criteria will be enrolled throughout the study recruitment period until sufficient specimens have been collected from the groups shown in Table 1. Children in the enteropathy substudy are stratified into age bands (6-11 months; 12-23 months and 24-59 months) to enable age-matching of well-nourished controls. Children with underlying chronic gastrointestinal disease or a known malignancy are ineligible.

Table 1: Enteropathy substudy groups

**Children aged 6-59 months**	**Severe acute malnutrition[1]**		**Well nourished controls** WHZ>-1
	**Oedematous[2]**	**Non-oedematous**	
**HIV-positive** (HIV PCR+ if <18mo; HIV antibody + if >18mo)	N=50 (Group A-I)	N=50 (Group A-II)	N=100[3] (Group B)
**HIV-negative** (HIV PCR- if <18mo; HIV antibody - if >18mo)	N=50 (Group C-I)	N=50 (Group C-II)	N=100[4] (Group D)

[1]SAM defined according to WHO criteria
[2]Presence of bilateral pitting pedal oedema.
Note that children below 6 months of age are excluded from the enteropathy substudy to avoid interrupting exclusive breastfeeding during the lactulose-mannitol test.
WHZ: Weight-for-height Z score; PCR: polymerase chain reaction.

Eligibility for microbiota and metabolomics substudies: Children enrolled into the enteropathy substudy are also included in the microbiota and metabolomics substudies, since these substudies utilize the stool, urine and plasma samples collected for enteropathy analyses.

For peer review only

For peer review only - http://bmjopen.bmj.com/site/about/guidelines.xhtml

Eligibility for immunology substudy: The immunology substudy comprises all children with SAM (drawn from both the observational cohort and the enteropathy substudy, as shown in Figure 1) providing a blood sample of sufficient volume (>2ml) for cellular assays after 1st June 2017.

Well-nourished controls: Controls are children drawn from the same hospitals and communities as cases with SAM (including well-nourished sibling controls), who are aged 6-59 months (matched to enteropathy substudy children within age bands), well-nourished (weight-for-height Z-score >-1) and clinically well (no acute illness or current infections) with known HIV status. Controls are categorized into two groups: well-nourished HIV-positive (Group B) and well-nourished HIV-negative (Group D), as shown in Table 1. Children with underlying chronic gastrointestinal disease or a known malignancy are ineligible. Well-nourished controls provide comparison biomarker data for all the laboratory substudies.

Informed consent procedures: Written informed consent is obtained from the primary caregiver using consent forms translated into local languages; where possible, other family members are included in the consent process. Illiterate caregivers who have understood a verbal explanation of the study can provide a thumb imprint in the presence of a witness. Assent from children is not sought because all are <5 years old.

STUDY PROCEDURES

Study procedures are outlined in **Table 2**.

Table 2: Summary of procedures in observational cohort

For peer review only - http://bmjopen.bmj.com/site/about/guidelines.xhtml

For peer review only

For peer review only

Assessment	Hospitalization		Post-discharge[3]				
	Baseline[1]	Discharge[2]	2w	4w	12w	24w	48w
Caregiver informed consent to join observational cohort	x						
Summary checklist	x						
Locator information[4]	x						
Acute admission information	x						
Baseline data	x						
Daily clinical review[5]	Daily during hospitalisation						
Blood collection[6]	x	x			x	x	x
HIV testing[7]	x						
CD4 count and viral load (HIV-infected children only)	x				x	x	x
Full blood count[8]	x	x			x	x	x
Anthropometry	x	x	x	x	x	x	x
Skinfold thickness[9]		x	x	x	x	x	x
Body composition[10]	x	x	x	x	x	x	x
Discharge data collection		x					
Daily morbidity diary			Daily during follow-up period by caregivers				
Follow-up clinic: history, examination, morbidity and mortality data			x	x	x	x	x

[1] Children will be enrolled as soon as possible after hospitalisation and will undergo baseline investigations as soon as possible after enrolment. This is to provide a window of opportunity to time collection of research specimens with clinical specimens, and to ensure that the child is clinically stable before undertaking research investigations.
[2] The discharge procedures will be undertaken on the day of discharge, or as close as possible to that date.
[3] Windows will be created around these post-discharge time-points to maximize follow-up for caregivers who miss visits or are unavailable, as follows: 2 weeks (1-3 weeks); 4 weeks (3-5 weeks); 12 weeks (10-14 weeks); 24 weeks (20-28 weeks); 48 weeks (44-52 weeks).
[4] Locator information will updated at subsequent visits if caregivers have moved or changed contact details.
[5] A clinical review will be undertaken every day between admission and discharge by the study clinician.
[6] 5.4 mL of blood (depending on child weight; amount will not exceed 2 mL/kg total over 2 week period) will be collected by a study nurse into endotoxin-free EDTA tubes. Samples will be used to store whole blood, PBMC and plasma for subsequent measurement of CRP and albumin. Where blood sample volumes allow (≥2mL sample), bacterial binding assays and whole blood stimulations will be conducted and culture supernatants and cells stored for subsequent assessment of immune cell function at each time-point.

Study blood samples will not be collected from children with known haemoglobin <6 g/dL.
[7]HIV testing is conducted as part of routine clinical practice, but if it has not been undertaken, the study sample will be used to test for HIV, as stated in the informed consent form, since HIV status is required to allocate children to study groups.
[8]Full blood count results will be transcribed from clinical records; if not done by clinical teams, the EDTA sample will be used to measure FBC in clinical laboratories at each site
[9]Skinfold thickness (triceps, subscapular, supra-iliac) and mid-thigh circumference will be measured using Holtain calipers or tape measure.
[10]Body composition will be assessed by bioimpedance vector analysis.

Baseline procedures:

Baseline data on maternal and household characteristics, the child's past medical history and current illness are collected by a study nurse. Anthropometry, including body composition measured by whole-body (wrist-ankle) bio-electrical impedance analysis (BodyStat 1500MD; BodyStat Ltd., Douglas, Isle of Man), leg length using an electronic knemometer (Zimbabwe only, due to availability of knemometers) and triceps, subscapular and supra-iliac skinfold thickness using calipers (Holtain Ltd., Crymych, UK) are undertaken at baseline. Blood (1mL/kg up to 5.4mL maximum) is collected at baseline into an endotoxin-free EDTA tube for all children and, in the enteropathy substudy, additionally into a PAXgene tube (PreAnalytiX GmbH, Hombrechtikon, Switzerland) for subsequent transcriptomic analysis. Blood is not collected from children with severe anaemia (known haemoglobin <6 g/dL). HIV testing is carried out in accordance with national guidelines as part of routine clinical practice; where it has not been done, the child's HIV status is ascertained using a rapid test antibody algorithm for children over 18 months, or HIV DNA PCR for children under 18 months. CD4 count/percentage and viral load are measured in HIV-positive children. Maternal HIV status is documented where available, so that HIV-exposed uninfected children can be identified. Blood samples are sent to research laboratories at each site to conduct whole blood stimulation and bacterial binding assays (as described in the immunology substudy) and to store aliquots of whole blood, peripheral blood cells and plasma at -

For peer review only

80ºC[31]. In the enteropathy substudy, nasogastric aspirate, stool and urine (after an oral dose of lactulose and mannitol) are also collected. Lactulose and mannitol are ingested by the child after fasting and urine is collected over a two-hour period to measure recovery of lactulose and mannitol, a measure of intestinal absorptive capacity and permeability, as previously described[32].

Daily procedures: Routine inpatient management is undertaken by ward clinical teams according to local hospital protocols, which are based on WHO guidelines[2].[33] In addition, the HOPE-SAM study clinician at each hospital site collects daily data until discharge on clinical parameters (including daily examination), resolution of acute infections, nutritional recovery (loss of oedema, restoration of appetite, weight gain), and treatment/nutritional supplements received; this will allow us to evaluate differences in management between countries. Children with HIV-SAM who are ART-naïve start ART according to national guidelines, which are based on WHO recommendations[2 34].

Discharge: The clinical team decides when the child is ready to be discharged, which is generally when their medical complications are resolving and the child has a good appetite and is clinically well and alert[2]. Children receive ready-to-use therapeutic feeds (RUTF) to take at home according to local guidelines. At discharge, the study nurse collects data and a repeat blood sample (including full blood count) and undertakes discharge anthropometry, body composition, leg length (Zimbabwe only) and triceps, subscapular and supra-iliac skinfold thickness measurements (**Table 2**). The caregiver is given a daily morbidity diary and pre-prepared stickers corresponding to different illnesses and shown how to complete the diary. The caregiver is provided with the date of the first follow-up appointment and contact details of the study nurse.

For peer review only - http://bmjopen.bmj.com/site/about/guidelines.xhtml

Follow-up: Children attend follow-up appointments at dedicated study clinics at 2, 4, 12, 24 and 48 weeks post-discharge. At each visit, the study physician undertakes a clinical assessment and the study nurse captures illness, medication and feeding data. Clinic data are transcribed from handheld medical records if available and the morbidity diary is reviewed and a new diary and stickers supplied. Anthropometry, body composition, leg length (Zimbabwe only) and triceps, subscapular and supra-iliac skinfold thicknesses are measured at each visit. Acute illnesses are treated in the study clinic, or the child is referred to hospital if necessary. Children with relapsed malnutrition are provided with nutritional supplements or RUTF according to local guidelines, or readmitted to hospital if they develop complicated SAM. Transport reimbursement for clinic attendance is provided to caregivers for each visit.

Blood is collected at weeks 12, 24 and 48 post-discharge into endotoxin-free EDTA tubes to measure full blood count, CD4 count and viral load (HIV-positive children only), conduct whole blood stimulation and bacterial binding assays (where blood volumes >2mL), and store peripheral blood cells and plasma aliquots for subsequent analysis (all blood samples), including soluble and cellular markers of immune activation, as outlined in **Supplementary Table 1**. Children in the enteropathy substudy have additional stool and urine collection following lactulose-mannitol dosing as shown in **Table 3.**

Table 3: Summary of procedures for cases in the enteropathy substudy

Assessment	Hospitalization		Post-discharge[3]				
	Baseline[1]	Discharge[2]	2w	4w	12w	24w	48w
Caregiver informed consent to join observational cohort and enteropathy substudy	X						

For peer review only - http://bmjopen.bmj.com/site/about/guidelines.xhtml

Summary checklist	X						
Locator information[4]	X						
Acute admission information	x						
Baseline data	X						
Daily clinical review[5]	Daily during hospitalisation						
Blood collection[6]	x	x			x	x	x
HIV testing[7]	X						
CD4 count and viral load (HIV-infected children only)	X				x	x	x
Full blood count[8]	X	x			x	x	x
Gastric aspirate[9]	x						
Stool collection[10]	x	x			x	x	x
Lactulose-mannitol testing[11]	x	x			x		x
Anthropometry	X	x	x	x	x	x	x
Skinfold thickness[12]		x	x	x	x	x	x
Body composition[13]	X	x	x	x	x	x	x
Discharge data collection		x					
Daily morbidity diary			Daily during follow-up period by caregivers				
Follow-up clinic: history, examination, morbidity and mortality data			x	x	x	x	x

[1]Children will be enrolled within 24h of hospitalization and will undergo baseline investigations within 72h of hospitalization. This is to provide a window of opportunity to time collection of research specimens with clinical specimens, and to ensure that the child is clinically stable before undertaking research investigations.
[2]The discharge procedures will be undertaken on the day of discharge, or as close as possible to that date.
[3]Windows will be created around these post-discharge time-points to maximize follow-up for caregivers who miss visits or are unavailable, as follows: 2 weeks (1-3 weeks); 4 weeks (3-5 weeks); 12 weeks (10-14 weeks); 24 weeks (20-28 weeks); 48 weeks (44-52 weeks).
[4]Locator information will updated at subsequent visits if caregivers have moved or changed contact details.
[5]Daily clinical review will be conducted every day between admission and discharge by the study clinician.
[6]During hospitalisation, 5.4 mL of blood (depending on child weight; amount will not exceed 2 mL/kg total over 2 week period) will be collected by a study nurse into a 2.7 mL endotoxin-free EDTA tube and

For peer review only - http://bmjopen.bmj.com/site/about/guidelines.xhtml

a 2.7 mL PAXGene tube, for subsequent isolation of RNA and gene expression analysis. After discharge (weeks 12, 24 and 48), 5.4 mL of blood (depending on child weight; amount will not exceed 2 mL/kg total over 2 week period) will be collected by a study nurse into two 2.7 mL endotoxin-free EDTA tubes.

[7]HIV testing is conducted as part of routine clinical practice, but if it has not been undertaken, the study sample will be used to test for HIV (see section 9.4), as stated in the informed consent form, since HIV status is required to allocate children to study groups.

[8]Full blood count results will be transcribed from clinical records; if not done by clinical teams, the EDTA sample will be used to measure FBC in clinical laboratories at each site

[9]A gastric juice sample will be collected at the same time as the blood draw by aspirating the nasogastric tube with a sterile feeding syringe, to test for gastric pH; sterile water or saline will then be instilled and a sample of gastric juice collected for storage for subsequent PCR and culture (section 7.5.2)

[10]Stool collection will be undertaken at the same time as the blood draw

[11]Lactulose-mannitol testing will be conducted, with collection of a baseline urine sample, followed by a 2hr urine collection post-LM ingestion. This test will be deferred until children are judged to be clinically stable by the study physician during daily reviews. In general, this will be a child in the nutritional rehabilitation phase, who has no cardiorespiratory compromise.

[12]Skinfold thickness (triceps, subscapular, supra-iliac) and mid-thigh circumference will be measured using Holtain calipers or tape measure.

[13]Body composition will be assessed by bioimpedance vector analysis.

Caregivers are reminded of follow-up visits by phone, and visit completion is tracked on a dedicated database. If caregivers do not attend follow-up appointments, attempts are made to contact them by phone and home visits are made if feasible, particularly for those defaulting the 48-week visit, so that long-term outcome data can be collected. For post-discharge deaths, a home visit is undertaken by study nurses where possible to conduct a verbal autopsy. Children who are readmitted to one of the study sites with relapsed SAM have data collected during the new episode of hospitalisation. The study ends for each participant at the week 48 visit.

SUBSTUDIES

As outlined in Figure 1, four nested substudies will utilise biological specimens to address mechanistic questions related to enteropathy, microbiota, metabolomics and immune function.

Enteropathy substudy

For peer review only

For peer review only - http://bmjopen.bmj.com/site/about/guidelines.xhtml

The gut, which acts as an internal interface between humans and the environment, must contain the nutrient stream and the symbiotic microbiota while allowing molecular intimacy to permit absorption. The mechanism underlying this duality is the integrity of the gastrointestinal barrier; intestinal damage (enteropathy) can impair this critical barrier function. A spectrum of enteropathies affect children in developing countries[30]. Environmental enteric dysfunction (EED), characterised by small intestinal inflammation, blunted villi and increased intestinal permeability, is almost universal and is morphologically indistinguishable from HIV enteropathy[30]. Children in resource-poor settings also suffer from frequent diarrhoea, food insecurity and micronutrient deficiencies, which all exacerbate enteropathy[30]. As a result, a cycle of intestinal infection, impaired mucosal function and malnutrition commonly arises, which may ultimately precipitate SAM, especially in the context of HIV infection[35 36]. It is not yet established if the enteropathy seen in children with SAM[37], which we here refer to as malnutrition enteropathy[37], is qualitatively or quantitatively distinguishable from EED. In addition to local intestinal pathology, enteropathies may cause systemic pathology due to persistent immune activation arising from enteric inflammation and microbial translocation across the damaged gut wall[30]. It is becoming apparent that chronic inflammation may be particularly deleterious in malnourished individuals[23]; in children with SAM, systemic inflammation arising from underlying enteropathy may further increase morbidity and mortality.

We hypothesize that i) the degree of enteropathy during hospitalisation differs between oedematous and non-oedematous SAM and is independently associated with morbidity, mortality and nutritional recovery during hospitalization; ii) the degree of enteropathy at discharge is independently associated with morbidity, mortality and relapse of SAM; and

For peer review only

iii) children with HIV-SAM have more severe enteropathy than HIV-negative children with SAM, which contributes to their poorer outcomes.

Using stored samples, a longitudinal series of investigations will compare gastric and small intestinal barrier function, using a range of biomarkers to capture the domains of malnutrition enteropathy (**Supplementary Table 2**). To understand better the extra-intestinal consequences of enteropathy, we will first compare the microbial composition of the upper gut and plasma using deep sequencing in a subgroup of children with paired gastric and blood samples. Secondly, we will undertake transcriptomics using PAXGene blood samples to determine i) whether there are differences in gene expression profiles between well-nourished controls, HIV-negative children with SAM and HIV-positive children with SAM (including comparison of oedematous and non-oedematous types); and ii) whether specific patterns of gene expression are associated with morbidity and mortality in SAM.

Microbiota substudy

Normal assembly of the gut microbiota in early life is critical for many aspects of physiological, neurological and immune development[38]. Recent evidence suggests that an immature or pathogenic microbiota plays a causative role in the pathogenesis of SAM[25]. For example, a number of microbial taxa have been identified, including *Faecalibacteium prausnitzii*, which discriminate and predict gut microbiota maturity and child growth[28]. Other pathogenic microorganisms, including IgA-targeted *Enterobacteriaceae*, are associated with impaired growth and may contribute to SAM[39]. Nutritional rehabilitation with RUTF induces temporary recovery of a disturbed microbiota; however, the microbiota appears to revert back to an immature diseased state following nutritional recovery[29]. HIV infection is also associated with a disturbed gut

For peer review only - http://bmjopen.bmj.com/site/about/guidelines.xhtml

microbiota[40], which may further compound enteropathy phenotypes. Furthermore, there is some evidence that differences exist in malnutrition enteropathy between oedematous and non-oedematous SAM[41]; however, few studies have investigated differences in the gut microbiota between the two forms of the disease.

We hypothesize that (i) a unique gut microbial signature exists in HIV-SAM, compared with HIV-negative children with SAM, that is independently associated with morbidity, mortality, nutritional recovery and degree of enteropathy during hospitalisation; (ii) a unique gut microbial signature exists in oedematous compared with non-oedematous SAM; (iii) specific microorganisms or gut microbial diversity indices are independently associated with morbidity, mortality, nutritional recovery and degree of enteropathy during hospitalisation; and (iv) the gut microbiota is partially restored to a healthy state with nutritional rehabilitation but reverts to a dysbiotic state during follow-up, which predicts morbidity, mortality and relapse of SAM.

Using stored stool samples collected at baseline, a cross-sectional investigation will determine differences in the gut microbial composition and predicted function between: HIV-negative children with SAM versus HIV-SAM, oedematous versus non-oedematous SAM, and well-nourished controls. Gut microbial composition and predicted function will be compared between groups at discharge and at 12, 24 and 48 weeks post-discharge. Briefly, total DNA and/or RNA will be extracted from stool samples and used as template for next-generation sequencing library preparation and for quantitative polymerase chain reaction (qPCR). Whole metagenome shotgun sequencing will be performed using the HiSeq 2500 system. Raw metagenomic sequencing data will be quality-filtered and analysed through a well-validated bioinformatics pipeline using MetaPhlAn[42] and HUMAnN[43]. The compositional and predicted functional metagenomic data generated

For peer review only - http://bmjopen.bmj.com/site/about/guidelines.xhtml

will be used to identify signatures of SAM and to investigate associative links between specific gut microbial signatures and clinical outcomes.

Metabolomics substudy

During SAM, metabolic processes are altered in response to a starved environment, and may plausibly contribute to long-term clinical outcomes. Previous studies suggest that amino acid turnover, lipid metabolism, oxidative stress and other metabolic pathways are disrupted in SAM and may be associated with disease state and clinical outcome[26 44 45]; however, little is known about how the metabolic phenotype responds to nutritional therapy. It is hypothesised that disturbed gut microbiota composition and function may drive microbial metabolic dysregulation in addition to host-derived dysregulation. Of particular interest are differences in the metabolic phenotype between oedematous and non-oedematous SAM. The 'reductive adaptation' seen in non-oedematous SAM (utilisation of fat and muscle stores) is disrupted in oedematous SAM, which may contribute to differences in clinical outcomes. Specifically, protein turnover, inflammation, oxidative stress and bile acid metabolism are disrupted in oedematous-SAM, which may contribute to co-morbidities including diarrhoea, steatosis and enteropathy[46 47].

We hypothesize that: (i) a unique plasma and urine metabolic phenotype exists in oedematous compared with non-oedematous SAM during hospitalisation, which is independently associated with morbidity, mortality and nutritional recovery; (ii) the metabolic phenotype is partially restored to a healthy state with nutritional rehabilitation but reverts to a disturbed state during follow-up, which predicts morbidity, mortality and relapse; and (iii) both host-derived and gut microbial-driven metabolic dysregulation underlie clinical outcomes.

For peer review only - http://bmjopen.bmj.com/site/about/guidelines.xhtml

Using stored urine and plasma samples collected during hospitalisation, a cross-sectional investigation will determine differences in the metabolic phenotype between children with oedematous SAM, non-oedematous SAM and well-nourished controls. Urine and plasma metabolic phenotypes will be compared between groups at discharge and 12, 24 and 48 weeks post-discharge. Briefly, global untargeted metabolomic phenotyping will be performed via ^{1}H nuclear magnetic resonance (NMR) spectroscopy using a 700 MHz Bruker NMR spectrometer to identify metabolic signatures of SAM. Targeted analysis via ultra-performance liquid chromatography-mass spectrometry will be performed to examine specific pathways of interest, including tryptophan and bile acid metabolism.

Immunology substudy

Bacterial infections are common among children hospitalised for SAM[23 48-50] and mortality is driven by a range of species[48 50-53], consistent with generalised defects in innate anti-bacterial defence. Increased infectious morbidity and mortality persist after discharge from hospital[10 17 54], suggesting that restoration of anti-bacterial immune responses may lag behind nutritional rehabilitation. A recent randomised trial in children with SAM confirmed that deaths following hospitalisation were predominantly due to bacterial infections but were not prevented by daily co-trimoxazole prophylaxis[17]. Collectively, these observations highlight that children remain vulnerable to infection despite current treatment approaches; targeting persistent immune dysfunction could plausibly reduce infectious mortality after discharge[24].

Multiple innate and adaptive immune mediators are dysregulated in malnutrition[24 27 55]. However, few studies have assessed cellular immune function in malnourished children;

For peer review only - http://bmjopen.bmj.com/site/about/guidelines.xhtml

most existing studies were undertaken decades ago on small cross-sectional cohorts without the benefit of recent advances in immunology techniques[27]. Immune dysfunction in SAM likely reflects both *intrinsic* defects, whereby immune cells lack capacity to adequately respond to infection, and *extrinsic* defects, where cells have intact anti-bacterial capacity but are chronically modulated by the systemic pro-inflammatory environment which characterises SAM (i.e. heightened pro-inflammatory cytokines[44] and circulating bacterial antigens[23 56 57]). Systemic inflammation is directly associated with mortality in SAM[23] and driven by multiple comorbidities, including bacterial translocation from the damaged gut into the blood, sub-clinical infections and metabolic dysregulation[44 58 59]. The implications of innate immune cell dysfunction for subsequent acquisition of infections and infectious mortality have not been investigated.

We hypothesise that: (i) anti-bacterial functions of innate immune cells are compromised in SAM due to a combination of intrinsic and extrinsic defects; ii) innate immune cell function is independently associated with infectious morbidity and mortality during hospitalisation for SAM; and iii) nutritional rehabilitation only partly restores innate immune cell function, leading to an ongoing risk of bacterial infections post-discharge.

Using blood samples collected at baseline, discharge and 12, 24 and 48 weeks post-discharge, the longitudinal relationship between circulating innate immune cell function and bacterial infections will be assessed. The intrinsic phagocytic capacity, secreted cytokine response and maturation state of innate immune cells after culture with bacterial antigens will be assessed. Plasma concentrations of endotoxin and pro-inflammatory mediators will be quantified at each time-point and the degree to which these extrinsic factors influence innate immune cell antibacterial function will be assessed via plasma co-culture with innate immune cells from healthy donors. Bacterial

For peer review only - http://bmjopen.bmj.com/site/about/guidelines.xhtml

infections during hospitalisation will be diagnosed using clinical criteria and blood culture, stool culture and urinalysis where available.

SAMPLE SIZES

Observational study: The observational cohort will recruit as many children with SAM as possible during the period of enrolment (July 2016 to March 2018), estimated at 600-800 children (capped at 800 maximum), to assess clinical and nutritional outcomes among HIV-positive and HIV-negative children hospitalised with SAM. Assuming mortality of 15%, overall loss to follow-up of 15% and recruitment target of 800 children, there would be 560 evaluable children at 48 weeks, of whom 224 would have HIV-SAM based on an estimated inpatient HIV prevalence of 40%. This will provide >80% power to detect absolute differences of 17% in binary outcomes between HIV-SAM and HIV-negative children with SAM, and of 0.33 times the standard deviation in continuous outcomes.

Enteropathy substudy: The sample size was estimated using previously reported values for LM ratios, which remain a widely used non-invasive marker of enteropathy. Comparing 100 versus 100 children with two-sided alpha=0.025 (to allow for two primary comparisons, i.e. HIV-SAM versus HIV-negative children with SAM, and HIV-SAM versus well-nourished HIV-positive children) provides >80% power to detect differences in mean LM ratio during hospitalisation of at least 0.16 (assuming SD=0.36), a difference which would be clinically relevant given the LM ratios previously reported for well-nourished children (0.42), malnourished children (1.3) and children with persistent diarrhoea (2.85) in the Gambia[60]. It also provides >80% power to detect differences of at least 0.1 in the mean change in LM ratio from enrolment (assuming SD for change=0.23 and 7% missing samples). For inflammatory markers, comparing 100 versus 100

For peer review only - http://bmjopen.bmj.com/site/about/guidelines.xhtml

children with two-sided alpha=0.025 provides >80% power to detect differences in mean log_{10} concentrations of at least 0.44 times their standard deviation, or 2.75-fold differences between groups. Inclusion of well-nourished controls provides an indication of normal ranges in young African children. HIV-positive and HIV-negative SAM groups will be stratified to include approximately 50 children with and without oedematous malnutrition, if possible.

Microbiota and metabolomics substudy

Power calculations are difficult in metagenomics and metabolomic analyses due to the large number of observed outcomes and unknown effect sizes and variance. Previous studies using smaller sample sizes have identified significant taxonomic differences in twin pairs discordant for oedematous-SAM (n=13)[25] and metabolic differences between the two forms of SAM (n=40)[26]. These studies suggest that a difference of 50% in metabolites could be expected. Using ANCOVA, setting α=0.05 and assuming either low (ρ=0.1) or high (ρ=0.7) correlation, the study would require 95-126 subjects to achieve 80% power[61]. False discovery rate (FDR) multiple correction testing will be applied to reduce the high-dimensionality of the data and limit false-positives.

Immunology substudy

Up to 200 children with SAM and 200 well-nourished controls will be included in a cross-sectional analysis of innate immune cell function during hospitalisation. Assuming similar infectious mortality to a recent Kenyan study (15%)[17], a cohort of 200 provides 80% power to detect associations between immune profiles and infectious mortality at an odds ratio of 1.7 and 2-sided alpha of 0.05. We will aim for 100 children with longitudinal analysis of innate immune cell function at discharge, 12, 24 and 48 weeks post-discharge[17].

For peer review only - http://bmjopen.bmj.com/site/about/guidelines.xhtml

STUDY OUTCOMES AND RISK FACTORS

The main study outcomes are clinical (mortality, morbidity and relapse of malnutrition) and nutritional (weight, height, mid-upper arm circumference, leg length, head circumference, mid-thigh circumference, skin-fold thickness and body composition by bioimpedance vector analysis) assessed over 48 weeks of follow-up. Mortality is assessed in hospital by daily physician review and, post-discharge, through study visits and by telephone where possible for children who are lost to follow-up. Morbidity during hospitalization is assessed through daily clinical assessments and available hospital laboratory tests. Morbidity after discharge is assessed, first, using daily morbidity diaries, in which caregivers record episodes of illness (lethargy interfering with feeding; respiratory distress; diarrhoea; oedema and fever); second, from caregiver recall and review of handheld medical records at each follow-up visit; and, third, from data collected during hospitalization for children who are readmitted during the follow-up period. Time-to-recovery from malnutrition will be evaluated during hospitalization; relapse of malnutrition during follow-up will be categorized as moderate acute malnutrition, uncomplicated SAM and complicated SAM, according to WHO definitions. Nutritional outcomes will be expressed both as continuous variables (attained Z-score and change in Z-score between visits), and as categorical variables (moderate wasting, WHZ<-2; severe wasting, WHZ<-3; stunting, HAZ<-2; severe stunting, HAZ<-3; underweight, WAZ <-2; and microcephaly, head circumference-for-age <-2).

Risk factors will be evaluated at baseline, hospital discharge and over the period of follow-up for associations with clinical and nutritional outcomes. In addition to baseline clinical and demographic factors, the following laboratory parameters will be evaluated: haemoglobin, serum albumin, C-reactive protein, CD4 count and HIV viral load (for HIV-

For peer review only - http://bmjopen.bmj.com/site/about/guidelines.xhtml

positive children). Haemoglobin, CD4 and HIV-viral load will be measured in real time and the results reviewed during follow-up clinics.

Data on potential confounders are collected at baseline, discharge and during the follow-up period, including child feeding practices, household socioeconomic status (defined by household income and cooking method), maternal employment and education, and household factors such as water, sanitation and hygiene practices, availability of electricity, location (rural, peri-urban or urban) and household size.

ANALYSIS

All analyses will be interpreted exploratively since HOPE-SAM is an observational study with multiple risk factors, outcomes and substudies. For all analyses, P values will not be artificially adjusted, but interpreted as exploring the strength of evidence supporting any association. The only exception is the use of approaches to minimise false discovery when analysing high-dimensional data from the microbiota and metabolomics substudies, as described.

Observational Cohort

The primary comparison will be the clinical and nutritional outcomes of children with HIV-SAM compared to HIV-negative children with SAM. We will review all deaths and adjudicate clinical diagnoses and causes of death to ensure robust and consistent data across sites. We will compare each participant's clinical management to WHO guidelines to identify any contributory factors in hospital care. Factors associated with outcomes during hospitalisation (e.g. mortality, nutritional recovery) will be determined for each group (HIV-SAM and HIV-negative children with SAM) using multivariable analysis (Cox models for time-to-event data, linear models for continuous outcomes). Factors

For peer review only - http://bmjopen.bmj.com/site/about/guidelines.xhtml

associated with outcomes over 48 weeks post-discharge (hospital re-admission, morbidity and mortality, relapse, anthropometry, body composition and response to ART) will be determined for each group (HIV-SAM and HIV-negative children with SAM) using multivariable analysis (Cox models for time-to-event data, linear models for continuous outcomes). HIV-positive children with SAM and HIV-negative children with SAM will be included in one model together with the risk factors, and interaction tests will be used to investigate whether associations between risk factors and outcomes differ between the two groups of children. We will evaluate the ability of mid-upper arm circumference (MUAC) at discharge to predict long-term outcomes using receiver-operator-characteristic (ROC) analysis, in the whole cohort and within the subgroups of HIV-SAM and HIV-negative children with SAM. We will then evaluate whether addition of other variables improves the predictive capacity of MUAC (using WHO criteria in those >6 months old, and published data for children <6 months[62]) for each group, including body composition, haemoglobin, albumin and CRP, plus CD4%, viral load and timing of ART initiation (HIV-SAM only). We will construct multivariable models and compare them with MUAC alone using the net-reclassification index.

Body composition analysis

Previous work in body composition by bio-electrical impedance in Ethiopian infants and children with SAM has shown that the conventional approach, predicting total body weight from height-adjusted impedance, fails due to confounding by oedema[63]. The same project validated an alternative approach, known as Bio-electrical Impedance Vector Analysis (BIVA), and described significant differences between each of three groups: healthy controls, oedematous-SAM and non-oedematous SAM. Vector analysis splits impedance into two height-adjusted components, resistance and reactance, which are further linked through phase angle (PA). Variability in these components is

For peer review only - http://bmjopen.bmj.com/site/about/guidelines.xhtml

associated with biochemical parameters[64]. These variables will be explored using graphical analysis, or transformed into age- and sex-adjusted Z-scores for statistical comparison, including longitudinal analyses. Higher phase angle indicates better nutritional status, while declining height-adjusted resistance over time indicates loss of oedema.

Enteropathy substudy

The primary comparison for the enteropathy substudy will be between HIV-positive children with SAM (group A) and HIV-negative children with SAM (group C), stratified by presence or absence of oedema. Control groups (B and D) are well-nourished children with or without HIV, to provide normative data for biomarkers and to evaluate the impact of SAM within each HIV group. Thus, biomarkers among HIV-positive children with SAM will first be compared to HIV-negative children with SAM (to evaluate the impact of HIV) and, second, to well-nourished HIV-positive children (to evaluate the impact of SAM). Biomarkers among HIV-negative children with SAM will be compared to well-nourished HIV-negative children. For each continuous outcome, simple descriptive analysis will be used to compare groups during hospitalisation using t-tests on appropriately transformed data. For any outcome with moderate ($p<0.05$) evidence of difference between either group a regression model will be constructed including groups A, B, C, D to directly test (using interactions) whether there is a synergistic effect of HIV-SAM versus HIV-negative SAM versus HIV alone versus neither. These models will also be used to explore whether there is any evidence for heterogeneity in effects between oedematous and non-oedematous SAM. Associations between enrolment factors (e.g. intestinal permeability and microbial translocation) will be explored using pairwise Spearman correlations and principal components analysis. Mean changes at the follow-up time-points in each group will be estimated, and groups compared (as above) using

For peer review only - http://bmjopen.bmj.com/site/about/guidelines.xhtml

generalised estimating equations. For outcomes that differ across SAM groups over time, multilevel models will be used to explore possible predictors from the other factors measured. Time to nutritional recovery will be compared using Kaplan-Meier and log-rank tests, and Cox models to adjust for baseline differences between groups.

Microbiota and metabolomics substudy

The primary comparison will be between HIV-negative children with oedematous and non-oedematous SAM, with a separate comparison between HIV-positive children with SAM and HIV-negative children with SAM. Analyses will examine: (i) differences in metagenomic/metabolomic variables between groups at each time-point; (ii) differences in metagenomic/metabolomic variables within groups over time; (iii) correlations between metagenomic and metabolomic variables; and (iv) correlations between metagenomic/metabolomic variables and clinical outcomes. A systematic analysis will be undertaken to reduce high-dimensional data, integrate the multi-omics datasets and minimise false discovery.

Compositional metagenomic data will be compared between groups for indices of alpha and beta diversity. Principal coordinate analysis and partial least squares discriminant analysis will be performed on metabolomics data to identify overall differences between groups. High-dimensional datasets will be reduced using random forest models to identify taxa, microbial gene families and metabolites that most strongly contribute to differences between groups, corrected by Benjamani-Hochburg false discovery rate detection. Targeted analysis by qRT-PCR will validate differential abundance or expression of candidate microbial genes. Longitudinal comparisons will be performed within and between groups using multilevel simultaneous component analysis. Orthogonal projections to latent structures models will integrate metabolomic and

For peer review only

metagenomic data whilst linear regression, canonical correlation and hierarchal clustering analysis will measure correlations between -omics datasets. Finally, ROC analysis will identify the ability of different analytes to predict long-term nutritional and clinical outcomes.

Immunology substudy

Integrated profiles of innate immune cell function will be generated for each child using principal components analysis followed by hierarchical clustering[65 66]. This data-reduction method identifies whether absolute levels of specific markers or relative differences between markers differentiate children into groups. The resulting innate immune profiles will be compared between HIV-SAM, HIV-negative children with SAM and well-nourished groups using univariable tests and multivariable analysis of variance (MANOVA) of the principal components.

To address the relationship between immune function and infections, regression analyses will determine whether baseline innate immune profiles (or the individual parameters defining them) are associated with the infectious morbidity or mortality during hospitalisation, using logistic models for binary outcomes and linear models for duration. Key clinical characteristics, including age, sex, oedema and baseline WHZ, will be added to models to investigate their confounding effects. Multivariable stacked regression methods will be used to compare the impact of different factors on severe bacterial infections based on heterogeneity tests.

To determine whether treatment for SAM restores innate immune cell antibacterial function, mixed effects regression models will compare longitudinal changes in individual immune parameters, and the principal components calculated from the weights identified

For peer review only - http://bmjopen.bmj.com/site/about/guidelines.xhtml

at baseline (which include well-nourished controls). Similarities and differences in longitudinal immune profiles will be compared between groups using nonmetric multi-dimensional scaling[65 67 68]. This approach will group children according to their composite innate immune function, allowing the duration and variability of immune restoration to be evaluated over the course of nutritional rehabilitation. Binary logistic regression will determine whether innate immune profiles at discharge are associated with morbidity or mortality during follow-up.

PATIENT AND PUBLIC INVOLVEMENT

Patients and their caregivers were not involved in the design of the study. During recruitment, all caregivers of children admitted to hospital were given information about the study; those whose children had severe acute malnutrition were approached to give written informed consent. A meeting to disseminate results of the study to participants and their caregivers will be held at the end of the study. An interactive game to engage caregivers in the science underlying malnutrition is being developed in collaboration with experts from the Centre of the Cell, a unique science education centre based at Queen Mary University of London (https://www.centreofthecell.org/).

SAFETY REPORTING

For all adverse events, the study team will assess expectedness and relatedness to study activities. Since this is an observational study without interventions, we anticipate that the risk is minimal; however, serious adverse events will be reported to local ethical review boards (Medical Research Council of Zimbabwe, and University of Zambia Biomedical Research Ethics Committee) and the study sponsor (Queen Mary University of London) according to their respective guidelines.

For peer review only - http://bmjopen.bmj.com/site/about/guidelines.xhtml

DATA COLLECTION AND MONITORING

Clinical and demographic data are recorded on paper case report forms. All data are checked for completeness and plausibility before data entry and problems flagged for resolution by the clinical team. All data are double-entered onto a dedicated password-protected online study database, and any discrepancies resolved. Study participants are identified on electronic databases only by study numbers (assigned at enrolment); no personal identifiers are entered.

ETHICS AND DISSEMINATION

The study complies with the principles of the Declaration of Helsinki (2008) and is conducted in compliance with the principles of Good Clinical Practice (GCP) and local regulatory requirements in each country. Ethical approval was obtained from the University of Zambia Biomedical Research Ethics Committee, the Joint Research Ethics Committee of the University of Zimbabwe and the Medical Research Council of Zimbabwe. The ethical review board of the Sponsor, Queen Mary University of London, provided an advisory review of the study. Since this is an observational study, there is no Data and Safety Monitoring Board.

Results will be disseminated through conference abstracts and peer-reviewed publications and discussed with relevant policymakers and programmers. Study findings will be disseminated to families of participants at face-to-face meetings.

TIME FRAME AND STUDY STATUS

For peer review only

Enrolment into the study began in July 2016 and is expected to end in March 2018. All participants will be followed for 48 weeks, with an expected study completion date of March 2019.

DISCUSSION

HOPE-SAM aims to document the short- and long-term clinical and nutritional outcomes of HIV-positive and HIV-negative children with SAM, and to identify the factors at presentation and at discharge from hospital that independently predict these outcomes. Mechanistic substudies aim to evaluate the contribution of enteropathy, microbiota, metabolome and innate immune cell function to these clinical outcomes. The prevalence of malnutrition in HIV-positive children is as high as 40% in some settings and the challenges of managing this population are well recognised[69]. The WHO protocol on management of SAM aims to reduce case fatality below 10%, but rates as high as 35% are still reported among HIV-positive children[5 70]. No studies have systematically and longitudinally collected morbidity data in HIV-SAM, or documented repeat hospitalisations and mortality after discharge from hospital, particularly in the current era where ART is available upon diagnosis. HOPE-SAM will provide a unique opportunity to enrol and follow a cohort of children managed for SAM in three large hospitals across two sub-Saharan African countries at several time-points over a one-year period. Nested longitudinal laboratory substudies aim to better characterise the pathogenesis of SAM in HIV-positive and HIV-negative children, to determine whether pathogenic processes are normalised during nutritional rehabilitation and follow-up, and to identify potential mechanistic pathways. Our ultimate goal is to utilise the findings generated in this study to inform new intervention approaches that can be evaluated in clinical trials to improve outcomes among children with SAM.

For peer review only - http://bmjopen.bmj.com/site/about/guidelines.xhtml

ACKNOWLEDGEMENTS

Members of the HOPE-SAM study team not listed in the author list: *Harare, Zimbabwe:* Virginia Sauramba, Adlight Dandadzi, Chipo Kureva, Johnson Mushonga, Eddington Mpofu, Washington Dune, Tafadzwa Chidhanguro, Sibongile Nkiwane, Sandra Rukobo, Margaret Govha, Patience Mashayanembwa, Leah Chidamba, Bernard Chasekwa, Joice Tome, Rachel Makasi, Wellington Murenjekekwa, Theodore Chidawanyika, Blessing Tsenesa, Stephen Moyo, Penias Nyamwino, Pururudzai Simango, Shepherd Seremwe, Lovemore Chingaoma and Sarudzai Kasaru. *Lusaka, Zambia*: Andreck Tembo, Mary Mpundu, Evelyn Nyendwa, Gwendolyn Nayame, Dalitso Tembo, Sophreen Mwaba, Esther Chilala, Lucy Macwani, Tenzeni Dumba, Miyoba Chipunza, Lydia Kazhila, Temwaninge Gondwe, Dennis Phiri, Mpala Mwanza, Kanekwa Zyambo.

We thank Philippa Rambanepasi, Karen Gwanzura and Agatha Muyenga for financial management of the study; Zinah Sorefan and Daniela Azurunwa for study coordination at Queen Mary University of London; Professor Kim Michaelsen for the use of the electronic knemometers and advice on their use; Dr Gemma Buxton for her help in drafting the case report forms used in the study; and we remember the hard work of Edith Mukusho who sadly passed away during the HOPE-SAM study. We thank the staff and management and members of the Department of Paediatrics and Child Health at the three hospital sites who have made this study possible. We are indebted to the caregivers, families and children who are participating in the HOPE-SAM study.

For peer review only

AUTHOR CONTRIBUTIONS

Designed study: MB-D, BA, CDB, RCR, BM, KC, CK, KCh, DN, PC, NC, FM, JW, ARM, JS, ASW, KJN, PK, AJP

Sought funding: MB-D, BA, CDB, RCR, JHH, ARM, JS, ASW, KJN, PK, AJP

Undertaking study: BM, KC, CK, KCh, FM, DN, PC, NC, FM, IM, EB, KM, SM, TR

Study oversight: MB-D, BA, JHH, KJN, PK, AJP

Analysis: MB-D, BA, CDB, RCR, RN, JW, ARM, JS, ASW, KJN, PK, AJP

Wrote first draft of manuscript: MB-D, CDB, RCR, AJP

Critically revised manuscript: All

FUNDING

This work was supported by the Medical Research Council UK (MR/K012711/1), the Wellcome Trust (107634/Z/15/Z to MB-D; 206225/Z/17/Z to CDB, an award funded in partnership with the Royal Society; 206455/Z/17/Z to RCR; and 108065/Z/15/Z to AJP), and a Bio-Resource Grant from the Centre for Genomic Health within the Life Sciences Initiative at Queen Mary University of London.

COMPETING INTERESTS

None of the authors have any competing interests to declare.

For peer review only

For peer review only

References

1. Black R E, Victora C G, Walker S P, et al. Maternal and child undernutrition and overweight in low-income and middle-income countries. *Lancet* 2013;382:427–51.
2. World Health Organisation. Guideline Updates on the management of severe acute malnutrition in infants and children: World Health Organisation, Geneva, 2013. Available at http://apps.who.int/iris/handle/10665/ 95584
3. Lenters LM, Wazny K, Webb P, et al. Treatment of severe and moderate acute malnutrition in low- and middle-income settings: a systematic review, meta-analysis and Delphi process. *BMC Public Health* 2013;13 (Supp 3):S23.
4. Golden MHN. Oedematous malnutrition. *British Medical Bulletin* 1998;54(2):433-44.
5. Schofield C, A. A. Why have mortality rates for severe malnutrition remained so high? *Bull World Health Organ* 1996;74(2):223-29.
6. Munthali T, Jacobs C, Sitali L, et al. Mortality and morbidity patterns in under-five children with severe acute malnutrition (SAM) in Zambia: a five-year retrospective review of hospital-based records (2009–2013). *Archives of Public Health* 2015;73(23)
7. Preidis G A, McCollum E D, Mwansambo C, et al. Pneumonia and Malnutrition are Highly Predictive of Mortality among African Children Hospitalized with Human Immunodeficiency Virus Infection or Exposure in the Era of Antiretroviral Therapy. *Journal of Pediatrics* 2011;159(3):484-89.
8. World Health Organisation G. Global guidance on criteria and processes for validation: Elimination of Mother-to-Child transmission of HIV and Syphilis. Geneva: World Health Organization, 2017. Available at https://www.who.int/reproductivehealth/publications/emtct-hiv-syphilis/en/
9. Evans C, Jones CE, Prendergast AJ. HIV-exposed, uninfected infants: new global challenges in the era of paediatric HIV elimination. *Lancet Infectious Diseases* 2016; 16(6): e92-e107
10. Heikens GT, Bunn J, Amadi A, et al. Case management of HIV-infected severely malnourished children: challenges in the area of highest prevalence. *Lancet* 2008;371:1305-7.
11. Kerac M, Bunn J, Chagaluka G, et al. Follow-Up of Post-Discharge Growth and Mortality after Treatment for Severe Acute Malnutrition (FuSAM Study): A Prospective Cohort Study. *Plos One* 2014;9(6): e96030
12. Lelijveld M, Seal A, Wells J C, et al. Chronic disease outcomes after severe acute malnutrition in Malawian children (ChroSAM): a cohort study. *Lancet Global Health* 2016;4:e654-62.
13. Moïsi JC, Gatakaa H, Berkley JA, et al. Excess child mortality after discharge from hospital in Kilifi, Kenya: a retrospective cohort analysis. *Bull World Health Organ* 2011;89:725-32A.

For peer review only - http://bmjopen.bmj.com/site/about/guidelines.xhtml

14. Wiens MO, Pawluk S, Kissoon N, et al. Pediatric Post-Discharge Mortality in Resource Poor Countries: A Systematic Review. *Plos One* 2013;8(6): e66698
15. Ngari MM, Fegan G, Mwangome MK, et al. Mortality after Inpatient Treatment for Severe Pneumonia in Children: a Cohort Study. *Paediatric and Perinatal Epidemiology* 2017;31:233-42.
16. Fergusson P, Chinkhumba J, Grijalva-Eternod C, et al. Nutritional recovery in HIV-infected and HIVuninfected children with severe acute malnutrition. *Archives of Diseases in Children* 2009;94:512-16.
17. Berkley JA, Ngari M, Johnstone T, et al. Daily co-trimoxazole prophylaxis to prevent mortality in children with complicated severe acute malnutrition: a multicentre, double-blind, randomised placebo-controlled trial. *Lancet Global Health* 2016;4(7):e464 - e73
18. DeBoer M D, Lima A A M, Oría R B, et al. Early childhood growth failure and the developmental origins of adult disease: Do enteric infections and malnutrition increase risk for the metabolic syndrome? *Nutrition Reviews* 2012 70(11):642-53.
19. Barouki R, Gluckman P D, Grandjean P, et al. Developmental origins of non-communicable disease: Implications for research and public health. *Environmental Health* 2012;11:42
20. Davey Smith G, Greenwood R, Gunnell D, et al. Leg length, insulin resistance, and coronary heart disease risk: The Caerphilly Study. *Journal of Epidemiology Community Health* 2001;55:867-72.
21. Gunnell D, Whitley E, Upton MN, et al. Associations of height, leg length, and lung function with cardiovascular risk factors in the Midspan Family Study. *Journal of Epidemiology Community Health* 2002;57:141-46.
22. Jones K D, Thitiri J, Ngari M, et al. Childhood malnutrition: Toward an understanding of infections, inflammation, and antimicrobials. *Food and Nutrition Bulletin* 2014;35(2 Suppl): S64-70
23. Attia S, Versloot CJ, Voskuijl W, et al. Mortality in children with complicated severe acute malnutrition is related to intestinal and systemic inflammation: an observational cohort study. *American Journal of Clinical Nutrition* 2016;104:1441-9.
24. Bourke CD, Berkley J A, Prendergast AJ. Immune Dysfunction as a Cause and Consequence of Malnutrition. *Trends in Immunology* 2016;37(6):386-89.
25. Smith MI, Yatsunenko T, Manary MJ, et al. Gut microbiomes of Malawian twin pairs discordant for kwashiorkor. *Science* 2013;339(6119):548-54.
26. Di Giovanni V, Bourdon C, Wang DX, et al. Metabolomic Changes in Serum of Children with Different Clinical Diagnoses of Malnutrition. *Journal of Nutrition* 2016;146:2436-44.
27. Rytter MJH, Kolte L, Briend A, et al. The Immune System in Children with Malnutrition—A Systematic Review. *Plos One* 2014;9(8):e105017
28. Blanton LV, Charbonneau MR, Salih T, et al. Gut bacteria that prevent growth impairments transmitted by microbiota from malnourished children. *Science* 2016;351(6275)
29. Subramanian S, Huq S, Yatsunenko T, et al. Persistent gut microbiota immaturity in malnourished Bangladeshi children. *Nature* 2014;510(7505):417-21.

For peer review only - http://bmjopen.bmj.com/site/about/guidelines.xhtml

30. Prendergast A, Kelly P. Review: Enteropathies in the developing world: neglected effects on global health. *Am J Trop Med Hyg* 2012;86:756-63.
31. Lutwama F, Kagina BM, Wajja A, et al. Distinct T-Cell Responses When BCG Vaccination Is Delayed From Birth to 6 Weeks of Age in Ugandan Infants. *The Journal of Infectious Diseases* 2014;209(6):887-97.
32. Prendergast AJ, Humphrey JH, Mutasa K, et al. Assessment of Environmental Enteric Dysfunction in the SHINE Trial: Methods and Challenges. *Clin Infect Dis* 2015;61 (suppl 7):S685-702.
33. World Health Organisation. Management of severe malnutrition : a manual for physicians and other senior health workers. WHO, 1999. Available at http://apps.who.int/iris/handle/10665/41999
34. World Health Organisation. Consolidated guidleines on the use of antiretroviral drugs for treating and preventing HIV infection: Recommendations for a public health approach. WHO, 2016. Available at http://www.who.int/hiv/pub/arv/arv-2016/en/
35. Costiniuk CT, JB. A. Human immunodeficiency virus and the gastrointestinal immune system: does highly active antiretroviral therapy restore gut immunity? *Nature* 2012;5(6):596-604.
36. Brenchley JM, Douek DC. HIV infection and the gastrointestinal immune system. *Nature* 2008;1(1) doi: doi:10.1038/mi.2007.1
37. Amadi B, Besa E, Zyambo K, et al. Impaired Barrier Function and Autoantibody Generation in Malnutrition Enteropathy in Zambia. *EBioMedicine* 2017;22(Supplement C):191-99.
38. Kau AL, Ahern PP, Griffin NW, et al. Human nutrition, the gut microbiome and the immune system. *Nature* 2011;474(7351):327-36.
39. Kau AL, Planer JD, Liu J, et al. Functional characterization of IgA-targeted bacterial taxa from undernourished Malawian children that produce diet-dependent enteropathy. *Sci Transl Med* 2015;7(276):276ra24.
40. Monaco CL, Gootenberg DB, Zhao G, et al. Altered Virome and Bacterial Microbiome in Human Immunodeficiency Virus-Associated Acquired Immunodeficiency Syndrome. *Cell Host Microbe* 2016;19(3):311-22.
41. Amadi B, Fagbemi AO, Kelly P, et al. Reduced production of sulfated glycosaminoglycans occurs in Zambian children with kwashiorkor but not marasmus. *Am J Clin Nutr* 2009;89(2):592-600.
42. Segata N, Waldron L, Ballarini A, et al. Metagenomic microbial community profiling using unique clade-specific marker genes. *Nature methods* 2012;9(8):811-4.
43. Abubucker S, Segata N, Goll J, et al. Metabolic reconstruction for metagenomic data and its application to the human microbiome. *PLoS computational biology* 2012;8(6):e1002358.
44. Bartz S, Mody A, Hornik C, et al. Severe Acute Malnutrition in Childhood: Hormonal and Metabolic Status at Presentation, Response to Treatment and Predictors of Mortality. *Journal of Clinical and Endocrine Metabolism* 2014;99(6):2128-37.

For peer review only - http://bmjopen.bmj.com/site/about/guidelines.xhtml

45. Farras M, Chandwe K, Mayneris-Perxachs J, et al. Characterizing the metabolic phenotype of intestinal villus blunting in Zambian children with severe acute malnutrition and persistent diarrhoea. *Plos One* 2018;13(3):e0192092
46. Mayneris-Perxachs J, Lima AA, Guerrant RL, et al. Urinary N-methylnicotinamide and β-aminoisobutyric acid predict catch-up growth in undernourished Brazilian children. *Sci Rep* 2016;6:19780. doi: 10.1038/srep19780
47. Manary MJ, Leeuwenburgh C, Heinecke JW. Increased oxidative stress in kwashiorkor. *J Pediatr* 2000;137(3):421-4.
48. Chisti MJ, Salam MA, Bardhan PK. Severe Sepsis in Severely Malnourished Young Bangladeshi Children with Pneumonia: A Retrospective Case Control Study. *Plos One* 2015;10(10):e0139966.
49. Page A-L, de Rekeneire N, Sayadi S, et al. Infections in children admitted with complicated severe acute malnutrition in Niger. *Plos One* 2013;8(7):e68699
50. Jones KDJ, A BJ. Severe acute malnutrition and infection. *Paediatrics and International Child Health* 2014;34(sup 1):S1-S29.
51. Bahwere P, Levy J, Philippe H, et al. Community-Acquired Bacteremia among Hospitalized Children in Rural Central Africa. *International Journal of Infectious Diseases* 2001;5:180-88.
52. Thuo N, Ohuma E, Karisa J, et al. The prognostic value of dipstick urinalysis in children admitted to hospital with severe malnutrition. *Archives of Diseases in Childhood* 2010;95(6):422-6.
53. Berkley J A, Lowe BS, Mwangi I, et al. Bacteremia among Children admitted to a rural Hospital in Kenya. *New England Journal of Medicine* 2005;352:39-47.
54. Chang CY, Trehan I, Wang R J, et al. Children Successfully Treated for Moderate Acute Malnutrition Remain at Risk for Malnutrition and Death in the Subsequent Year after Recovery. *Journal of Nutrition* 2013;143:215-20.
55. Ibrahim MK, Zambruni M, Melby CL, et al. Impact of Childhood Malnutrition on Host Defense and Infection. *Clinical Microbiology Reviews* 2017;30(4):919-71.
56. Hughes SM, Amadi B, Mwiya M, et al. Dendritic cell anergy results from endotoxemia in severe malnutrition. *Journal of Immunology* 2009;183(4):2818-26.
57. Jones K. D, Hünten-Kirsch B, Laving AM, et al. Mesalazine in the initial management of severely acutely malnourished children with environmental enteric dysfunction: a pilot randomized controlled trial. *BMC Med* 2014;12 doi: 10.1186/s12916-014-0133-2
58. Watanabe K, A. PJW. Environmental Enteropathy: Elusive but Significant Subclinical Abnormalities in Developing Countries. *EBioMedicine* 2016;10:25-32.
59. Kelly P, Besa E, Zyambo K. Endomicroscopic and Transcriptomic Analysis of Impaired Barrier Function and Malabsorption in Environmental Enteropathy. . *Plos Neglected Tropical Diseases* 2016;10(4):e00046000.
60. Behrens RH, Lunn PG, Northrop CA, et al. Factors affecting the integrity of the intestinal mucosa of Gambian children. *American Journal of Clinical Nutrition* 1987;45:1433-41.

For peer review only - http://bmjopen.bmj.com/site/about/guidelines.xhtml

61. Borm GF, Fransen J, WA L. A simple sample size formula for analysis of covariance in randomized clinical trials. *Journal of Clinical Epidemiology* 2007;60(12):1234-8.
62. Mwangome M, Ngari M, Fegan G, et al. Diagnostic criteria for severe acute malnutrition among infants aged under 6 mo. *The American journal of clinical nutrition* 2017;105(6):1415-23.
63. Girma T, Kæstel P, Workeneh N, et al. Bioimpedance index for measurement of total body water in severely malnourished children: Assessing the effect of nutritional oedema. *Clinical Nutrition* 2016; 35(3):713-7.
64. Girma T, Hother Nielsen AL, Kæstel P, et al. Biochemical and anthropometric correlates of bio-electrical impedance parameters in severely malnourished children: A cross-sectional study. *Clinical Nutrition* 2017; 37(2):701-705.
65. Bourke CD, Nausch N, Rujeni N, et al. Integrated Analysis of Innate, Th1, Th2, Th17, and Regulatory Cytokines Identifies Changes in Immune Polarisation Following Treatment of Human Schistosomiasis. *The Journal of Infectious Diseases* 2013;208(1):159-69.
66. Prendergast AJ, Szubert AJ, Berejena C, et al. Baseline Inflammatory Biomarkers Identify Subgroups of HIV-Infected African Children With Differing Responses to Antiretroviral Therapy. *Journal of Infectious Diseases* 2016;214:226-36
67. Bourke CD, Mutapi F, Nausch N, et al. Trichuris suis ova therapy for allergic rhinitis does not affect allergen-specific cytokine responses despite a parasite-specific cytokine response. *Clinical and Experimental Allergy* 2012;42:1582-95.
68. Bourke CD, Nausch N, Rujeni N, et al. Cytokine Responses to the Anti-schistosome Vaccine Candidate Antigen Glutathione-S-transferase Vary with Host Age and Are Boosted by Praziquantel Treatment. *Plos Neglected Tropical Diseases* 2014;8(5)
69. Fergusson P, Tomkins A. HIV prevalence and mortality among children undergoing treatment for severe acute malnutrition in sub-Saharan Africa: a systematic review and meta-analysis. *Transactions of the Royal Society of Tropical Medicine and Hygiene* 2009;103(6):541-48.
70. Chinkhumba J, Tomkins A, Banda C, et al. The impact of HIV on mortality during in-patient rehabilitation of severely malnourished children in Malawi. *Transactions of the Royal Society of Tropical Medicine and Hygiene* 2008;102(7):639-44.

For peer review only - http://bmjopen.bmj.com/site/about/guidelines.xhtml

FIGURE LEGENDS

Figure 1: **Study flow chart.**

All hospital admissions are screened for eligibility for the observational cohort and enteropathy sub-study, with procedures undertaken as shown in the flow chart during hospitalisation and post-discharge. Well-nourished children from outpatient clinics and the community meeting eligibility criteria as well-nourished controls are enrolled and undergo a single baseline assessment as shown. The immunology, microbiota and metabolomics sub-studies enrol children as shown. All children with SAM, regardless of which arm of the study they are enrolled into, are followed for 48 weeks post-discharge. [1]The immunology substudy started from 1st June 2017 and required children to have a blood sample >2mL to conduct cellular assays.

For peer review only

For peer review only - http://bmjopen.bmj.com/site/about/guidelines.xhtml

For peer review only

For peer review only - http://bmjopen.bmj.com/site/about/guidelines.xhtml

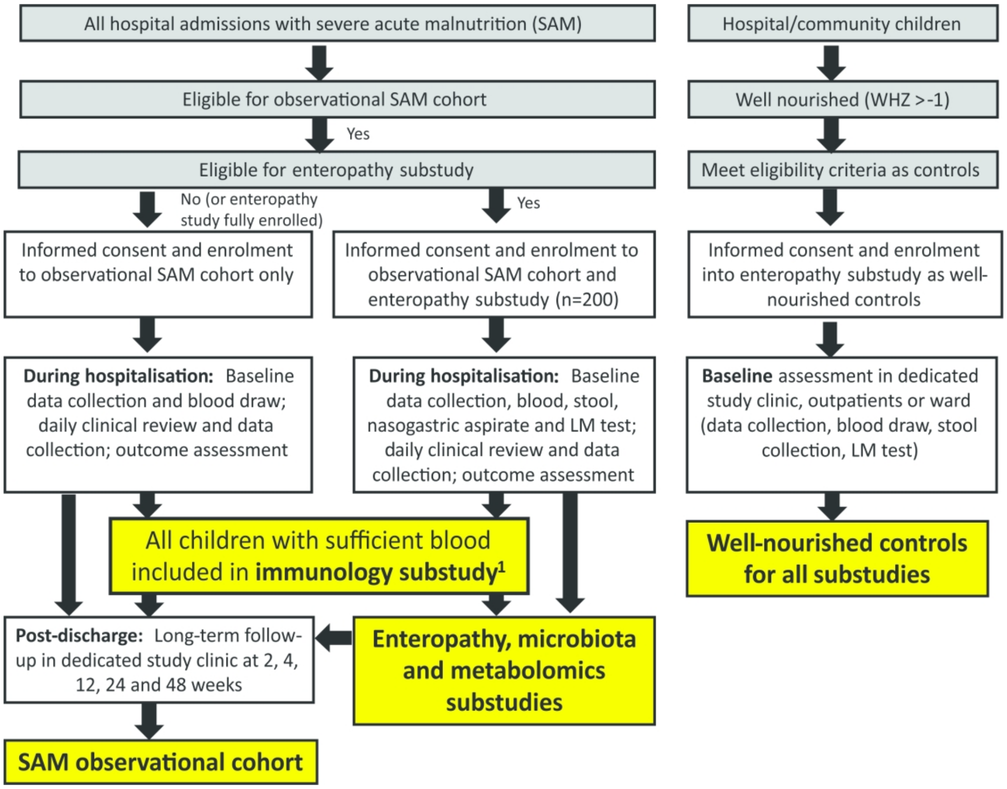

204x159mm (300 x 300 DPI)

Supplementary Table 1: Assays undertaken on stored samples for children in the observational cohort and immunology substudy.

Sample type	Assay (method)	Location of work	Study subjects	Baseline	Discharge	Week 12	Week 24	Week 48
Blood	HIV testing (rapid antibody test algorithm if >18 mo old or HIV DNA PCR <18 mo old)[1]	TROPGAN, Zvitambo or clinical sites	All	X				
Blood	CD4 count (flow cytometry or PIMA)[1]	TROPGAN, Zvitambo or clinical sites	All HIV-positive	X	X	X	X	X
Plasma	HIV viral load (real-time PCR)[1]	TROPGAN, Zvitambo or clinical sites	All HIV-positive	X	X	X	X	X
Plasma	C-reactive protein (ELISA)	TROPGAN, Zvitambo	All	X	X	X	X	X
Plasma	Albumin (ELISA)	TROPGAN, Zvitambo	All	X	X	X	X	X
Plasma	Lipopolysaccharide (LAL assay)	TROPGAN and Zvitambo	Subgroup[3]	X	X	X	X	X
Plasma	Lipopolysaccharide binding protein (LBP)	TROPGAN and Zvitambo	Subgroup[3]	X	X	X	X	X
Plasma	sCD14 (ELISA)	TROPGAN and Zvitambo	Subgroup[3]	X	X	X	X	X
Plasma	sCD163 (ELISA)	TROPGAN and Zvitambo	Subgroup[3]	X	X	X	X	X
Plasma	IL-6, TNF-alpha, IL-1β (ELISA) and/or multiplex cytokines	TROPGAN and Zvitambo	Subgroup[3]	X	X	X	X	X
Plasma	Total PAMP activity (THP1 reporter cell line[2])	TROPGAN and Zvitambo	Subgroup[3]	X	X	X	X	X
Whole	Molecular techniques	QMUL, London[2]	Subgroup[3]	X	X	X	X	X

For peer review only - http://bmjopen.bmj.com/site/about/guidelines.xhtml

blood	for bacterial detection (broad-range and specific PCR and next-generation sequencing)							
**Immunology substudy only**								
Whole blood	*In vitro* binding to bacterial products, cytokine expression and cellular responses to PAMP stimulation[4]	TROPGAN, Zvitambo	All	X	X	X	X	X
Plasma	Co-culture with healthy immune cells[5]	QMUL, London	All	X	X	X	X	X

[1]If HIV test, CD4 and viral load have already been conducted as part of routine clinical care, they will not be repeated on the research sample.
[2]THP1 reporter cells are derived from THP1, a human monocytic cell line that naturally expresses many pattern recognition receptors (PRR). The cell line stably expresses an NF-κB/AP-1 inducible reporter (SEAP) system to facilitate the monitoring of PRR-induced NF-κB/AP-1 activation.
[3]Assays will be undertaken in a subgroup of children, using a case-control or case-cohort design to evaluate the impact of biomarkers on immune activation and mortality.
[4]Whole blood will be stimulated with pathogen-associated molecular patterns (PAMP) in culture plates and bacterial antigens labelled with fluorescent tags in test tubes, and incubated for 1-24hr. Supernatant will be removed and stored at -80C for subsequent analysis of pro- and anti-inflammatory cytokines, and cells will be fixed for subsequent analysis of bacterial binding, cellular activation, proliferation and cytokine elaboration by flow cytometry.
[5]To determine the effect of the systemic milieu on healthy immune cell function, plasma samples will be transported to the Blizard Institute, QMUL and co-cultured with healthy immune cells, which will be functionally analysed via multi-parameter flow cytometry in the Flow Cytometry Core Facility.

IFABP: Intestinal fatty acid binding protein; ELISA: Enzyme-linked immunosorbent assay; GLP-2: glucagon-like peptide 2; sCD14: soluble CD14; sCD163: soluble CD163; PAMP: pathogen-associated molecular pattern; QMUL: Queen Mary University of London; CRP: C-reactive protein; LAL: limulus amoebocyte lysate assay.

For peer review only - http://bmjopen.bmj.com/site/about/guidelines.xhtml

Supplementary table 2: Additional laboratory analyses for enteropathy substudy

Sample type	Assay (method)	Location of work	Study groups[1]	Baseline	Discharge	Week 12	Week 24	Week 48
Urine	Lactulose-mannitol ratio (mass spectrometry)	Orgeon Analytics, USA	A, B, C, D (all)	X	X	X		X
Stool	Neopterin, myeloperoxidase, alpha-1 antitrypsin and REG-1B (ELISA)	TROPGAN and Zvitambo	A, B, C, D (all)	X	X	X	X	X
Plasma	I-FABP (ELISA)	TROPGAN and Zvitambo	A, B, C, D (all)	X	X	X	X	X
Plasma	GLP-2	TROPGAN and Zvitambo	A, B, C, D (all)	X	X	X	X	X
Plasma	Citrulline (mass spectrometry)	Imperial College London	A, B, C, D (all)	X	X	X	X	X
Plasma	Kynurenine:tryptophan ratio and metabolites along tryptophan pathway (mass spectrometry)	Imperial College London	A, B, C, D (all)	X	X	X	X	X
Stool	Microbiome analysis[2]	BCCDC, Vancouver	A, B, C, D (all)	X	X	X	X	X
Stool	*Helicobacter pylori* antigen	TROPGAN and Zvitambo	A, B, C, D (all)	X				
Gastric juice	Culture and molecular techniques for bacterial detection (broad-range and specific PCR and next-generation sequencing)	QMUL, London	Subgroup of A, C (n=50 per group)	X				

For peer review only - http://bmjopen.bmj.com/site/about/guidelines.xhtml

RNA extracted from PAXGene tubes	Gene expression analysis (RNASeq)	QMUL, London	A, B, C, D (all)	X	X			
Plasma and urine	Targeted and untargeted metabolic phenotyping	Imperial College London	A, B, C, D (all)	X	X	X	X	X

[1]Enteropathy substudy groups: Group A:,HIV-positive children with severe acute malnutrition; Group B: HIV-positive well-nourished controls; Group C: HIV-negative children with severe acute malnutrition; Group D: HIV-negative well-nourished controls.

Note that controls only have blood taken at baseline.

[2]For microbiome analyses,total DNA and/or RNA will be extracted from stool samples and used as template for next generation sequencing library preparation and for quantitative polymerase chain reaction (qPCR), then sequenced via whole metagenome shotgun sequencing

QMUL: Queen Mary University of London; BCCDC: British Columbia Centre for Disease Control.

For peer review only
